# Combining Mendelian randomization and network deconvolution for inference of causal networks with GWAS summary data

**DOI:** 10.1371/journal.pgen.1010762

**Published:** 2023-05-18

**Authors:** Zhaotong Lin, Haoran Xue, Wei Pan

**Affiliations:** Division of Biostatistics, University of Minnesota, Minneapolis, Minnesota, United States of America; Huazhong University of Science and Technology Tongji Medical College, CHINA

## Abstract

Mendelian randomization (MR) has been increasingly applied for causal inference with observational data by using genetic variants as instrumental variables (IVs). However, the current practice of MR has been largely restricted to investigating the *total* causal effect between two traits, while it would be useful to infer the *direct* causal effect between any two of many traits (by accounting for indirect or mediating effects through other traits). For this purpose we propose a two-step approach: we first apply an extended MR method to infer (i.e. both estimate and test) a causal network of total effects among multiple traits, then we modify a graph deconvolution algorithm to infer the corresponding network of direct effects. Simulation studies showed much better performance of our proposed method than existing ones. We applied the method to 17 large-scale GWAS summary datasets (with median *N* = 256879 and median #IVs = 48) to infer the causal networks of both total and direct effects among 11 common cardiometabolic risk factors, 4 cardiometabolic diseases (coronary artery disease, stroke, type 2 diabetes, atrial fibrillation), Alzheimer’s disease and asthma, identifying some interesting causal pathways. We also provide an R Shiny app (https://zhaotongl.shinyapps.io/cMLgraph/) for users to explore any subset of the 17 traits of interest.

## Author summary

Understanding the causal relationship and building a causal network among several risk factors and diseases is a key to therapeutic development and informed clinical decision making. The development of Mendelian randomization (MR) opens a door to using observational GWAS summary data for causal inference between a pair of traits. Here we first take advantage of bi-directional Mendelian randomization to infer the total causal effect between each pair of traits in the network without specifying an exposure and an outcome a priori. Then we apply network deconvolution to the (estimated) total causal network, obtaining an estimated direct causal network, each edge of which represents the direct causal effect of one trait on another that is not mediated through any other traits. On the other hand, most MR methods are vulnerable to violation of valid instrumental variable assumptions and are based on two independent samples while GWAS sample overlap becomes inevitable as more large-scale consortium data being used. Therefore we propose a robust and efficient MR method, called MR-cML-C, accommodating overlapping samples, and show that it has nice statistical properties. We also clarify both finite-sample and large-sample properties of the causal parameter estimator under the incorrect independence assumption (i.e. ignoring sample overlap). By reconstructing both a total and a direct causal networks of 17 traits, including 11 common cardiometabolic risk factors and 6 diseases, we demonstrate the usefulness of our method.

## Introduction

A fundamental task in science is to understand causal pathways among various risk factors and diseases. This is particularly challenging with observational data due to the likely presence of hidden confounding, implying that an observed association is not equivalent to a causation. In our real data example, we’d like to infer which of some known risk factors are causal to coronary artery disease (CAD). While many previous studies have established for example that obesity is associated with CAD [1], whether it is causal, especially independent of other known risk factors, is still debatable with conflicting results from observational studies [2]. Mendelian randomization (MR) is a powerful tool to infer causal relationship between two traits in the presence of unmeasured confounding, by using single nucleotide polymorphisms (SNPs) as instrumental variables (IVs) [3–5]. A distinct and useful feature of MR is its applicability when the two traits come from two different genome-wide association study (GWAS) summary datasets. The conventional MR analysis usually assumes the causal direction is known from an exposure to an outcome. When the direction is not clear, bidirectional MR can be applied [6, 7]. However, such a causal estimate only reflects the *total* causal effect from one trait to the other, which consists of possibly both a direct effect and an indirect effect mediated through other factors [8–11]. In our motivating real data example, we’d like to estimate causal relationships among multiple common risk factors and diseases; we are not only interested in a total effect of a risk factor, say obesity/BMI, on a disease, say CAD, but also its direct effect after accounting for possible mediating effects through other risk factors. In addition, in general we do not want to pre-specify any causal directions because, for example, there may be a bidirectional relationship between BMI and CAD. For this purpose, we propose a two-step framework to infer both total and direct causal networks, allowing bi-directional relationships (i.e. cycles). In the first step, we apply bidirectional MR on every pair of traits to construct a *total* causal (effect) graph, depicting the total causal effect from one node to the other. In the second step, we apply network deconvolution [12] to the (estimated) total causal network to estimate the *direct* causal (effect) graph, each edge of which measures the direct effect of one node on the other after accounting for mediating effects through other nodes in the graph.

In principle, any bidirectional MR method could be used in the first step. However, the inference of the direct causal graph depends crucially on the validity of the estimated total causal effects in the first step, which relies on the three key IV assumptions in MR: (i) Relevance assumption—IVs are associated with the exposure; (ii) Independence assumption—IVs are independent of unmeasured confounding; (iii) Exclusion restriction—IVs affect the outcome only through the exposure. However, these assumptions may be violated due to the pervasive horizontal pleiotropy [13, 14]. Under the plurality assumption (that the valid IVs form the largest group of IVs sharing the same causal parameter value), MR-cML is robust to the presence of some invalid IVs violating any or all of three IV assumptions and has been shown to perform better than many existing methods under various scenarios [15]. Furthermore, as shown before [16], with a simple IV screening procedure, MR-cML achieves good performance in inferring both causal directions and effect sizes between two traits while allowing bidirectional relationships (i.e. either trait is causal to the other at the same time). Thus, we will apply MR-cML in our causal graph framework, called **Graph-MRcML**.

One limitation of the original MR-cML is its implementation only for two-sample MR (i.e., assuming two independent GWAS summary datasets) [15]. However, in practice, multiple traits may come from the same study, as several lipid traits from the Global Lipids Genetics Consortium GWAS data to be used in our real data example [17]. More generally, as more international consortia and large-scale biobanks emerging, it is inevitable to have overlapping samples between some GWAS datasets. It has been shown that sample overlap may lead to biased estimates and inflated type-I errors in MR [18]. To address this, we first extend MR-cML to the overlapping-sample set-up, which turns out to be non-trivial, especially with respect to valid statistical inference. In addition, we establish theory that, perhaps surprisingly, the bias of the causal parameter estimator under the incorrect independence assumption (i.e. ignoring sample overlap) will disappear asymptotically (as the sample size increases); however, the usual (model-based) variance will be biased, thus we propose a robust/sandwich estimator. More importantly, the causal parameter estimator fully accounting for sample overlap is more efficient than the one under the working independence assumption. It is emphasized that, as a distinct feature, our proposed method not only estimates causal networks, but also can assess the statistical significance of any estimated causal effects. For this purpose, in addition to developing statistical theory for large-sample inference, we also develop a novel and effective data perturbation scheme for more accurate finite-sample inference by accounting for model fitting uncertainties (e.g. in selecting out invalid IVs). The latter task is technically challenging mainly because of the presence of some complex dependencies among the traits and the SNPs: we need to fully take into account of not only possible correlations among the traits (due to overlapping samples), but also each trait’s being used multiple times across many pairs of traits (thus inducing dependencies among the resulting estimates in a causal network) and linkage disequilibrium (LD) among the SNPs/IVs across all the traits (even if the SNPs/IVs are selected as independent for each trait). In particular, it would be impractical to restrict the SNPs/IVs to be independent across all the traits, leading to no or few SNPs.

There are several approaches in the MR literature aiming to estimate the direct causal effects among multiple traits. [19] proposed a two-step framework similar to ours, which used MR-Egger to construct a causal network of total effects, then under the sparsity assumption approximately invert it by penalized regression to infer the corresponding direct causal network. Besides the difference of our using more robust and efficient MR-cML versus their (modified) MR-Egger, no theory of their method is established; in particular, it is unclear how their proposed statistical inference would perform, partly due to technical challenges imposed by their using penalized regression. Another related method is two-step MR [20] or network MR [8], which focuses on the set-up with a candidate mediator between an exposure and an outcome. Our proposed method can be regarded as a generalization of this approach to infer a more complex causal network of multiple traits without pre-specifying causal directions and mediators. Finally, multivariable MR (MVMR) [9, 10] can be used to estimate direct effects of multiple exposures on an outcome. However, first, our method depends only on the validity of univariable MR (UVMR) (and the corresponding valid IV assumptions), while there are additional assumptions required for MVMR [21]. For example, a valid IV for UVMR may not be valid for MVMR, and there is a potential issue of multicollinearity in MVMR, leading to weak IV biases [22]. Second, existing MVMR methods all require the use of independent IVs for all exposures, sometimes leading to no or only few IVs for some exposures if the number of exposures is not too small. More generally, application of any existing MVMR method would reduce the number of the IVs, leading to loss of estimation efficiency and the possible issue of multicollinearity as to be confirmed in the real data example.

To summarize, we have two main contributions in methods development. First, we propose a general framework for inferring (including estimating and testing) both total and direct causal graphs among multiple traits of interest. Second, for better performance of the proposed framework, we extend the MR-cML method [15] to accommodate overlapping samples, and modify the network deconvolution algorithm, either of which can be useful in their own applications. Through extensive simulation studies, we show that the extended MR-cML performed better than the original one and other widely-used MR methods in the presence of sample overlap. We also show improved performance of our modified network deconvolution algorithm over that of the original one. Finally, we applied the proposed framework to 17 large-scale GWAS summary datasets (with median sample size of 256879 and median 48 IVs) to infer causal networks among 11 common cardiometabolic risk factors and 6 diseases, including 4 cardiometabolic diseases (coronary artery disease, stroke, type 2 diabetes, atrial fibrillation), Alzheimer’s disease (AD) (for its associations with some cardiometabolic risk factors/diseases [23]) and asthma (more as a negative control), identifying some interesting causal pathways.

## Materials and methods

### Causal model

**Fig 1:**
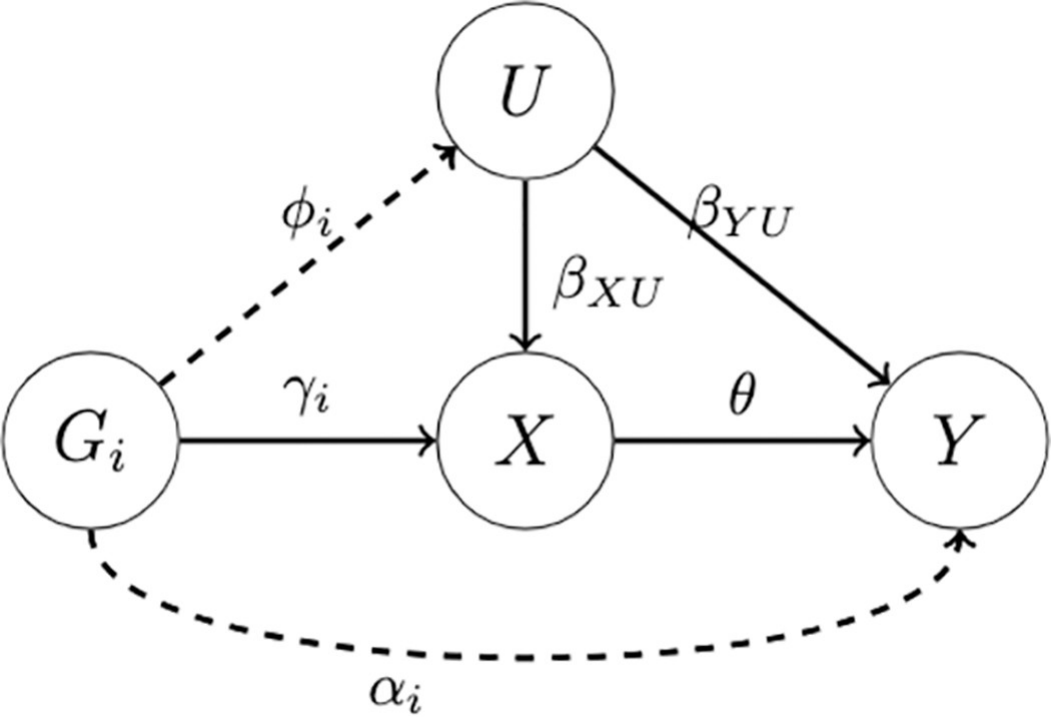
A causal model for the exposure *X* and the outcome *Y*.

Based on Fig 1, we have

$$\begin{array}{c} U=\phi_{i}G_{i}+\epsilon_{U},\;X=\gamma_{i}G_{i}+\beta_{XU}U+\epsilon_{X},\;Y=\theta X+\beta_{YU}U+\alpha_{i}G_{i}+\epsilon_{Y}, \end{array}$$

where *ϵ*_*U*_, *ϵ*_*X*_, *ϵ*_*Y*_ are independent random errors. We can further express the exposure *X* and the outcome *Y* as

$$\begin{array}{cc} X & =\left(\gamma_{i}+\beta_{XU}\phi_{i}\right)G_{i}+\left(\beta_{XU}\epsilon_{U}+\epsilon_{X}\right)\;≔\;b_{Xi}G_{i}+\epsilon_{X}^{*},\\ Y & =\left(\theta b_{Xi}+\beta_{YU}\phi_{i}+\alpha_{i}\right)G_{i}+\left(\theta \epsilon_{X}^{*}+\beta_{YU}\epsilon_{U}+\epsilon_{Y}\right)\;≔\;b_{Yi}G_{i}+\epsilon_{Y}^{*}. \end{array}$$

Accordingly, we have

$$b_{Xi}=\gamma_{i}+\beta_{XU}\phi_{i},$$ (1)

$$b_{Yi}=\theta b_{Xi}+\beta_{YU}\phi_{i}+\alpha_{i}\;≔\;\theta b_{Xi}+r_{i},$$ (2)

where *b*_*Xi*_ and *b*_*Yi*_ are the marginal effects of SNP *G*_*i*_ on the exposure *X* and the outcome *Y* respectively, *θ* is the causal effect of interest, and *r*_*i*_ represents all pleiotropic effects on *Y* (but not through *X*), including both uncorrelated pleiotropy (i.e. *α*_*i*_) and correlated pleiotropy (i.e. *β*_*YU*_*ϕ*_*i*_ that is correlated with *b*_*Xi*_). A SNP *i* is an invalid IV if *r*_*i*_ ≠ 0 (or *b*_*Xi*_ = 0).

Given the GWAS summary data $\left\{\left({\hat{\beta }}_{Xi},{\hat{\sigma }}_{Xi},{\hat{\beta }}_{Yi},{\hat{\sigma }}_{Yi}\right)\;:\;i=1,\ldots ,m\right\}$, where ${\hat{\beta }}_{Xi}$ and ${\hat{\beta }}_{Yi}$ are the estimates of *b*_*Xi*_ and *b*_*Yi*_ with the corresponding standard errors ${\hat{\sigma }}_{Xi}$ and ${\hat{\sigma }}_{Yi}$ respectively, the central goal of robust MR is to infer *θ* in the presence of invalid IVs.

### Extension of MR-cML to overlapping samples

In this section, we consider the situation when there are overlapping samples between two GWAS datasets, and the *m* IVs/SNPs are independent. It is reasonable to assume

$$(\begin{array}{c} {\hat{\beta }}_{Xi}\\ {\hat{\beta }}_{Yi} \end{array})\sim N((\begin{array}{c} b_{Xi}\\ \theta b_{Xi}+r_{i} \end{array}),(\begin{array}{cc} \sigma_{Xi}^{2} & \rho \sigma_{Xi}\sigma_{Yi}\\ \rho \sigma_{Xi}\sigma_{Yi} & \sigma_{Yi}^{2} \end{array})),$$

where *ρ* captures the correlation between ${\hat{\beta }}_{Xi}$ and ${\hat{\beta }}_{Yi}$ due to overlapping samples (and possibly other reasons) in the two GWAS datasets. We assume it is known or can be well-estimated; we will discuss its estimation later. We also assume $\sigma_{Xi}^{2}$ and $\sigma_{Yi}^{2}$ are known or well-estimated as ${\hat{\sigma }}_{Xi}^{2}$ and ${\hat{\sigma }}_{Yi}^{2}$ respectively. Then the log-likelihood (up to some constants) is

$$\begin{array}{cc} & l\left(\theta ,\left\{b_{Xi},r_{i}\right\};\left\{{\hat{\beta }}_{Xi},\sigma_{Xi},{\hat{\beta }}_{Yi},\sigma_{Yi}\right\},\rho \right)=\sum_{i=1}^{m}l_{i}\left(\theta ,b_{Xi},r_{i};{\hat{\beta }}_{Xi},\sigma_{Xi},{\hat{\beta }}_{Yi},\sigma_{Yi},\rho \right)=\\ & -\frac{1}{2(1-\rho^{2})}\sum_{i=1}^{m}(\frac{{\left({\hat{\beta }}_{Xi}-b_{Xi}\right)}^{2}}{\sigma_{Xi}^{2}}+\frac{{\left({\hat{\beta }}_{Yi}-\theta b_{Xi}-r_{i}\right)}^{2}}{\sigma_{Yi}^{2}}-2\rho \frac{\left({\hat{\beta }}_{Xi}-b_{Xi}\right)}{\sigma_{Xi}}\frac{\left({\hat{\beta }}_{Yi}-\theta b_{Xi}-r_{i}\right)}{\sigma_{Yi}}), \end{array}$$ (3)

where we use {*b*_*Xi*_, *r*_*i*_} = {(*b*_*Xi*_, *r*_*i*_) : *i* = 1, …, *m*} to represent a set of the parameters, and similarly for $\left\{{\hat{\beta }}_{Xi},\sigma_{Xi},{\hat{\beta }}_{Yi},\sigma_{Yi}\right\}$. With Eq (3), we obtain the constrained maximum likelihood estimator (cMLE) by solving

$$\begin{array}{c} {\text{min}}_{\theta ,b_{Xi},r_{i}}-l\left(\theta ,\left\{b_{Xi},r_{i}\right\};\left\{{\hat{\beta }}_{Xi},\sigma_{Xi},{\hat{\beta }}_{Yi},\sigma_{Yi}\right\},\rho \right)\;\text{subject}\;\text{to}\;\sum_{i=1}^{m}\;I\left(r_{i}\neq 0\right)=K, \end{array}$$ (4)

where *I*(⋅) is the indicator function, *K* is a tuning parameter representing the unknown number of invalid IVs. As MR-cML requires the plurality condition (see Assumption 2 in Section A.1 in S1 Text), there should be at least two valid IVs, i.e., *K* can be ranged from 0 to *m* − 2. We note that when *ρ* = 0, it becomes the method proposed by [15]. We refer this extended version of MR-cML to **MR-cML-C** (C for correlated samples), and the original version in [15] as **MR-cML-I** (I for independence) obtained under the (incorrect) working independence assumption of *ρ* = 0.

To infer a possibly bi-directional causal relationship between a pair of traits, we will apply bi-directional MR with an extra step as proposed in [16] to screen for valid IVs (see Section D.1 in S1 Text).

#### Estimation of causal parameter and its standard error with fixed *K*

Equating the first-order derivatives of log-likelihood Eq (3) to zero gives:

$${\hat{r}}_{i}\left(\theta ,b_{Xi}\right)=\left({\hat{\beta }}_{Yi}-\theta b_{Xi}\right)-\rho \left({\hat{\beta }}_{Xi}-b_{Xi}\right)\sigma_{Yi}/\sigma_{Xi},$$ (5a)

$${\hat{b}}_{Xi}\left(\theta ,r_{i}\right)=\frac{{\hat{\beta }}_{Xi}/\sigma_{Xi}^{2}-\rho \left({\hat{\beta }}_{Yi}-r_{i}+\theta {\hat{\beta }}_{Xi}\right)/\left(\sigma_{Xi}\sigma_{Yi}\right)+\theta ({\hat{\beta }}_{Yi}-r_{i})/\sigma_{Yi}^{2}}{1/\sigma_{Xi}^{2}-2\rho \theta /\left(\sigma_{Xi}\sigma_{Yi}\right)+\theta^{2}/\sigma_{Yi}^{2}},$$ (5b)

$$\hat{\theta }\left(\left\{b_{Xi},r_{i}\right\}\right)=\frac{\sum_{i=1}^{m}[b_{Xi}\left({\hat{\beta }}_{Yi}-r_{i}\right)/\sigma_{Yi}^{2}-\rho b_{Xi}\left({\hat{\beta }}_{Xi}-b_{Xi}\right)/\left(\sigma_{Xi}\sigma_{Yi}\right)]}{\sum_{i=1}^{m}b_{Xi}^{2}/\sigma_{Yi}^{2}}.$$ (5c)

For a given number of invalid IVs (0 < *K* ≤ *m* − 2), we use a coordinate descent-like algorithm to iteratively solve Eq (4). At the (*t* + 1)th iteration:

Step 1: Calculate $r_{i}^{\left(t+1\right)}=\left({\hat{\beta }}_{Yi}-\theta^{\left(t\right)}b_{Xi}^{\left(t\right)}\right)-\rho \left({\hat{\beta }}_{Xi}-b_{Xi}^{\left(t\right)}\right)\sigma_{Yi}/\sigma_{Xi}$; In order to select out *K* invalid IVs, we choose them as the ones with the largest
  $$\begin{array}{c} d_{i}^{\left(t+1\right)}\;≔\;l_{i}\left(\theta^{\left(t\right)},b_{Xi}^{\left(t\right)},r_{i}^{\left(t+1\right)};{\hat{\beta }}_{Xi},\sigma_{Xi},{\hat{\beta }}_{Yi},\sigma_{Yi},\rho \right)-l_{i}\left(\theta^{\left(t\right)},b_{Xi}^{\left(t\right)},0;{\hat{\beta }}_{Xi},\sigma_{Xi},{\hat{\beta }}_{Yi},\sigma_{Yi},\rho \right), \end{array}$$
  so that the log-likelihood is maximally increased. Specifically, we order $d_{i}^{\left(t+1\right)}$ decreasingly, then for *i* = 1, …, *K*, let $r_{\left(i\right)}^{\left(t+1\right)}={\hat{r}}_{\left(i\right)}\left(\theta^{\left(t\right)},b_{X(i)}^{\left(t\right)}\right)$ (Eq (5a)); for *j* = *K* + 1, …, *m*, let $r_{\left(i\right)}^{\left(t+1\right)}=0$.
Step 2: Update *b*_*Xi*_ and *θ* using Eqs (5b) and (5c):
  $$\begin{array}{c} b_{Xi}^{\left(t+1\right)}={\hat{b}}_{Xi}\left(\theta^{\left(t\right)},r_{i}^{\left(t+1\right)}\right),\;\theta^{\left(t+1\right)}=\hat{\theta }\left(\left\{b_{Xi}^{\left(t+1\right)},r_{i}^{\left(t+1\right)}\right\}\right). \end{array}$$

We repeat the above two steps until convergence, obtaining the final estimates $\hat{\theta }\left(K\right)$ and ${\left\{{\hat{r}}_{i}\left(K\right),{\hat{b}}_{Xi}\left(K\right)\right\}}_{i=1}^{m}$. It is noted that at the convergence the (estimated) invalid IVs (with ${\hat{r}}_{i}\neq 0$) do not contribute to estimating *θ*. We use the observed Fisher information to estimate the standard error (SE) of $\hat{\theta }\left(K\right)$.

#### Model selection and data perturbation

Following [15], we use BIC to select the set of invalid IVs. We denote *B*_0_ = {*i*|*r*_*i*_ ≠ 0, *i* = 1, …, *m*} the set of truly invalid IVs, with size |*B*_0_| = *K*_0_. Denote the cMLEs obtained from Eq (4) as $\hat{\theta }\left(K\right)$, ${\hat{b}}_{Xi}\left(K\right)$, and ${\hat{r}}_{i}\left(K\right)$ for *i* = 1, …, *m*, and ${\hat{B}}_{K}=\left\{i|{\hat{r}}_{i}\left(K\right)\neq 0,i=1,\ldots ,m\right\}$ the estimated set of invalid IVs. We estimate the *K* from a candidate set K based on the following Bayesian information criterion (BIC):

$$\begin{array}{c} \text{BIC}\left(K\right)=-2l\left(\hat{\theta }\left(K\right),\left\{{\hat{b}}_{Xi}\left(K\right),{\hat{r}}_{i}\left(K\right)\right\};\left\{{\hat{\beta }}_{Xi},\sigma_{Xi},{\hat{\beta }}_{Yi},\sigma_{Yi}\right\},\rho \right)+log\left(N\right)\cdot K, \end{array}$$ (6)

where *N* = min(*N*_1_, *N*_2_). We select $\hat{K}=\text{arg}\;{\text{min}}_{K\in K}\text{BIC}\left(K\right)$ and ${\hat{B}}_{\hat{K}}=\left\{i|{\hat{r}}_{i}\left(\hat{K}\right)\neq 0,i=1,\ldots ,m\right\}$. The final estimate $\hat{\theta }=\hat{\theta }\left(\hat{K}\right)$ and its estimated standard error $\hat{\text{SE}}\left(\hat{\theta }\left(\hat{K}\right)\right)$ are used to perform inference on *θ*. We refer this method to **MR-cML-BIC-C**.

The proposed MR-cML-BIC-C is based on the (consistently) selected set of valid IVs, ignoring inherent uncertainty in model selection, thus tending to underestimate standard errors for finite samples. To better account for model selection uncertainty, especially with a small (to medium) sample size, we adopt the data perturbation approach [15, 16], which is equivalent to bootstrapping the corresponding GWAS individual-level data [24]. Briefly, for the *b*−th perturbation, *b* = 1, …, *B*, we generate perturbed samples

$$\begin{array}{c} (\begin{array}{c} {\hat{\beta }}_{Xi}^{\left(b\right)}\\ {\hat{\beta }}_{Yi}^{\left(b\right)} \end{array})∼N((\begin{array}{c} {\hat{\beta }}_{Xi}\\ {\hat{\beta }}_{Yi} \end{array}),(\begin{array}{cc} \sigma_{Xi}^{2} & \rho \sigma_{Xi}\sigma_{Yi}\\ \rho \sigma_{Xi}\sigma_{Yi} & \sigma_{Yi}^{2} \end{array})), \end{array}$$

for *i* = 1, …, *m* independently. Then the remaining steps follow: we apply MR-cML-BIC-C on each perturbed dataset to obtain ${\hat{\theta }}^{\left(b\right)}$, and we use the sample mean and standard deviation over the *B* estimates, ${\hat{\theta }}^{\left(1\right)},\ldots ,{\hat{\theta }}^{\left(B\right)}$, from the *B* perturbed datasets as the final estimate and its standard error respectively. We call this method **MR-cML-DP-C**.

We use a generic notation **MR-cML-C**, referring either MR-cML-BIC-C or MR-cML-DP-C; we use similar notations for **MR-cML-I**. We also use MR-cML to denote either MR-cML-C or MR-cML-I.

#### Estimation of *ρ*

The correlation between the GWAS estimates of two (continuous) traits *X* and *Y* is given by $\rho =\frac{N_{0}}{\sqrt{N_{1}N_{2}}}r\left(\text{x},\text{y}\right)$, where *N*_0_ is the sample size of the overlapping samples and *r*(**x**, **y**) is the phenotypic correlation between the two traits, which can be estimated based on completely overlapped individual-level data (Eqs (4) and (5) in [25], Eq (7) in [26]). Without individual-level data, two commonly-used strategies to estimate *ρ* are (i) using the correlation between the two sets of GWAS null Z-scores [27]; and (ii) using the intercept from a fitted bivariate LD-score regression model (LDSC) [28]. We note that, while our motivation is to take into account of sample overlap between the two GWAS studies, other relevant sources for correlations can also be captured, including not only sample overlap but also population stratification and cryptic relatedness [29].

### Graph-MRcML

Now instead of considering two traits, suppose we have *T* traits/diseases, say *Y*_1_, …, *Y*_*T*_, and the goal is to construct a causal network among them. This can be done by first constructing an *total* causal graph (**G**_*tot*_), and second deconvoluting **G**_*tot*_ into the *direct* causal graph (**G**_*dir*_). Briefly, in the first step, we apply bi-directional MR-cML-C on *every* pair of traits and obtain the total causal graph **G**_*tot*_. However, such a graph may contain both direct and indirect causal effects, and we’d like to distinguish them to better understand the causal paths among the *T* traits. Therefore, in the second step, we use a network deconvolution method [12] to estimate the direct causal graph **G**_*dir*_.

We also extend a data perturbation scheme for statistical inference on such graphs. Briefly, we perturb the GWAS summary data for the *T* traits multiple times and obtain the estimated total and direct causal (effect) graphs with each perturbed dataset. Then the empirical distribution of such estimates from multiple perturbed samples is used for inference. To account for multiple testing, we use the Bonferroni adjustment with the effective number of independent tests estimated as in [30] (see Section D.2 in S1 Text).

#### Using MR-cML for estimation and inference of G_*tot*_

Let $\hat{\text{B}}$ and **S** denote the *m* × *T* matrices of GWAS summary statistics for the *T* traits: each entry ${\hat{\text{B}}}_{i,j}$ and **S**_*i*,*j*_ are the estimated association effect size and standard error between the *i*-th SNP and the trait *Y*_*j*_ respectively. Note that some ${\hat{\text{B}}}_{i,j}$ (and **S**_*i*,*j*_) can be missing if the *i*-th SNP is never used in the analyses involving the *j*-th trait. We use ${\hat{\text{B}}}_{i,\cdot }$ and ${\hat{\text{B}}}_{\cdot ,j}$ to denote the *i*-th row and the *j*-th column of matrix $\hat{\text{B}}$ respectively. Let P denote the *T* × *T* correlation matrix, i.e. **P**_*j*,*j*_ = 1 and **P**_*j*,*k*_ = **P**_*k*,*j*_ = *ρ*_*jk*_ for *j* ≠ *k*, where *ρ*_*jk*_ is the correlation between the GWAS estimates for traits *j* and *k*.

The algorithm consists of two parts. In the first part we perform data perturbation on the GWAS data, and in the second part we estimate the total causal (effect) network **G**_*tot*_ with the perturbed data. Instead of using the data perturbation scheme mentioned in Model selection and data perturbation for each pair separately, here we will perturb the summary statistics for *all* traits together (i.e. the whole matrix $\hat{\text{B}}$). The reason is that, there might be correlations among the SNPs/IVs (i.e. the rows of $\hat{\text{B}}$) besides the correlations among the GWAS traits (i.e. the columns of $\hat{\text{B}}$), and we need to take the correlations into account. For example, let’s say we have GWAS summary data for three traits, HDL, LDL and TG, which may come from the same consortium (i.e. with overlapping samples). Then *between* the set of IVs for HDL→LDL and the set of IVs for LDL→TG, there might be SNPs in linkage disequilibrium (LD), though *within* the two sets, IVs were independent as selected for MR in practice. For this purpose, we use a matrix normal distribution [31] to model and perturb the data. Let $\text{Z}=\hat{\text{B}}/\text{S}$ denote the matrix of Z-scores, then for the *b*-th perturbed dataset, ${\text{Z}}^{\left(b\right)}=\text{Z}+{\text{E}}^{\left(b\right)},{\hat{\text{B}}}^{\left(b\right)}={\text{Z}}^{\left(b\right)}*\text{S}$, where * is the element-wise multiplication and **E**^(*b*)^ follows a matrix normal distribution:

$$\begin{array}{c} {\text{E}}^{\left(b\right)}∼MN_{m,T}\left(0_{m\times T},\text{R},\text{P}\right), \end{array}$$

where **R** is the LD matrix of the *m* SNPs. Or equivalently, we have $\text{vec}\left({\text{E}}^{\left(b\right)}\right)∼N\left(0_{mT},\text{P}⊗\text{R}\right),$ where ⊗ denotes the Kronecker product and vec(**E**^(*b*)^) denotes the vectorization of **E**^(*b*)^. To generate **E**^(*b*)^, we first generate an *mT*-vector **v** from a standard normal distribution, and vec(**E**^(*b*)^) = **Av**, where **AA**^*T*^ = **P** ⊗ **R** and the matrix decomposition is done with eigen decomposition. Then we convert vec(**E**^(*b*)^) back to **E**^(*b*)^ and obtain the perturbed GWAS data ${\hat{\text{B}}}^{\left(b\right)}=\hat{\text{B}}+\text{S}*{\text{E}}^{\left(b\right)}$. In practice, we use the 1703 approximately independent LD blocks [32], and extract the LD matrix **R** using the 1000 Genomes Phase 3 EUR population as the reference panel in TwoSampleMR [33]. The algorithm is summarized as follows:

Step 1: Generate the perturbed data ${\hat{\text{B}}}^{\left(b\right)}$ as described above.
Step 2: Apply bidirectional MR-cML-BIC-C on every pair of traits to obtain (all the non-diagonal entries in) ${\text{G}}_{tot}^{\left(b\right)}$.

We repeat the above steps *B* times and use the element-wise mean of ${\left\{{\text{G}}_{tot}^{\left(b\right)}\right\}}_{b=1}^{B}$ as the final estimate for **G**_*tot*_, i.e., ${\hat{\text{G}}}_{tot}=\sum_{b=1}^{B}{\text{G}}_{tot}^{\left(b\right)}/B$. We also use the element-wise standard deviation of ${\left\{{\text{G}}_{tot}^{\left(b\right)}\right\}}_{b=1}^{B}$ to estimate the standard error for each entry in ${\hat{\text{G}}}_{tot}$. Lastly, the p-value for each (off-diagonal) entry of ${\hat{\text{G}}}_{tot}$ (or the edge in the corresponding network) (to test for the entry being 0 or the edge is absent) is calculated based on the standard normal distribution.

#### Using network deconvolution for estimation and inference of G_*dir*_

Under the assumption that the causal relationships between variables are linear, there is a relationship between **G**_*tot*_ and **G**_*dir*_ [12]:

$${\text{G}}_{tot}={\text{G}}_{dir}+{\text{G}}_{dir}^{2}+{\text{G}}_{dir}^{3}+\cdots ={\text{G}}_{dir}\left(\text{I}+{\text{G}}_{dir}+{\text{G}}_{dir}^{2}+{\text{G}}_{dir}^{3}+\ldots \right)={\text{G}}_{dir}{\left(\text{I}-{\text{G}}_{dir}\right)}^{-1},$$ (7)

where **I** is the identity matrix. The first equality in Eq (7) is by definition with an intuitive interpretation: a total effect represented by an element (i.e. edge) in **G**_*tot*_ can be decomposed into a direct effect represented by the corresponding edge in **G**_*dir*_ and the sum of all indirect effects mediated through one, two, ⋯, up to (infinitely) many nodes (due to possible cycles) as represented by the corresponding edges in ${\text{G}}_{dir}^{2},{\text{G}}_{dir}^{3},\cdots$ [34]. An illustrative example is given in Section D.3 in S1 Text. If and only if the spectral radius (i.e. the largest absolute value of all real/complex eigenvalues) of **G**_*dir*_ is less than 1, the third equality in Eq (7) holds [34, 35]. Then it is easy to show

$${\text{G}}_{dir}={\text{G}}_{tot}{\left(\text{I}+{\text{G}}_{tot}\right)}^{-1}.$$ (8)

Note that in **G**_*tot*_, only the off-diagonal elements are estimated by bidirectional MR. For the diagonal elements, we may follow the practice in [12] of setting them to zeros, and we refer this approach to **Graph-MRcML-d0**. This is correct if there is no cycle in the underlying direct graph. However, with cycles in **G**_*dir*_, in general the corresponding diagonal elements in **G**_*tot*_ are not zeros. To see this, we rewrite Eq (7) as

$$\begin{array}{c} {\text{G}}_{tot}={\text{G}}_{dir}+{\text{G}}_{dir}\left({\text{G}}_{dir}+{\text{G}}_{dir}^{2}+{\text{G}}_{dir}^{3}+\ldots \right)={\text{G}}_{dir}+{\text{G}}_{dir}{\text{G}}_{tot}={\text{G}}_{dir}\left(\text{I}+{\text{G}}_{tot}\right). \end{array}$$

Denote the adjacency matrices **G**_*tot*_ = (*T*_*ij*_) and **G**_*dir*_ = (*D*_*ij*_), then the *i*-th diagonal element of **G**_*tot*_ can be expressed as

$$\begin{array}{c} T_{ii}=\sum_{j}\;D_{ij}\left(I\left(j=i\right)+T_{ji}\right)=\sum_{j\neq i}\;D_{ij}T_{ji}, \end{array}$$ (9)

where the second equality follows from the assumption that there is no self-loop in the direct graph (i.e. *D*_*ii*_ = 0). Accordingly, we propose a heuristic approach to specify the diagonal elements of **G**_*tot*_. We obtain the initial estimate of **G**_*dir*_ by setting ${\hat{T}}_{ii}=\sum_{j\neq i}T_{ij}T_{ji}$. Then we update ${\hat{T}}_{ii}$ and ${\hat{\text{G}}}_{dir}$ iteratively based on Eqs (9) and (8) till convergence. If it fails to converge, we will use the initial estimate (by setting ${\hat{T}}_{ii}=\sum_{j\neq i}T_{ij}T_{ji}$, which can serve as a good approximation in some scenarios, e.g. when *T*_*ij*_ is close to *D*_*ij*_). We refer this approach to **Graph-MRcML-d1**. As in the previous section, we leverage data perturbation to obtain a final estimate and perform statistical inference of the direct causal graph. More discussions and illustrative examples are given in Section D.4 in S1 Text.

There are some criticisms of the original network deconvolution paper [12]; see https://liorpachter.wordpress.com/2014/02/11/the-network-nonsense-of-manolis-kellis/. First of all, the key idea of network deconvolution is in Eq (7), based on a well-established and widely-used definition of the total effects in terms of the direct effects in the literature of linear structural equation modeling for directed causal graphs [34]. Second, we only consider smaller directed graphs with the total effects estimated by UVMR with much larger sample sizes [9, 10]. Hence the concern on relatively poor performance of network deconvolution compared to Gaussian graphical modeling (i.e. using the correlation and partial correlation to estimate the total and direct effects in the undirected graphs respectively for high-dimensional data) is not relevant here. Third, due to the differences between our and the original implementations, other main criticisms are not applicable here. Specifically, we do not scale, threshold or symmetrize the total effect graph **G**_*tot*_, hence there are no corresponding parameters (and their tuning). Instead of applying eigen-decomposition to the total graph, we invert the matrix in Eq (8) directly. We do acknowledge that the method requires the assumption that the spectral radius of the direct causal graph is less than 1, which may be violated in practice. However, as to be shown in the numerical examples, our method performed well (without encountering the spectral radius issue).

### Theory

As shown in Section A in S1 Text, our proposed methods enjoy some desirable statistical properties: under mild conditions, the BIC can consistently select valid IVs; both the MR-cML-BIC-I and MR-cML-BIC-C estimators are consistent and asymptotically normal for the true causal parameter *θ*; and the Graph-MRcML estimators are consistent and asymptotically normal for the true total and direct causal effect graphs.

### Simulation for MR with sample overlap

In this simulation, we investigated the performance of different MR methods in the presence of overlapping samples. We simulated data as follows:

$$\begin{array}{cc} & \text{U}=\text{G}\phi +\epsilon_{U},\;\text{X}=\gamma \cdot \text{G}+\text{U}+\epsilon_{X},\;\text{Y}=\theta \cdot \text{X}+\text{G}\alpha +\text{U}+\epsilon_{Y}, \end{array}$$

where ***ϵ***_*U*_, ***ϵ***_*X*_, ***ϵ***_*Y*_ were generated from N(0,1) independently. 20 IVs were generated independently from a binomial distribution with minor allele frequency (MAF) 0.3 and the IV strength *γ* was set to 0.08 for all IVs. We considered 0% and 30% invalid IVs. In the case of 0% invalid IVs, the set-up was the same as that in [18]. In the case of 30% invalid IVs, we generated the direct effect ***α*** iid from $N(0.04,0.05^{2})$, and considered (i) uncorrelated pleiotropy (i.e., ***ϕ*** = 0) and (ii) correlated pleiotropy, where ***ϕ*** was generated iid from Unif(0,0.01). The causal effect *θ* was set to be 0 or 0.2. Additionally, we generated 1000 null SNPs with the same MAF, of which we used the sample correlation of the Z-scores to estimate *ρ*. The GWAS sample sizes for the exposure and the outcome were set as *N*_1_ = *N*_2_ = *N* = 25000. We varied the proportion of sample overlap as one of {0%, 50%, 80%, 100%}. For example, the exposure GWAS summary statistics were calculated using the first 25 000 individuals, and the outcome GWAS summary statistics were calculated using the next 25 001–50 000 individuals for 0% overlap, or using individuals 12 501–37 500 for 50% overlap.

For each simulation set-up, we ran 1000 replicates and compared some popular MR methods, MR-IVW [36], weighted-median [37], weighted-mode [38], MR-Mix [39], MR-RAPS [40], MR-cML-I [15], with the proposed MR-cML-C.

### Simulation for direct causal network inference

**Fig 2:**
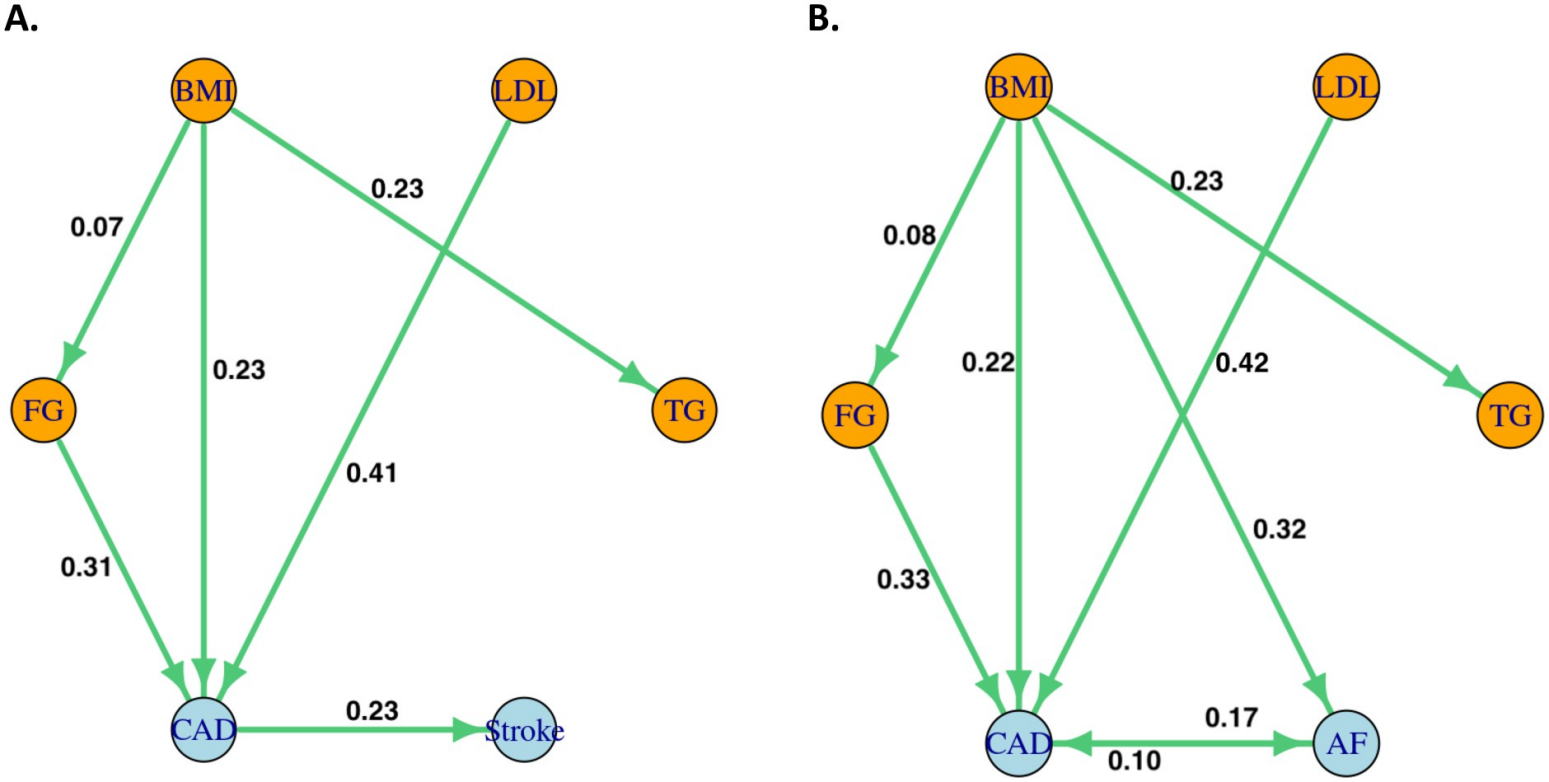
Estimated direct causal graphs for 6 traits.

We conducted simulations based on real GWAS summary statistics to study the performance of Graph-MRcML. We generated GWAS summary statistics based on the direct graph among 6 traits as shown in Fig 2. We considered scenarios with and without cycles in the direct graph, corresponding to Fig 2A and 2B respectively. Briefly, we first generated the true GWAS effect sizes **B** based on the underlying direct graph, and then generated the GWAS estimates $\hat{\text{B}}$ based on the matrix normal distribution introduced before to capture the LD structure and sample structure among GWAS data as in the real data analysis. We also considered different GWAS sample sizes by varying the standard error matrix **S**. Details are given in Section D.5 in S1 Text.

We repeated the simulation 100 times for each set-up, and applied Graph-MRcML with 200 data perturbations on each simulated dataset $\hat{\text{B}}$ and **S**, with other inputs remaining the same as in the real data analysis, including the LD matrix **R** and the correlation matrix **P** of the GWAS summary data, and the set of IVs used in each MR analysis after the screening process. We also performed a simulation to study the influence of the number of IVs used in an analysis.

### Real data analysis

We applied the proposed Graph-MRcML framework to study the causal relationships among 17 traits, including 11 cardiometabolic risk factors and 6 diseases. The 11 risk factors were triglycerides (TG), low-density lipoprotein cholesterol (LDL), high-density lipoprotein cholesterol (HDL), Height, body-mass index (BMI), birth weight (BW), diastolic blood pressure (DBP), systolic blood pressure (SBP), fasting glucose (FG), Smoke (cigarette per day) and Alcohol (alcoholic drinks per week). The 6 diseases were coronary artery disease (CAD), stroke, type 2 diabetes (T2D), asthma (more as a negative control), atrial fibrillation (AF) and Alzheimer’s disease (AD). The sample sizes for the 17 GWAS datasets ranged from 10 083 to 1 030 836, with a median of 256 879. We followed the same data pre-process steps described in Section 2.3 of [16] to prepare the data using TwoSampleMR package [33].

## Results

### Simulation for MR with sample overlap: Better type-I error control and higher power of MR-cML-C than other methods

We compared the data perturbation version of MR-cML-I and MR-cML-C, and other commonly used MR methods in this section. Here we discuss main findings while more results are given in the Section B in S1 Text.

**Fig 3:**
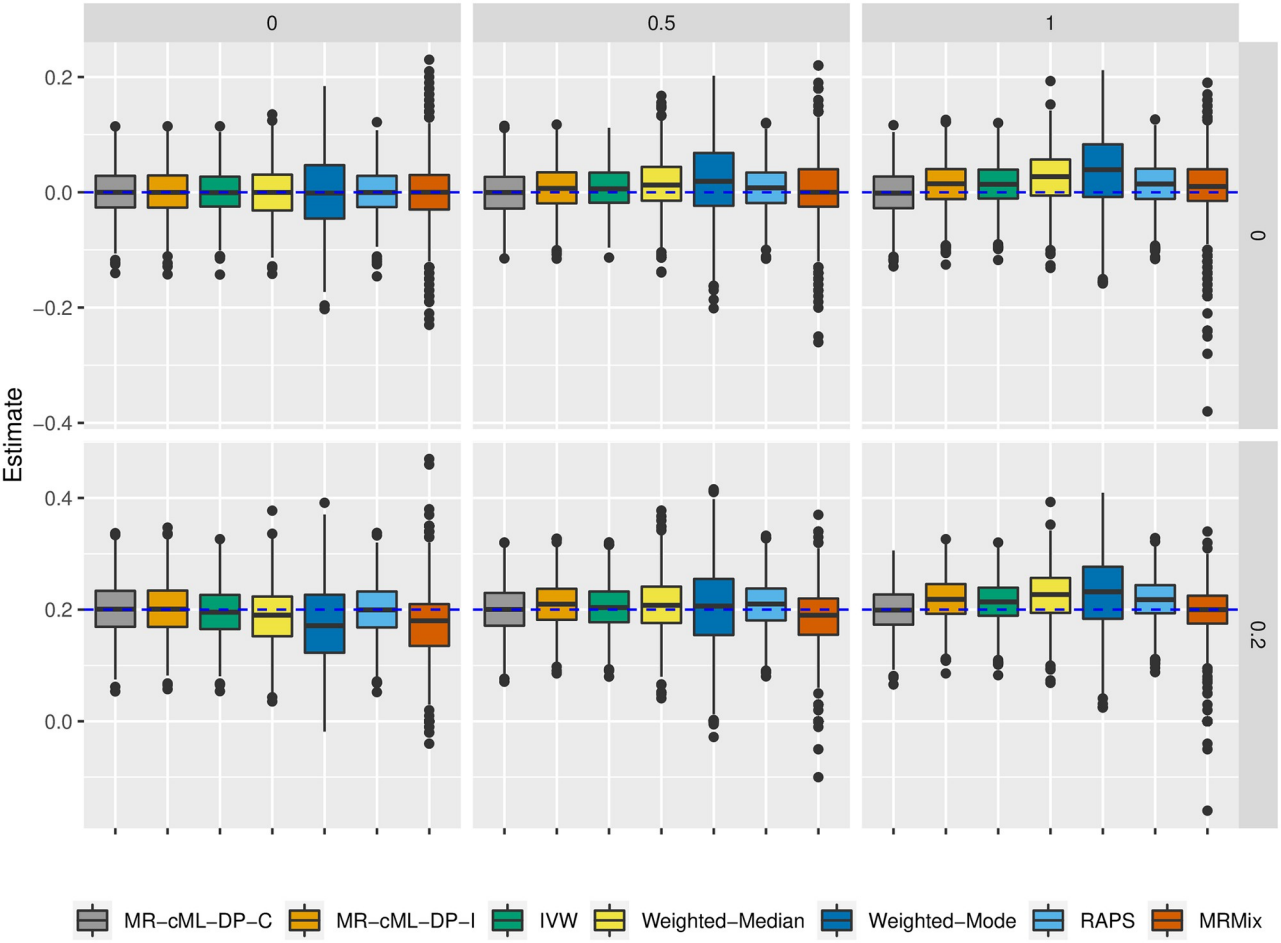
Estimates of the causal effect *θ* with 0% invalid IVs across 1000 replicates. From left to right correspond to 0%, 50% and 100% overlapping samples. Top panel: *θ* = 0 and bottom panel: *θ* = 0.2.

In the case of no invalid IVs, most of the methods were able to control the type-I error reasonably well in the presence of sample overlap; only MR-IVW and MR-RAPS had slightly inflated type-I errors. MR-cML-DP-C, MR-cML-DP-I, IVW, Weighted-Median and MR-RAPS had comparably higher power than Weighted-Mode and MR-Mix (Fig H in S1 Text). However, as shown in Fig 3, only MR-cML-DP-C gave unbiased causal estimates across all the scenarios, while other methods might remain biased, even more so in some situations, as the proportion of sample overlap increased.

**Fig 4:**
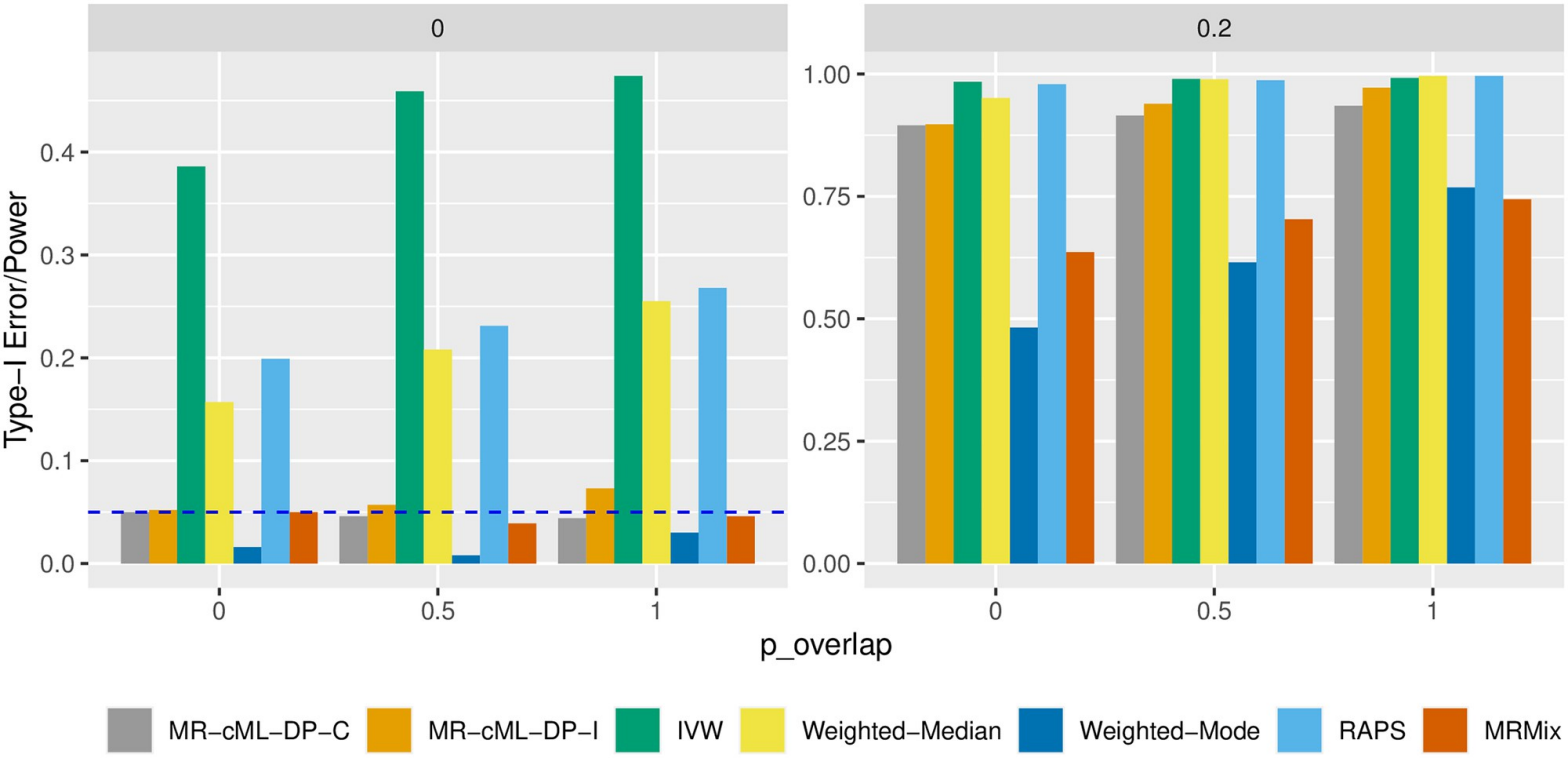
Empirical type-I error and power in the presence of 30% invalid IVs with correlated pleiotropy. X-axis represents different proportions of sample overlap (0%, 50% and 100%). Left: *θ* = 0 (type-I error) and right: *θ* = 0.2 (power).

In the case of 30% invalid IVs with correlated pleiotropy, Fig 4 shows the empirical type-I error (left) and power (right) for different methods. Only MR-cML-DP-C, Weighted-Mode and MR-Mix could control the type-I error as the proportion of sample overlap increased, and MR-cML-DP-C had the highest power among these three methods. As shown in Section B in S1 Text, MR-cML-DP-C yielded smaller biases and MSEs than many other methods, especially when the proportion of sample overlap was high. In particular, with the working independence assumption, MR-cML-DP-I was able to control the type-I error when there was no sample overlap, but had slightly inflated type-I errors as the sample overlapping proportion increased. In addition, as shown in S1 Text, while MR-cML-C and MR-cML-I yielded biased estimates because both methods sometimes failed to identify all invalid IVs (perhaps due to the small sample size and/or small effects of some invalid IVs), the bias of MR-cML-C was smaller than that of MR-cML-I in presence of sample overlap. A likely reason was that MR-cML-C performed better in identifying invalid IVs than MR-cML-I (Fig O in S1 Text). It is also notable that as the proportion of the overlapping samples increased, even as the total sample size decreased, the estimates of the causal parameter *θ* became more precise with smaller variances, and the estimate by MR-cML-C was less biased (Figs I and L in S1 Text). This suggests an advantage of using the overlapping-sample or only one-sample design over using the two-sample design in MR for causal inference (when the sample structure is correctly accounted for).

#### Other simulation results

We performed simulations to study the consistency of MR-cML-BIC-I when we mis-specified the model by ignoring the correlation between the two GWAS summary datasets. First, BIC based on the incorrectly specified model was able to select the correct set of invalid IVs with increasing probabilities as the sample size being increasing. Second, we studied the performance of MR-cML-BIC-I when assuming it correctly selected the set of invalid IVs. In this case, the bias of MR-cML-BIC-I was going to zero as the sample size increased, but the standard error was overestimated using the usual (naive or model-based) variance estimator. On the other hand, the robust sandwich variance estimator was consistent in estimating the standard error but the confidence interval based on it had a low coverage rate mainly due to the finite-sample bias of the causal estimate by MR-cML-BIC-I. We also point out that, although the selection consistency of BIC used in MR-cML-BIC-I and the estimation consistency of MR-cML-BIC-I still hold in the presence of sample overlap, MR-cML-BIC-C still outperformed MR-cML-BIC-I, especially when the sample size was not large enough. Details are given in S1 Text.

A model averaging (MA) approach was proposed in [15] (that is often combined with data perturbation) to achieve better inferential performance for finite samples. We also adopted the MA approach in MR-cML-C, called MR-cML-MA-C (and MR-cML-MA-DP-C with data perturbation). We found that MR-cML-MA-C performed better than MR-cML-BIC-C, but it was still unsatisfactory: it might yield inflated type-I errors. However, with data perturbation, there was no additional benefit from model averaging; MR-cML-MA-DP-C performed similarly to MR-cML-DP-C. For this reason, we skip the discussion of MA in the Methods section, and we no longer recommend the use of MA. The detailed results are given in the Section C.1 in S1 Text.

### Simulation for direct causal network inference: Recovery of the direct causal network by Graph-MRcML

In this section, we studied the performance of Graph-MRcML in recovering the direct causal network. We summarize the main findings here while more detailed results and discussions are provided in S1 Text.

We first applied Graph-MRcML on the simulated GWAS summary data. The iterative algorithm in Graph-MRcML-d1 converged successfully in all simulations for both set-ups. As shown in Table 1, Graph-MRcML-d1 was able to control the type-I error reasonably well and yielded high power. Graph-MRcML-d0 (i.e., the diagonal elements of the total graph were set to zero) also had similar type-I error and power (Table N in S1 Text). Both methods gave only small biases for some entries in the direct graph as shown in Tables O and P in S1 Text.

**Table 1:** Empirical type-I error and power by Graph-MRcML-d1 for (a) Set-up (a) and (b) Set-up (b). Numbers underlined correspond to power.

	BMI	LDL	FG	TG	CAD	Stroke
BMI		0	0.59	1	0.9	0

LDL	0		0	0.01	1	0

FG	0	0		0	0.89	0

TG	0	0.01	0		0.06	0.01

CAD	0	0	0	0		1

Stroke	0	0.01	0	0	0	

**(a)** Set-up (a)						
	BMI	LDL	FG	TG	CAD	AF
BMI		0	0.8	1	0.87	1

LDL	0.02		0	0	1	0

FG	0	0		0	0.96	0

TG	0	0	0		0.01	0

CAD	0	0	0	0		1

AF	0	0	0	0	1	

**(b)** Set-up (b)						

We also applied Graph-MRcML to the simulated GWAS summary data of various sample sizes. Detailed results are given in Section D.5.2 in S1 Text. To summarize, there were slightly inflated type-I errors with a smaller sample size, probably because of MR-cML-BIC-C failing to identify all invalid IVs. But as the sample size increased, type-I error was well controlled. Furthermore, as there was no cycle in Set-up (a), the diagonal elements of the total graph were all zeros. As a result, the diagonal elements of the total graph in both Graph-MRcML-d0 and Graph-MRcML-d1 were consistent, and the resulting direct graph estimates approached the true values as the sample size increased (Tables Q to S in S1 Text). On the other hand, in the presence of cycles, the true diagonal elements of the total graph in Set-up (b) were not all zeros, hence the direct graph estimate by Graph-MRcML-d0 might be off. In contrast, if it converged successfully, Graph-MRcML-d1 yielded almost unbiased estimates of the direct graph as the sample size increased (Tables W to Y in S1 Text). In general, based on our experience, in the absence of cycles in a direct graph, Graph-MRcML-d0 and Graph-MRcML-d1 performed similarly; otherwise, Graph-MRcML-d1 would have better performance than Graph-MRcML-d0.

As shown in the real data analysis to be discussed next, the number of IVs used in each MR analysis had a wide range. To study the impact of the number of IVs on the performance of the proposed method, we conducted an additional simulation with more IVs for FG, which had the least IVs in the real data analysis. We found that when we had more (valid) IVs for FG, the precision of the direct effect estimates of FG on the other traits increased, while the precision for other direct effect estimates might or might not change much, depending on the underlying relationship among the traits. Details are given in Section D.5.3 in S1 Text.

### Real data analysis

We applied the proposed Graph-MRcML framework to study the causal relationships among 17 traits, including 11 cardiometabolic risk factors and 6 diseases. We first estimated the correlation matrix **P** using both the null Z-scores approach and bivariate LDSC regression as discussed in Estimation of *ρ*. As shown in Fig R in S1 Text, most of the GWAS traits were not highly correlated with each other except for a few such as {SBP, DBP}, {TG, HDL, LDL}, the GWAS datasets of which were collected from the same study respectively. Two approaches generally gave very similar results with only slight differences, and we used the LDSC estimates in the subsequent analysis. For the pairs with small correlations (|*ρ*| < 0.1), we set them to be 0 in the subsequent analysis because, as shown by our numerical and theoretical studies, the results would be robust to ignoring such small correlations.

The numbers of IVs used in all pairwise bi-directional MR analyses (after the IV screening procedure) ranged from 6 to 374, with a median of 48. We performed *B* = 2000 data perturbations and applied bidirectional MR-cML-BIC-C on each perturbed dataset to obtain the total graph. Due to the temporal order of birth weight and the other traits, the total causal effect of each trait to birth weight was set to zero. Given the possible presence of cycles in the underlying (unknown) direct graph (e.g. between SBP and DBP), we applied Graph-MRcML-d1 to obtain the direct graph. We will discuss the results by Graph-MRcML-d1 based on the Bonferroni-adjusted significance level with 228 effective tests, i.e. 0.05/228 ≈ 2.2e-4. Results by Graph-MRcML-d0 are discussed in Section E.2 in S1 Text, where we might end up with an estimated direct network with a spectral radius greater than one.

Additional investigations on the relationship among lipid traits and glycemic traits [41] are discussed in Section E.4 in S1 Text. Our findings are similar to those in [41], suggesting that fasting insulin has plausible direct effects on TG and HDL.

#### Total causal effect network identifies many causal relationships among risk factors and complex diseases

**Fig 5:**
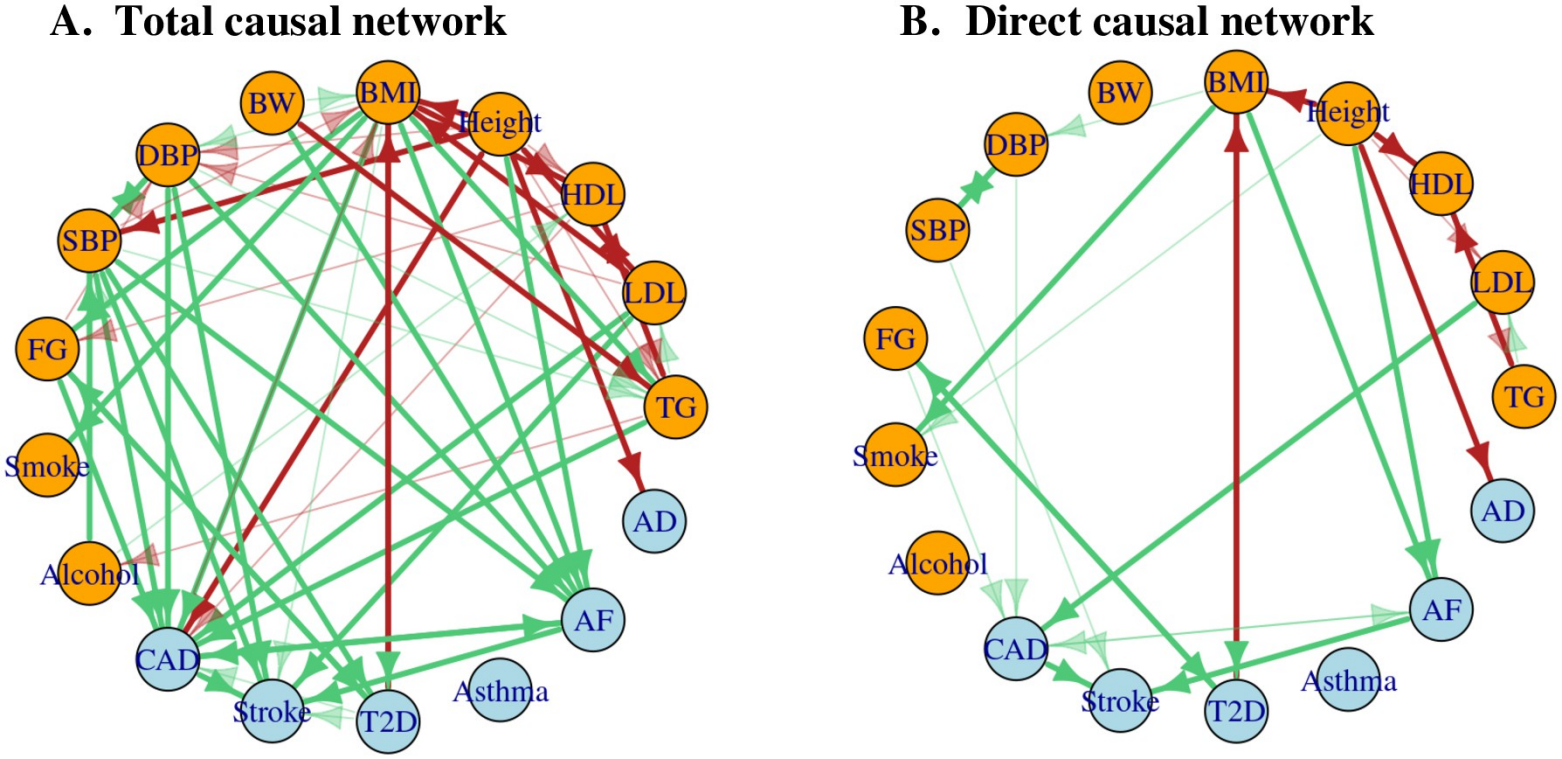
The estimated total (A) and direct (B) causal graphs for the 11 risk factors and 6 diseases. The edges in green represent positive effects and those in red are negative ones. The nodes in blue are diseases and those in orange are risk factors. The dark-solid edges are identified at the Bonferroni-adjusted significance level, while the light-colored ones are marginally significant at a less stringent level of 6.5e-3.

Fig 5A shows the inferred total causal graph (${\hat{\text{G}}}_{tot})$ for the 17 traits. In a total causal graph, an edge *A* → *B* represents the total causal effect of *A* on *B*; its presence or effect size does not depend on whether or what other variables are included in the graph. In other words, all or a part of a total causal effect may be the sum of all mediating effects through other variables included or not included in the graph. First, it suggested that BMI had a positive causal effect on CAD. It also identified many well-accepted causal relationships from the risk factors to diseases as discussed in [42], such as DBP → CAD, LDL → CAD, FG → T2D and so on. As a negative control, no causal path towards asthma was identified. It is also noted that a negative causal effect between Height and AD was suggested. Many observational studies have found that height is inversely associated with the risk of AD [43–45], and a recent longitudinal study that analyzed data from hundreds of thousands of men also found a link between height and the likelihood of developing dementia [46].

Second, some bidirectional relationships between a risk factor and a disease were identified: BMI ↔ T2D, FG ↔ T2D. BMI and FG are both well-accepted causal risk factors for T2D. For direction FG ← T2D, it is possible that the pancreas makes more insulin to make up for insulin resistance in T2D, and blood sugar levels build up overtime. For BMI ← T2D, T2D may cause weight loss since the cells cannot get the energy they need from glucose, and the body breaks down fat to use for energy instead, but more studies are needed.

Third, there were many interesting links between the risk factors. For example, our method inferred a positive causal link BMI → Smoke (cigarette per day). Previous observational studies have reported a positive correlation between BMI and smoking intensity [47], and a common biological basis for nicotine addiction and obesity was also suggested with genetic evidence [48]. Recently, an MR study suggested that obese individuals are more likely to smoke and with a higher smoking intensity in both the discovery and replication samples [49].

Lastly, some links among the diseases were identified, such as CAD ↔ AF, AF → Stroke and CAD → Stroke. Common heart disorders are risk factors for stroke [50], for example, CAD increases the risk for stroke, because plaque builds up in the arteries and blocks the flow of oxygen-rich blood to the brain. Also AF can cause blood clots that may break loose and travel to another part of the body, cutting off blood supply to the brain [51]. Studies have shown that there is a vicious cycle between CAD and AF [52].

#### Direct causal effect network suggests direct causal pathways

Fig 5B shows the inferred direct causal graph (${\hat{\text{G}}}_{dir})$ for the 17 traits. It is notable that this ${\hat{\text{G}}}_{dir}$ and all the direct graph estimates from the 2000 perturbed datasets had a spectral radius smaller than one, while the iterative algorithm in Graph-MRcML-d1 converged successfully in all cases. In a direct causal graph, an edge *A* → *B* represents the direct causal effect of *A* to *B* after conditioning on all the other variables included in the graph; in other words, it is the remaining causal effect of *A* to *B* after accounting for (i.e. removing) all possible mediating effects of *A* to *B* through any of the other variables included in the graph. Accordingly, the direct effect size of *A* to *B* also depends on what other variables are included in the graph.

First, we can see that several independent risk factors of diseases were identified after accounting for other traits in the graph. For example, LDL was an independent risk factor for CAD. In the Cooper Clinic Longitudinal Study [53], LDL was found to be associated with the CAD mortality in a multivariable model after adjusting for atherosclerotic CVD risk factors such as HDL, current tobacco use, hypertension, BMI and glucose. Another example is BMI → AF. Many observational cohort studies have highlighted BMI as an independent risk factor for AF after adjusting for traditional risk factors. In the Women’s Health study, BMI was found to be associated with elevations in AF risk after accounting for a wide range of covariates including diabetes, hypertension, history of hypercholesterolemia, alcohol consumption and smoking [54]. And in the Danish Diet, Cancer, and Health Study [55], BMI was found to be significantly associated with AF after adjusting for height, smoking, alcohol consumption, hypertension, diabetes, heart diseases, etc. Similarly, height is also a well-known independent risk factor for AF (as shown by Height → AF) after adjusting for many traditional risk factors as evidenced by many studies [56–58]. Overall, some of our findings here have lent strong support for some existing causal hypotheses drawn from previous observational cohort studies.

Perhaps as expected, the direct causal graph was less dense than the total causal graph in Fig 5A. First, multiple edges among the risk factors in the total causal graph were removed from the direct causal graph. For example, the edge from BMI to FG disappeared in the direct graph, which was probably because T2D served as an important mediator: BMI → T2D → FG. At the same time, the reverse path FG → T2D → BMI was also significant, suggesting the mechanisms underlying obesity, T2D and fasting glucose might be complicated, and more studies are needed [59].

Moreover, some edges between the risk factors and diseases were also removed. For example, LDL → Stroke is a known causal pair, however, as shown in Fig 5B, CAD might act as a mediator: LDL → CAD → Stroke. Another important question of interest is the role of BMI on CAD. While the total graph suggested BMI as a causal risk factor for CAD, it has been controversial about whether BMI is an independent risk factor for CAD [2]. Obesity is associated with many pathophysiological mechanisms involved in the development of CAD, such as preposition to insulin resistance and type 2 diabetes mellitus, lipid abnormalities and hypertension. In our analysis, the direct effect of BMI on CAD was not significant (p-value ≈ 0.16) after accounting for other factors; the direct causal effect estimate of BMI on CAD was attenuated from an estimated total causal effect of 0.31 (in the logOR scale) in ${\hat{\text{G}}}_{tot}$ to 0.09 in ${\hat{\text{G}}}_{dir}$. Furthermore, at a less stringent significance level (p-value<6.5e-3), there were indirect causal pathways from BMI to CAD via blood pressures, T2D and fasting glucose. Overall, our results suggested that BMI may be considered as a ‘minor’ independent risk factor for CAD after accounting for other factors or comorbidities [60].

For comparison, we also applied MVMR-IVW and MVMR-Robust [61] to investigate the causal effects of the 15 traits on CAD; we did not include Alzheimer’s disease (mainly because we did not expect to detect its causal effect on CAD). We used the mv_extract_exposures function in TwoSampleMR package to obtain the candidate IVs that were (nearly) independent across all the 15 traits (as required by current MVMR methods), leading to fewer IVs: 6 traits only had 6 or fewer IVs, and T2D only had one IV (with p-value<5e-8); this is a downside of using any current MVMR methods as discussed earlier. As shown in Table AH in S1 Text, 13 out of the 15 traits as exposures had a conditional F-statistic smaller than 10 (4.74 for BMI), suggesting that an MVMR analysis may suffer from weak instrument biases [22]. At the end, MVMR gave a similar conclusion: the estimated direct effect of BMI on CAD by MVMR-IVW was 0.07 with p-value 0.37, and it was 0.12 with p-value 0.06 by MVMR-robust. A recent study found a significant direct effect of BMI on CAD using MVMR-Robust, but with a smaller set of 6 risk factors, including Height, BMI, LDL, TG, SBP and HbA1c [62]. We further applied MVMR with the 5 exposures (excluding HbA1c), and the conditional F-statistics for BMI and SBP were still smaller than 10 (Table AI in S1 Text). Nevertheless, with this smaller set of risk factors, the estimated direct effect of BMI on CAD by MVMR-Robust was 0.20 with a significant p-value 6e-4, while by MVMR-IVW it was 0.13 with p-value 0.08. Lastly, our approach also suggested a significant direct effect of BMI on CAD (with an effect estimate 0.23 and p-value 1e-4) in this smaller network with the five traits and CAD.

## Discussion

It is always of interest to disentangle the causal relations among multiple traits, in which, for example, one can distinguish direct versus indirect/mediating causal effects. In this paper, we have proposed a general framework called Graph-MRcML to infer both a total causal (effect) network and a direct causal (effect) network. This framework has several merits. First, it allows for bidirectional edges or cycles in a directed graph, which is more likely to reflect true biological processes [63]. Second, using bidirectional MR to infer a total causal network alleviates the issue of unmeasured confounding and reverse causation with observational data. It does not require users to specify causal directions in advance. Third, many current methods of estimating causal networks require that the data for all the traits come from the same sample without hidden confounding [64, 65]; in contrast, our proposed framework can use GWAS data of traits from different (and possibly overlapping) samples with hidden confounding. Fourth, besides estimating both a total and a direct causal networks, the proposed data perturbation scheme allows for robust inference; that is, in addition to reconstructing a causal network, it allows testing the presence of an edge and constructing a confidence interval for any causal effect. Moreover, our proposed data perturbation scheme is both novel and effective by using a matrix normal distribution to effectively account for possible correlations among the SNPs (due to linkage disequilibrium) and among the traits simultaneously.

While our proposed framework is flexible in that it can use any bidirectional MR method to construct a total causal graph, we have focused on using MR-cML for its superior and robust performance. In particular, MR-cML is robust to both correlated and uncorrelated pleiotropic effects, while it possesses some nice statistical properties (e.g. estimation consistency and asymptotic normality) with impressive numerical performance. Furthermore, as shown in [16] both numerically and theoretically, with a simple IV screening procedure, MR-cML achieves good performance in inferring bi-directional causal directions under different scenarios with the exposure and the outcome being continuous and/or binary, as well as with some SNPs associated with a confounder of the two traits. However, it was originally proposed as a two-sample MR method. Nowadays, many large-scale GWAS were/are conducted by various consortia formed by many smaller studies with overlapping samples to maximize the total sample size and statistical power. Therefore, some SNP-exposure associations and SNP-outcome associations might be estimated from overlapping samples. To our best knowledge, except for a few newly proposed methods [13, 66, 67], most of the widely-used MR methods are based on two independent samples [15, 39, 68]. In this work, we have extended MR-cML to a more general set-up allowing sample overlap—it can be applied to two-sample, overlapping-two-sample and even one-sample set-ups. We have shown that all desirable statistical properties in the original version [15] carry over in this extended version of MR-cML. It is notable that we have also shown in the Supplementary that both the BIC selection consistency and the estimation consistency based on the original MR-cML-I in [15] (under the possibly incorrect assumption of no sample overlap) still hold in the presence of sample overlap; however, with realistic finite sample sizes, the extended MR-cML-C performed much better than MR-cML-I in the presence of sample overlap as shown in our simulation studies. Nevertheless, our numerical and theoretical results on MR-cML-I in the presence of sample overlap does suggest its (asymptotic) robustness, explaining why in practice it might be fine to ignore the issue of sample overlap if the proportion of overlapping is small.

There are a few limitations in this work. First, our proposed method is based on the classic statistical theory for a large sample size (*N*) and a fixed/small number of both IVs (*m*) and traits. This is suitable for a typical MR analysis with the sample sizes of GWAS data in tens to hundreds of thousands, while the number of IVs is often from tens to hundreds and that of traits in in low tens. However, if the number of (valid) IVs is small and/or the number of traits is large relative to the sample sizes, the finite-sample performance may go down with less precise estimates and loss of power. As shown in the simulations, in the presence of invalid IVs, the proposed method may yield biased estimates due to finite sample size; however, as shown by the theory, we expect it to give consistent selection of invalid IVs and consistent estimates as the sample size increases. Moreover, with a large number of traits, as in multiple regression, an issue similar to multicollinearity may appear or become more severe. In the future, it would be useful to incorporate variable selection to select and use only a subset of necessary traits to be included in the graph, though it may be then necessary to address the issue of post-selection inference, e.g. via data perturbation. Second, as in typical MR applications, we used the same GWAS sample to select significant SNPs as IVs and to estimate their association effects with the exposure. This will lead to the well-known “winner’s curse” or selection bias [69]. As in [66], we may account for the selection process by suitably adjusting the likelihood, which will be a future topic. Third, although the graph deconvolution algorithm is straightforward with a closed-form solution, the key assumption is that a direct causal graph has a spectral radius smaller than one. In practice, this assumption may be violated for an estimated direct causal graph, e.g. due to errors in estimating the corresponding total causal graph. However, when such a violation is not severe, the estimated direct causal graph might still be useful. Furthermore, because our proposed estimators for both the total and direct graphs are consistent (when the assumptions for MR-cML and graph deconvolution hold), it is expected that increasing sample sizes of GWAS will alleviate the potential problem. Fourth, in the real data analysis, due to the fact that one’s birth weight cannot be affected by any traits developed/measured in a later time, the (true) total causal effects of any latter traits to birth weight should be zeros, and we set them as zeros in the estimated total causal graph. While such a practice is not necessary when applying our proposed method, we expect that, by taking advantage of this prior knowledge, doing so would perform better for finite samples. Finally, as different MR methods rely on their own assumptions as well as the quality of genetic variants as IVs, more applications to real data, including applying alternative MR methods would be warranted as a means of causal triangulation.

## Supporting information

S1 Text Supplementary file with theoretical results and proofs, details of methods, additional real data analysis results and additional simulation results. (PDF)
