# Supplementary material for: Combining Mendelian randomization and network deconvolution for inference of causal networks with GWAS summary data
Source: PLoS Genet. 2023 May 18;19(5):e1010762. doi: 10.1371/journal.pgen.1010762 (PMC10231771; doi:10.1371/journal.pgen.1010762)
Supplement: S1 Text — (PDF) [file pgen.1010762.s001.pdf]

Supplementary materials: Combining Mendelian randomization and network deconvolution for inference of causal networks with GWAS summary data

A Theory

A.1 Large-sample theory

The estimation and selection consistency (of BIC) with the cMLE developed in [1] can be carried over to the set-up with overlapping samples. Here we first state the main conclusions with the proofs provided in the following subsections.

Denote $\hat{\theta} = \hat{\theta}(\hat{K})$ the cMLE using the set of selected valid IVs $\hat{B}^C_{\hat{K}}$. Now we state three assumptions used to prove the estimation consistency and asymptotic normality of our proposed cMLE $\hat{\theta}$.

Assumption 1. *For every SNP $i = 1, \ldots, m$, $\binom{\hat{\beta}_{Xi}}{\hat{\beta}_{Yi}} \sim \mathcal{N}\left(\binom{b_{Xi}}{b_{Yi}}, \begin{pmatrix} \sigma^2_{Xi} & \rho\sigma_{Xi}\sigma_{Yi} \\ \rho\sigma_{Xi}\sigma_{Yi} & \sigma^2_{Yi} \end{pmatrix}\right)$ with the known variances $(\sigma^2_{Xi}, \sigma^2_{Yi})$ and correlation ρ. Furthermore, the m pairs of $(\hat{\beta}_{Xi}, \hat{\beta}_{Yi})^m_{i=1}$ are mutually independent.*

Assumption 2. *(Plurality valid condition.) Suppose that B_0 is the index set of the true invalid IVs with $K_0 = |B_0|$. For any $B \subseteq \{1, \ldots, m\}$ and $|B| = K_0$, if $B \neq B_0$, then the $(m - K_0)$ ratios $\{r_i/b_{Xi}, i \in B^c\}$ are not all equal.*

Assumption 3. *(Orders of the variances and sample sizes.) There exist positive constants l_X, l_Y, l_N and u_X, u_Y, u_N such that we have $l_X/N_1 \leq \sigma^2_{Xi} \leq u_X/N_1$, $l_Y/N_2 \leq \sigma^2_{Yi} \leq u_Y/N_2$, and $l_N \cdot N_2 \leq N_1 \leq u_N \cdot N_2$ for $i = 1, \ldots, m$.*

It is noted that Assumption 1 is reasonable given the usual large sample sizes of GWAS. As usual, for each pair of traits, the m SNPs are selected to be independent, implying that their corresponding SNP-trait association estimates are nearly independent [2]. As discussed

in [1], Assumption 2 is relatively weak as compared to those adopted by many other MR methods; in particular, it is weaker than the majority valid assumption (i.e. at least more than 50% of IVs are valid); as usual, if GWAS summary data are based on the MLEs, Assumption 3 holds.

Theorem 1. *With Assumptions 1 to 3 satisfied, the proposed BIC consistently select invalid IVs, and the proposed constrained maximum likelihood estimator $\hat{\theta}$, combined with the use of the BIC selection criterion, is consistent for the true causal effect size θ_0, and*

$$\sqrt{V^*}(\hat{\theta} - \theta_0) \xrightarrow{d} \mathcal{N}(0, 1) \text{ as } N \to \infty,$$

where

$$V^* = \sum_{i \in \hat{B}_{\hat{K}}^C} \frac{b_{Xi}^2 \sigma_{Yi}^2 + b_{Yi}^2 \sigma_{Xi}^2 - 2\rho b_{Xi} b_{Yi} \sigma_{Xi} \sigma_{Yi}}{(\sigma_{Yi}^2 + \theta_0^2 \sigma_{Xi}^2 - 2\rho\theta_0 \sigma_{Xi}\sigma_{Yi})^2}$$

is consistently estimated by substituting θ, b_{Xi} and b_{Yi} with $\hat{\theta}$, $\hat{\beta}_{Xi}$ and $\hat{\beta}_{Yi}$ respectively.

To prove Theorem 1, we first show that the selection consistency is achieved by the proposed BIC. Then after correctly selecting out invalid IVs, the resulting cMLE of θ is the same as the maximum profile likelihood estimator being applied to all valid IVs, obtaining its estimation consistency and asymptotic normality similar to Theorems 3.1 and 3.2 in [2]. The details are given in Section A.2. We also show in Section A.3 that the Fisher information-based variance estimator of the cMLE used in our method and the one in Theorem 1 are asymptotically equivalent.

We note that the selection consistency of BIC in MR-cML-BIC-I as proposed in [1] still holds despite the violation of its assumption due to the presence of sample overlap, so do the estimation consistency and asymptotic normality of MR-cML-BIC-I. However, the corresponding estimation efficiency is lower than that of MR-cML-BIC-C. Furthermore, the usual (naive or model-based) variance estimator in [1] is not consistent; instead, we propose a robust variance estimator. Here we state the conclusion with the details given in Section A.4.

Theorem 2. *With Assumptions 1 to 3 satisfied, under the (mis-specified) working independence model as proposed in [1], the BIC can still consistently select invalid IVs, and the constrained maximum likelihood estimator $\hat{\theta}_I$, combined with the use of the BIC selection criterion, is consistent for the true causal effect size θ_0, and*

$$\sqrt{V_r}(\hat{\theta}_I - \theta_0) \xrightarrow{d} \mathcal{N}(0,1) \text{ as } N \to \infty,$$

where

$$V_r^{-1} = \mathbb{E}[\psi'(\theta_0; \rho = 0)]^{-1}\mathbb{E}[\psi^2(\theta_0; \rho = 0)]\mathbb{E}[\psi'(\theta_0; \rho = 0)]^{-1},$$

and $\psi(\theta; \rho = 0) = \partial \max_{\mathbf{b}_X} l(\theta, \mathbf{b}_X; \rho = 0)/\partial\theta$ is the profile likelihood score under the working independence model (i.e., $\rho = 0$); V_r can be consistently estimated by its sample version (as the sandwich estimator).

Since we apply the proposed MR-cML-BIC-C on every pair of traits to construct a total causal graph, we'd expect that the consistency and asymptotic normality can be obtained for the estimated total causal graph and direct causal graph as stated below. We are now considering the GWAS summary data from all traits together with possibly correlated SNPs as IVs, requiring a joint normal distribution of all the SNP-trait association estimates, which is reasonable based on large-scale GWAS data.

Assumption 4. *The vector of all SNP-trait association estimates $\hat{\beta}_{Xi}$'s and $\hat{\beta}_{Yi}$'s (across all traits X and Y and across all SNPs i) has a multivariate normal distribution.*

Assumption 5. $\mathbf{G}_{tot} = \mathbf{G}_{dir} + \mathbf{G}_{dir}^2 + \mathbf{G}_{dir}^3 + \cdots = \mathbf{G}_{dir}(\mathbf{I} - \mathbf{G}_{dir})^{-1}$.

Corollary 1. *With Assumptions 1 to 5 satisfied, if the diagonal elements of $\widehat{\mathbf{G}}_{tot}$ are set consistently, then vec($\widehat{\mathbf{G}}_{tot}$) and vec($\widehat{\mathbf{G}}_{dir}$) are consistent for vec($\mathbf{G}_{tot}$) and vec($\mathbf{G}_{dir}$) respectively, and are asymptotically normally distributed.*

A.2 Proof of Theorem 1

To prove the estimation consistency and asymptotic normality of the cMLE, we will first prove the selection consistency of BIC following the approach in the Supplementary S1 in [1], i.e., it will select the correct set of invalid IVs as the sample size N goes to infinity. Then we prove the estimation consistency and asymptotic normality based on the set of (selected) valid IVs.

Lemma 1. *With Assumptions 1 to 3 satisfied, if* $K_0 \in \mathcal{K}$*, we have* $P(\hat{K} = K_0) \to 1$ *and* $P(\hat{B}_{\hat{K}} = B_0) \to 1$ *as* $N_1, N_2 \to \infty$*.*

Proof. First, we show $P(\hat{B}_{K_0} = B_0) \to 1$, which is equivalent to show for any $B_1 \subseteq \{1, \cdots, m\}$ such that $|B_1| = K_0$ and $B_1 \neq B_0$, $P(\hat{B}_{K_0} = B_1) \to 0$ as $N_1, N_2 \to \infty$. We have

$$
\begin{aligned}
&P(\hat{B}_{K_0} = B_1) \\
&\leq P\Big\{ \min_{\tilde{\theta}, \tilde{b}_{Xi}} \sum_{i \in B_1^c} \left(\frac{(\hat{\beta}_{Xi} - \tilde{b}_{Xi})^2}{\sigma_{Xi}^2} + \frac{(\hat{\beta}_{Yi} - \tilde{\theta}\tilde{b}_{Xi})^2}{\sigma_{Yi}^2} - 2\rho \frac{(\hat{\beta}_{Xi} - \tilde{b}_{Xi})(\hat{\beta}_{Yi} - \tilde{\theta}\tilde{b}_{Xi})}{\sigma_{Xi}\sigma_{Yi}} \right) \\
&\qquad \leq \sum_{i \in B_0^c} \left(\frac{(\hat{\beta}_{Xi} - b_{Xi})^2}{\sigma_{Xi}^2} + \frac{(\hat{\beta}_{Yi} - \theta b_{Xi})^2}{\sigma_{Yi}^2} - 2\rho \frac{(\hat{\beta}_{Xi} - b_{Xi})(\hat{\beta}_{Yi} - \theta b_{Xi})}{\sigma_{Xi}\sigma_{Yi}} \right) \Big\}.
\end{aligned}
$$

Note that, for $i \in B_0^c$, $\left(\begin{smallmatrix} \hat{\beta}_{Xi} - b_{Xi} \\ \hat{\beta}_{Yi} - \theta b_{Xi} \end{smallmatrix}\right) \sim \mathcal{N}\Big(\mathbf{0}, \left(\begin{smallmatrix} \sigma_{Xi}^2 & \rho\sigma_{Xi}\sigma_{Yi} \\ \rho\sigma_{Xi}\sigma_{Yi} & \sigma_{Yi}^2 \end{smallmatrix}\right)\Big)$. So for any $\epsilon > 0$, there exists $C > 0$ such that

$$
P\Big\{ \sum_{i \in B_0^c} \left(\frac{(\hat{\beta}_{Xi} - b_{Xi})^2}{\sigma_{Xi}^2} + \frac{(\hat{\beta}_{Yi} - \theta b_{Xi})^2}{\sigma_{Yi}^2} - 2\rho \frac{(\hat{\beta}_{Xi} - b_{Xi})(\hat{\beta}_{Yi} - \theta b_{Xi})}{\sigma_{Xi}\sigma_{Yi}} \right) > C \Big\} < \frac{\epsilon}{2}. \qquad (1)
$$

And we have

$$
\begin{aligned}
&P\Big\{\min_{\tilde\theta,\tilde b_{Xi}}\sum_{i\in B_1^c}\left(\frac{(\hat\beta_{Xi}-\tilde b_{Xi})^2}{\sigma_{Xi}^2}+\frac{(\hat\beta_{Yi}-\tilde\theta\tilde b_{Xi})^2}{\sigma_{Yi}^2}-2\rho\frac{(\hat\beta_{Xi}-\tilde b_{Xi})(\hat\beta_{Yi}-\tilde\theta\tilde b_{Xi})}{\sigma_{Xi}\sigma_{Yi}}\right)\\
&\qquad\le\sum_{i\in B_0^c}\left(\frac{(\hat\beta_{Xi}-b_{Xi})^2}{\sigma_{Xi}^2}+\frac{(\hat\beta_{Yi}-\theta b_{Xi})^2}{\sigma_{Yi}^2}-2\rho\frac{(\hat\beta_{Xi}-b_{Xi})(\hat\beta_{Yi}-\theta b_{Xi})}{\sigma_{Xi}\sigma_{Yi}}\right)\Big\}\\
&\le P\Big\{\min_{\tilde\theta,\tilde b_{Xi}}\sum_{i\in B_1^c}\left(\frac{(\hat\beta_{Xi}-\tilde b_{Xi})^2}{\sigma_{Xi}^2}+\frac{(\hat\beta_{Yi}-\tilde\theta\tilde b_{Xi})^2}{\sigma_{Yi}^2}-2\rho\frac{(\hat\beta_{Xi}-b_{Xi})(\hat\beta_{Yi}-\theta b_{Xi})}{\sigma_{Xi}\sigma_{Yi}}\right)\le C\Big\}\\
&\quad+P\Big\{\sum_{i\in B_0^c}\left(\frac{(\hat\beta_{Xi}-\beta_{Xi})^2}{\sigma_{Xi}^2}+\frac{(\hat\beta_{Yi}-\theta\beta_{Xi})^2}{\sigma_{Yi}^2}-2\rho\frac{(\hat\beta_{Xi}-b_{Xi})(\hat\beta_{Yi}-\theta b_{Xi})}{\sigma_{Xi}\sigma_{Yi}}\right)>C\Big\}.
\end{aligned}
$$

After profiling out $\tilde b_{Xi}$'s, we get

$$
\begin{aligned}
&\min_{\tilde\theta,\tilde b_{Xi}}\sum_{i\in B_1^c}\left(\frac{(\hat\beta_{Xi}-\tilde b_{Xi})^2}{\sigma_{Xi}^2}+\frac{(\hat\beta_{Yi}-\tilde\theta\tilde b_{Xi})^2}{\sigma_{Yi}^2}-2\rho\frac{(\hat\beta_{Xi}-\tilde b_{Xi})(\hat\beta_{Yi}-\tilde\theta\tilde b_{Xi})}{\sigma_{Xi}\sigma_{Yi}}\right)\\
&=\min_{\tilde\theta}\sum_{i\in B_1^c}\frac{(\hat\beta_{Yi}-\tilde\theta\cdot\hat\beta_{Xi})^2(1-\rho^2)}{\sigma_{Yi}^2+\tilde\theta^2\sigma_{Xi}^2-2\rho\tilde\theta\sigma_{Xi}\sigma_{Yi}},
\end{aligned}
$$

so

$$
\begin{aligned}
&P\Big\{\min_{\tilde\theta,\tilde b_{Xi}}\sum_{i\in B_1^c}\left(\frac{(\hat\beta_{Xi}-\tilde b_{Xi})^2}{\sigma_{Xi}^2}+\frac{(\hat\beta_{Yi}-\tilde\theta\tilde b_{Xi})^2}{\sigma_{Yi}^2}-2\rho\frac{(\hat\beta_{Xi}-b_{Xi})(\hat\beta_{Yi}-\theta b_{Xi})}{\sigma_{Xi}\sigma_{Yi}}\right)\le C\Big\}\\
&=P\Big\{\min_{\tilde\theta}\sum_{i\in B_1^c}\frac{(\hat\beta_{Yi}-\tilde\theta\cdot\hat\beta_{Xi})^2}{\sigma_{Yi}^2+\tilde\theta^2\sigma_{Xi}^2-2\rho\tilde\theta\sigma_{Xi}\sigma_{Yi}}\le C/(1-\rho^2)\Big\}.
\end{aligned}
$$

We have $\frac{\hat\beta_{Yi}-\tilde\theta\cdot\hat\beta_{Xi}}{\sqrt{\sigma_{Yi}^2+\tilde\theta^2\sigma_{Xi}^2-2\rho\tilde\theta\sigma_{Xi}\sigma_{Yi}}}\sim\mathcal{N}(\frac{\theta\cdot b_{Xi}+r_i-\tilde\theta\cdot b_{Xi}}{\sqrt{\sigma_{Yi}^2+\tilde\theta^2\sigma_{Xi}^2-2\rho\tilde\theta\sigma_{Xi}\sigma_{Yi}}},1)$, so $\sum_{i\in B_1^c}\frac{(\hat\beta_{Yi}-\tilde\theta\cdot\hat\beta_{Xi})^2}{\sigma_{Yi}^2+\tilde\theta^2\sigma_{Xi}^2-2\rho\tilde\theta\sigma_{Xi}\sigma_{Yi}}$ follows non-central χ^2 distribution with degrees of freedom $(m-K_0)$ and non-centrality parameter $\lambda_{\tilde\theta}$ depending on $\tilde\theta$

$$
\lambda_{\tilde\theta}=\sum_{i\in B_1^c}\frac{(\theta\cdot b_{Xi}+r_i-\tilde\theta\cdot b_{Xi})^2}{\sigma_{Yi}^2+\tilde\theta^2\sigma_{Xi}^2-2\rho\tilde\theta\sigma_{Xi}\sigma_{Yi}}.
$$

With Assumption 3, we get

$$\lambda_{\tilde{\theta}} \geq \sum_{i \in B_1^c} \frac{(\theta \cdot b_{Xi} + r_i - \tilde{\theta} \cdot b_{Xi})^2}{\frac{u_Y}{N_2} + \tilde{\theta}^2 \cdot \frac{u_X}{l_N \cdot N_2} + 2|\tilde{\theta}| \frac{\sqrt{u_X u_Y}}{N_2 \sqrt{l_N}}} = N_2 \cdot \sum_{i \in B_1^c} \frac{(\theta \cdot b_{Xi} + r_i - \tilde{\theta} \cdot b_{Xi})^2}{u_Y + \tilde{\theta}^2 \cdot \frac{u_X}{l_N} + 2|\tilde{\theta}| \sqrt{u_X u_Y / l_N}}.$$

With Assumption 2, we know

$$\min_{\tilde{\theta}} \sum_{i \in B_1^c} \frac{(\theta \cdot b_{Xi} + r_i - \tilde{\theta} \cdot b_{Xi})^2}{u_Y + \tilde{\theta}^2 \cdot \frac{u_X}{l_N} + 2|\tilde{\theta}| \sqrt{u_X u_Y / l_N}} = v > 0,$$

here v is a constant. This is because, with Assumption 2, there is no $\tilde{\theta}$ making $\theta \cdot b_{Xi} + r_i - \tilde{\theta} \cdot b_{Xi} = 0$ for all $i \in B_1^c$ simultaneously. So we have $\min_{\tilde{\theta}} \lambda_{\tilde{\theta}} \geq N_2 \cdot v$. Then as N_2 large enough, we have

$$P\Big\{ \min_{\tilde{\theta}} \sum_{i \in B_1^c} \frac{(\hat{\beta}_{Yi} - \tilde{\theta} \cdot \hat{\beta}_{Xi})^2}{\sigma_{Yi}^2 + \tilde{\theta}^2 \sigma_{Xi}^2 - 2\rho \tilde{\theta} \sigma_{Xi} \sigma_{Yi}} \leq C/(1-\rho^2) \Big\} \leq \frac{\epsilon}{2}. \tag{2}$$

Combining (1) and (2), we get $P(\hat{B}_{K_0} = B_0) \to 1$ as $N_1, N_2 \to \infty$.

Next, we show $P(\hat{K} = K_0) \to 1$. For any $K_1 < K_0$, we have

$$\begin{aligned} P(\hat{K} = K_1) &\leq P\big\{ \text{BIC}(K_1) \leq \text{BIC}(K_0) \big\} \\ &= P\Big\{ 2l\left(\hat{\theta}(K_0), \hat{b}_{Xi}(K_0), \hat{r}_i(K_0)\right) - 2l\left(\hat{\theta}(K_1), \hat{b}_{Xi}(K_1), \hat{r}_i(K_1)\right) \leq \log(N)(K_0 - K_1) \Big\}. \end{aligned}$$

As we have shown $P(\hat{B}_{K_0} = B_0) \to 1$, with probability goes to 1 we have

$$\begin{aligned} & 2l\left(\hat{\theta}(K_0), \hat{b}_{Xi}(K_0), \hat{r}_i(K_0)\right) - 2l\left(\hat{\theta}(K_1), \hat{b}_{Xi}(K_1), \hat{r}_i(K_1)\right) \\ \geq & \min_{\tilde{\theta}, \tilde{b}_{Xi}} \sum_{i \in \hat{B}_{K_1}^c} \left(\frac{(\hat{\beta}_{Xi} - \tilde{b}_{Xi})^2}{\sigma_{Xi}^2} + \frac{(\hat{\beta}_{Yi} - \tilde{\theta}\tilde{b}_{Xi})^2}{\sigma_{Yi}^2} - 2\rho \frac{(\hat{\beta}_{Xi} - \tilde{b}_{Xi})(\hat{\beta}_{Yi} - \tilde{\theta}\tilde{b}_{Xi})}{\sigma_{Xi}\sigma_{Yi}} \right) \\ & - \sum_{i \in B_0^c} \left(\frac{(\hat{\beta}_{Xi} - b_{Xi})^2}{\sigma_{Xi}^2} + \frac{(\hat{\beta}_{Yi} - \theta b_{Xi})^2}{\sigma_{Yi}^2} - 2\rho \frac{(\hat{\beta}_{Xi} - b_{Xi})(\hat{\beta}_{Yi} - \theta b_{Xi})}{\sigma_{Xi}\sigma_{Yi}} \right). \end{aligned}$$

Then we get

$$
\begin{aligned}
P(\hat{K}=K_1) \le \sum_{|B|=K_1} P\Big\{ \min_{\tilde{\theta},\tilde{b}_{Xi}} \sum_{i\in B^c} & \left(\frac{(\hat{\beta}_{Xi}-\tilde{b}_{Xi})^2}{\sigma_{Xi}^2} + \frac{(\hat{\beta}_{Yi}-\tilde{\theta}\tilde{b}_{Xi})^2}{\sigma_{Yi}^2} - 2\rho \frac{(\hat{\beta}_{Xi}-\tilde{b}_{Xi})(\hat{\beta}_{Yi}-\tilde{\theta}\tilde{b}_{Xi})}{\sigma_{Xi}\sigma_{Yi}} \right) \\
& \le \sum_{i\in B_0^c} \left(\frac{(\hat{\beta}_{Xi}-b_{Xi})^2}{\sigma_{Xi}^2} + \frac{(\hat{\beta}_{Yi}-\theta b_{Xi})^2}{\sigma_{Yi}^2} - 2\rho \frac{(\hat{\beta}_{Xi}-b_{Xi})(\hat{\beta}_{Yi}-\theta b_{Xi})}{\sigma_{Xi}\sigma_{Yi}} \right) \\
& \quad + \log(N)(K_0-K_1)(1-\rho^2) \Big\}.
\end{aligned}
$$

Similar as above, we get

$$
\begin{aligned}
& \min_{\tilde{\theta},\tilde{b}_{Xi}} \sum_{i\in B^c} \left(\frac{(\hat{\beta}_{Xi}-\tilde{b}_{Xi})^2}{\sigma_{Xi}^2} + \frac{(\hat{\beta}_{Yi}-\tilde{\theta}\tilde{b}_{Xi})^2}{\sigma_{Yi}^2} - 2\rho \frac{(\hat{\beta}_{Xi}-\tilde{b}_{Xi})(\hat{\beta}_{Yi}-\tilde{\theta}\tilde{b}_{Xi})}{\sigma_{Xi}\sigma_{Yi}} \right) \\
& = \min_{\tilde{\theta}} \sum_{i\in B^c} \frac{(\hat{\beta}_{Yi}-\tilde{\theta}\cdot\hat{\beta}_{Xi})^2(1-\rho^2)}{\sigma_{Yi}^2+\tilde{\theta}^2\sigma_{Xi}^2-2\rho\tilde{\theta}\sigma_{Xi}\sigma_{Yi}},
\end{aligned}
$$

and $\sum_{i\in B^c} \frac{(\hat{\beta}_{Yi}-\tilde{\theta}\cdot\hat{\beta}_{Xi})^2}{\sigma_{Yi}^2+\tilde{\theta}^2\sigma_{Xi}^2-2\rho\tilde{\theta}\sigma_{Xi}\sigma_{Yi}}$ follows non-central χ^2 distribution with degrees of freedom $(m-K_1)$ and non-centrality parameter $\lambda_{\tilde{\theta}}$ depending on $\tilde{\theta}$

$$
\lambda_{\tilde{\theta}} = \sum_{i\in B^c} \frac{(\theta\cdot b_{Xi}+r_i-\tilde{\theta}\cdot b_{Xi})^2}{\sigma_{Yi}^2+\tilde{\theta}^2\sigma_{Xi}^2-2\rho\tilde{\theta}\sigma_{Xi}\sigma_{Yi}}.
$$

Similarly, since $K_1 < K_0$, and with Assumption 3 we have $\lambda_{\tilde{\theta}} \ge N_2\cdot v$ for some positive constant v, so for any $|B|=K_1$, we get

$$
\begin{aligned}
P\Big\{ \min_{\tilde{\theta},\tilde{b}_{Xi}} \sum_{i\in B^c} & \left(\frac{(\hat{\beta}_{Xi}-\tilde{b}_{Xi})^2}{\sigma_{Xi}^2} + \frac{(\hat{\beta}_{Yi}-\tilde{\theta}\tilde{b}_{Xi})^2}{\sigma_{Yi}^2} - 2\rho \frac{(\hat{\beta}_{Xi}-\tilde{b}_{Xi})(\hat{\beta}_{Yi}-\tilde{\theta}\tilde{b}_{Xi})}{\sigma_{Xi}\sigma_{Yi}} \right) \\
\le \sum_{i\in B_0^c} & \left(\frac{(\hat{\beta}_{Xi}-\beta_{Xi})^2}{\sigma_{Xi}^2} + \frac{(\hat{\beta}_{Yi}-\theta\beta_{Xi})^2}{\sigma_{Yi}^2} - 2\rho \frac{(\hat{\beta}_{Xi}-b_{Xi})(\hat{\beta}_{Yi}-\theta b_{Xi})}{\sigma_{Xi}\sigma_{Yi}} \right) \\
& + \log(N)(K_0-K_1)(1-\rho^2) \Big\} \to 0
\end{aligned}
$$

This gives us $P(\hat{K} = K_1) \to 0$ for any $K_1 < K_0$. For any $K_1 > K_0$, we have

$$
\begin{aligned}
&P(\hat{K} = K_1) \\
\leq &P\Big\{ \log(N)(K_1 - K_0) \\
&\quad \leq \sum_{i \in B_0^c} \frac{1}{1-\rho^2} \left(\frac{(\hat{\beta}_{Xi} - \beta_{Xi})^2}{\sigma_{Xi}^2} + \frac{(\hat{\beta}_{Yi} - \theta\beta_{Xi})^2}{\sigma_{Yi}^2} - 2\rho \frac{(\hat{\beta}_{Xi} - b_{Xi})(\hat{\beta}_{Yi} - \theta b_{Xi})}{\sigma_{Xi}\sigma_{Yi}} \right) \Big\}
\end{aligned}
$$

Since $\sum_{i \in B_0^c} \frac{1}{1-\rho^2} \left(\frac{(\hat{\beta}_{Xi} - \beta_{Xi})^2}{\sigma_{Xi}^2} + \frac{(\hat{\beta}_{Yi} - \theta\beta_{Xi})^2}{\sigma_{Yi}^2} - 2\rho \frac{(\hat{\beta}_{Xi} - b_{Xi})(\hat{\beta}_{Yi} - \theta b_{Xi})}{\sigma_{Xi}\sigma_{Yi}} \right)$ is a weighted sum of χ_1^2, we get $P(\hat{K} = K_1) \to 0$ for any $K_1 > K_0$. So we have $P(\hat{K} = K_0) \to 1$ as $N_1, N_2 \to \infty$. $\square$

After correctly selecting invalid IVs, the resulted cMLE of θ is the same as the maximum profile likelihood estimator (MPLE) (profiling out $\mathbf{b}_{XB}$) being applied to all (selected) valid IVs. And we will prove the consistency and asymptotic normality based on the MPLE. For the simplicity of notation, we assume all m IVs are valid from now on. We follow the similar proofs given in [2], but we only consider the 'fixed m large N' scenario here.

Under the theoretical model that m IVs are valid (i.e., $b_{Yi} = \theta b_{Xi}$) and Assumption 1, the log-likelihood function is given by

$$
l(\theta, b_{X1}, \ldots, b_{Xm}) = -\frac{1}{2(1-\rho^2)} \sum_{i=1}^{m} \Big(\frac{(\hat{\beta}_{Xi} - b_{Xi})^2}{\sigma_{Xi}^2} + \frac{(\hat{\beta}_{Yi} - \theta b_{Xi})^2}{\sigma_{Yi}^2} - 2\rho \frac{(\hat{\beta}_{Xi} - b_{Xi})}{\sigma_{Xi}} \frac{(\hat{\beta}_{Yi} - \theta b_{Xi})}{\sigma_{Yi}} \Big). \quad (3)
$$

The true causal effect θ_0 is of interest, and $\mathbf{b}_X = (b_{X1}, \ldots, b_{Xm})$ are treated as nuisance parameters. Then the profile log-likelihood of θ is given by profiling out $\mathbf{b}_X$ in Eq (3):

$$
l_p(\theta) = \max_{\mathbf{b}_X} l(\theta, \mathbf{b}_X) = -\frac{1}{2} \sum_{i=1}^{m} \frac{(\hat{\beta}_{Yi} - \theta\hat{\beta}_{Xi})^2}{\sigma_{Yi}^2 + \theta^2\sigma_{Xi}^2 - 2\rho\theta\sigma_{Xi}\sigma_{Yi}}. \quad (4)
$$

The maximum likelihood estimator of θ is given by $\hat{\theta} = \arg\max_\theta l_p(\theta)$.

It's noted that since we consider a fixed number of IVs, we have $||\mathbf{b}_X||_2^2 = O(1)$ (Assumption 2 in [2]). Furthermore, Assumption 3 implies that there exists constants c_σ, c'_σ such that $c_\sigma/N \leq \sigma_{Xi}^2 \leq c'_\sigma/N$ and $c_\sigma/N \leq \sigma_{Yi}^2 \leq c'_\sigma/N$ for $i = 1, \ldots, m$ (Assumption 3 in [2]). Now

we are ready to state the estimation consistency of $\hat{\theta}$.

Lemma 2. *Under the model that all m IVs are valid and Assumptions 1-3, the maximum likelihood estimator $\hat{\theta}$ is consistent, that is, $\hat{\theta} \xrightarrow{p} \theta_0$ as $N \to \infty$.*

Proof. Let $e_i = \hat{\beta}_{Yi} - b_{Yi}$, $\epsilon_i = \hat{\beta}_{Xi} - b_{Xi}$. After some algebra, we have

$$l_p(\theta) = -\frac{1}{2}\sum_{i=1}^{m} \frac{b_{Xi}^2(\theta_0-\theta)^2 + (e_i - \theta\epsilon_i)^2 + 2b_{Xi}(\theta_0-\theta)(e_i-\theta\epsilon_i)}{\sigma_{Yi}^2 + \theta^2\sigma_{Xi}^2 - 2\rho\theta\sigma_{Xi}\sigma_{Yi}}.$$

Notice that $e_i - \theta\epsilon_i \sim \mathcal{N}(0, \theta^2\sigma_{Xi}^2 + \sigma_{Yi}^2 - 2\theta\rho\sigma_{Xi}\sigma_{Yi})$. Follow the same argument in [2], we have

$$-2l_p(\theta) \geq \frac{N||\mathbf{b}_X||_2^2}{2c_\sigma}\min\left((\theta_0-\theta)^2, \frac{(\theta_0-\theta)^2}{\theta^2+2|\theta|}\right) + m + O_p(\sqrt{m} + \sqrt{N}||\mathbf{b}_X||\cdot|\theta_0-\theta|).$$

Similarly, we can show that for any $\epsilon > 0$ there exists constant $C(\theta_0, \epsilon) > 0$ such that $\inf_{|\theta-\theta_0|>\epsilon}(\theta_0-\theta)^2/(\theta^2+2|\theta|) \geq C(\theta_0,\epsilon)$. And the last term $O_p(\sqrt{N}||\mathbf{b}_X||\cdot|\theta_0-\theta|)$ is negligible compared to the first term when $|\theta-\theta_0| > \epsilon$. Let $C'(\theta_0,\epsilon) = \min(\epsilon^2, C(\theta_0,\epsilon)) > 0$, we have

$$\inf_{|\theta-\theta_0|>\epsilon} -2l_p(\theta) \geq (1+o_p(1))C'(\theta_0,\epsilon)\frac{N||\mathbf{b}_X||_2^2}{2c_\sigma} + m + O_p(\sqrt{m}),$$

and $-2l_p(\theta_0) = m + O_p(\sqrt{m})$. Therefore,

$$P\left(l_p(\theta_0) > \sup_{|\theta-\theta_0|>\epsilon} l_p(\theta)\right) = P\left(O_p(\sqrt{m}) \leq (1+o_p(1))C'(\theta_0,\epsilon)\frac{N||\mathbf{b}_X||_2^2}{2c_\sigma} + O_p(\sqrt{m})\right).$$

When $N \to \infty$ (and thus $m/(N^2||\mathbf{b}_X||_2^4) \to 0$), this probability converges to 1. □

Next, we study the asymptotic normality of $\hat{\theta}$. Define the profile score to be the derivative

of the profile log-likelihood:

$$\psi(\theta) := l_p'(\theta) = \sum_{i=1}^{m} \frac{(\hat{\beta}_{Yi} - \theta\hat{\beta}_{Xi})(\hat{\beta}_{Xi}\sigma_{Yi}^2 + \hat{\beta}_{Yi}\sigma_{Xi}^2\theta - \rho\sigma_{Xi}\sigma_{Yi}(\hat{\beta}_{Xi}\theta + \hat{\beta}_{Yi}))}{(\sigma_{Yi}^2 + \theta^2\sigma_{Xi}^2 - 2\rho\theta\sigma_{Xi}\sigma_{Yi})^2}. \tag{5}$$

The Taylor expansion of $\psi(\hat{\theta})$ around the truth θ_0 can be expressed as:

$$0 = \psi(\hat{\theta}) = \psi(\theta_0) + \psi'(\theta_0)(\hat{\theta} - \theta_0) + \frac{1}{2}\psi''(\tilde{\theta})(\hat{\theta} - \theta_0)^2,$$

where $\tilde{\theta}$ is between $\hat{\theta}$ and θ_0. Then we have

$$\sqrt{V^*}(\hat{\theta} - \theta_0) = \frac{-\psi(\theta_0)/\sqrt{V^*}}{\psi'(\theta_0)/V^* + (1/2)\psi''(\tilde{\theta})(\hat{\theta} - \theta_0)/V^*}, \tag{6}$$

where

$$V^* = \mathbb{E}[-\psi'(\theta_0)] = \sum_{i=1}^{m} \frac{b_{Xi}^2\sigma_{Yi}^2 + b_{Yi}^2\sigma_{Xi}^2 - 2\rho b_{Xi}b_{Yi}\sigma_{Xi}\sigma_{Yi}}{(\sigma_{Yi}^2 + \theta_0^2\sigma_{Xi}^2 - 2\rho\theta_0\sigma_{Xi}\sigma_{Yi})^2}. \tag{7}$$

The nominator of Eq (6) can be proved to converge in distribution to $\mathcal{N}(0,1)$, the first term in the denominator of Eq (6) can be proved to converge in probability to 1 and the second term in the denominator an be proved to be negligible given $\hat{\theta}$ is consistent. We first prove these three statements, and by Slutsky's Theorem, the following asymptotic normality of $\hat{\theta}$ can be established.

Lemma 3. *Under the assumptions in Lemma 2, we have*

$$\sqrt{V^*}(\hat{\theta} - \theta_0) \xrightarrow{d} \mathcal{N}(0,1),$$

where

$$V^* = \sum_{i=1}^{m} \frac{b_{Xi}^2\sigma_{Yi}^2 + b_{Yi}^2\sigma_{Xi}^2 - 2\rho b_{Xi}b_{Yi}\sigma_{Xi}\sigma_{Yi}}{(\sigma_{Yi}^2 + \theta_0^2\sigma_{Xi}^2 - 2\rho\theta_0\sigma_{Xi}\sigma_{Yi})^2}.$$

Proof. We first show $(1/\sqrt{V^*})\psi(\theta_0) \xrightarrow{d} \mathcal{N}(0,1)$.

We can rewrite Eq (5) to obtain

$$\psi(\theta_0) = \sum_{i=1}^{m} \frac{(e_i - \theta_0\epsilon_i)b_{Xi}}{\sigma_{Yi}^2 + \theta_0^2\sigma_{Xi}^2 - 2\rho\theta_0\sigma_{Xi}\sigma_{Yi}} + \sum_{i=1}^{m} \frac{(e_i - \theta_0\epsilon_i)(\epsilon_i\sigma_{Yi}^2 + e_i\sigma_{Xi}^2\theta_0 - \rho\sigma_{Xi}\sigma_{Yi}(\epsilon_i\theta_0 + e_i))}{(\sigma_{Yi}^2 + \theta_0^2\sigma_{Xi}^2 - 2\rho\theta_0\sigma_{Xi}\sigma_{Yi})^2}$$
$$:= \sum_{i=1}^{m}\psi_{1i} + \sum_{i=1}^{m}\psi_{2i}. \tag{8}$$

Since $\mathbb{E}[e_i - \theta_0\epsilon_i] = 0$ and $\text{Var}(e_i - \theta_0\epsilon_i) = \sigma_{Yi}^2 + \theta_0^2\sigma_{Xi}^2 - 2\rho\theta_0\sigma_{Xi}\sigma_{Yi}$, the first term on the right hand side ($\sum_{i=1}^m \psi_{1i}$) is distributed as $\mathcal{N}(0, V^*)$ and $V^* = \Theta(N||\mathbf{b}_X||_2^2)$. The second term is negligible compared to the first term since it has variance $O(m)$. Therefore, $(1/\sqrt{V^*})\psi(\theta_0) \to \mathcal{N}(0,1)$.

We next show $(-1/V^*)\psi'(\theta_0) \xrightarrow{p} 1$.

Since $\mathbb{E}[-\psi'(\theta_0)] = V^*$ by definition, it suffices to show $\text{Var}(\phi'(\theta_0)/V^*) \to 0$. With the observation that the $i-$th summand in Eq (5), $\psi_i(\theta)$, is a homogeneous quadratic polynomial of $(\tilde{b}_{Xi}, \tilde{e}_i, \tilde{\epsilon}_i) = (\sqrt{N}b_{Xi}, \sqrt{N}e_i, \sqrt{N}\epsilon_i)$:

$$\psi_i(\theta) = \frac{A_i}{B_i},$$

where

$$A_i = \Big\{((\theta_0 - \theta)\tilde{b}_{Xi} + \tilde{e}_i - \theta\tilde{\epsilon}_i)\cdot\big[(N\sigma_{Yi}^2 + N\theta_0\theta\sigma_{Xi}^2 - N\rho(\theta+\theta_0)\sigma_{Xi}\sigma_{Yi})\tilde{b}_{Xi}$$
$$+ (N\sigma_{Yi}^2 - N\rho\sigma_{Xi}\sigma_{Yi}\theta)\tilde{\epsilon}_i + (N\sigma_{Xi}^2\theta - N\rho\sigma_{Xi}\sigma_{Yi})\tilde{e}_i\big]\Big\},$$
$$B_i = (N\sigma_{Yi}^2 + N\theta^2\sigma_{Xi}^2 - 2N\rho\theta\sigma_{Xi}\sigma_{Yi})^2,$$

it is easy to see that $\psi_i'(\theta)$ is also a homogeneous quadratic polynomial of $(\tilde{b}_{Xi}, \tilde{e}_i, \tilde{\epsilon}_i)$. Also note that $\tilde{b}_{Xi}$ is treated as fixed and other terms such as $\text{Var}(\tilde{e}_i)$, $\text{Var}(\tilde{\epsilon}_i)$, $\text{Var}(\tilde{e}_i\tilde{\epsilon}_i)$, $\text{Cov}(\tilde{e}_i, \tilde{\epsilon}_i)$ are all $O(1)$, we have $\text{Var}(\psi'(\theta_0)) = O(N||\mathbf{b}_X||_2^2) \ll V^{*2} = \Theta(N^2||\mathbf{b}_X||^4)$.

Since $\psi_i'(\theta)$ is a homogeneous quadratic polynomial of $(\tilde{b}_{Xi}, \tilde{e}_i, \tilde{\epsilon}_i)$, so is $\psi_i''(\theta)$, and we can apply the same argument for $\psi'(\theta_0)$ above to $\psi''(\theta)$ and obtain that for a neighborhood $\mathcal{N}$

of θ_0, $\sup_{\theta \in \mathcal{N}} (1/V^*)\psi''(\theta) = O_p(1)$.

Lastly by Slutsky's Theorem, we have $\sqrt{V^*}(\hat{\theta} - \theta_0) \xrightarrow{d} \mathcal{N}(0, 1)$. □

With Lemmas 1-3, we can conclude Theorem 1.

So far, coupled with the selection consistency, we have established the estimation consistency and asymptotic normality of the cMLE $\hat{\theta}(\hat{K})$ for the new extended MR-cML-BIC-C method that accounts for sample overlap.

For the Graph-MRcML with T traits of interest, we first apply MR-cML-BIC-C on every pair of traits to estimate the total causal effect graph $\widehat{\mathbf{G}}_{tot}$, then estimate the direct causal effect graph as $\widehat{\mathbf{G}}_{dir} = \widehat{\mathbf{G}}_{tot}(\mathbf{I} + \widehat{\mathbf{G}}_{tot})^{-1}$. Now, we are going to prove Corollary 1.

Proof of Corollary 1. First, each element in $\text{vec}(\widehat{\mathbf{G}}_{tot})$ is consistent and asymptotic normal by Theorem 1. Next, since matrix inversion and matrix multiplication are continuous transformations [3], $\text{vec}(\widehat{\mathbf{G}}_{dir})$ is consistent by the Continuous Mapping Theorem [4]. For the asymptotic normality, we consider a $T \times (T-1)$ vector of the profile scores $\boldsymbol{\psi}$, where each element corresponds to the cMLE for an MR analysis on a pair of traits. Using the representation of Eq (8), we can write $\boldsymbol{\psi} = \boldsymbol{\psi}_1 + \boldsymbol{\psi}_2$, where the second term of is ignorable compared to the first term. Further denote $\mathbf{E} = (e_{it})$, where $e_{it} = \hat{\beta}_{it} - b_{it}$, $\hat{\beta}_{it}$ is the GWAS estimated effect of the i-th SNP on the t-th trait, and $\mathbb{E}[\hat{\beta}_{it}] = b_{it}$. Now, with Assumption 4, $\boldsymbol{\psi}_1$ is normally distributed. And we can follow the same proofs for Lemmas 1 to 3 to conclude that $\text{vec}(\widehat{\mathbf{G}}_{tot})$ is jointly asymptotic normal. Finally, since matrix inversion and matrix multiplication are continuous and differentiable [3], $\text{vec}(\widehat{\mathbf{G}}_{dir})$ is asymptotic normal by the Delta method [4]. □

A.3 Standard error of the causal parameter estimate

Following Section S2 in the Supplementary in [1], we can similarly show that the proposed cMLE and the MPLE in Lemma 3 asymptotically share the same variance. Suppose we've already selected the set of invalid IVs and for the simplicity of notation, we assume that the

m IVs are valid and used in the estimation and inference for $\hat{\theta}$. Then the $(m+1) \times (m+1)$ Fisher information matrix is

$$\mathcal{I} = -\begin{pmatrix} \frac{\partial^2 l}{\partial \theta^2} & \frac{\partial^2 l}{\partial \theta \partial \mathbf{b}_X'} \\ \frac{\partial^2 l}{\partial \theta \partial \mathbf{b}_X} & \frac{\partial^2 l}{\partial \mathbf{b}_X \mathbf{b}_X'} \end{pmatrix}, \tag{9}$$

where $\mathbf{b}_X = (b_{X1}, \ldots, b_{Xm})$. The second derivatives of log-likelihood are given as follows:

$$\begin{aligned} \frac{\partial^2 l}{\partial \theta^2} &= -\frac{1}{1-\rho^2} \sum_{i=1}^m \frac{b_{Xi}^2}{\sigma_{Yi}^2}, \\ \frac{\partial^2 l}{\partial \theta \partial b_{Xi}} &= -\frac{1}{1-\rho^2} (\frac{\rho \hat{\beta}_{Xi} - 2\rho b_{Xi}}{\sigma_{Xi}\sigma_{Yi}} - \frac{\hat{\beta}_{Yi} - 2\theta b_{Xi}}{\sigma_{Yi}^2}), \\ \frac{\partial^2 l}{\partial b_{Xi}^2} &= -\frac{1}{1-\rho^2} (\frac{1}{\sigma_{Xi}^2} - \frac{2\rho\theta}{\sigma_{Xi}\sigma_{Yi}} + \frac{\theta^2}{\sigma_{Yi}^2}). \end{aligned}$$

And we plug the cMLE $\hat{\theta}$ and $\hat{b}_{Xi}$ into above formulas and the variance estimator of the cMLE $\hat{\theta}$ is given as $\widehat{\mathrm{Var}(\hat{\theta})} = (\mathcal{I}^{-1})_{11}$. Using the formula for block matrix inversion, we have $\widehat{\mathrm{Var}(\hat{\theta})} = 1/\hat{V}$ and

$$\hat{V} = \frac{1}{1-\rho^2} \sum_i \left\{ \frac{\hat{b}_{Xi}^2}{\sigma_{Yi}^2} - \left(\frac{\rho \hat{\beta}_{Xi} - 2\rho \hat{b}_{Xi}}{\sigma_{Xi}\sigma_{Yi}} - \frac{\hat{\beta}_{Yi} - 2\hat{\theta}\hat{b}_{Xi}}{\sigma_{Yi}^2} \right)^2 \cdot \frac{1}{\frac{1}{\sigma_{Xi}^2} - \frac{2\rho\hat{\theta}}{\sigma_{Xi}\sigma_{Yi}} + \frac{\hat{\theta}^2}{\sigma_{Yi}^2}} \right\},$$

$$\hat{b}_{Xi} = (\hat{\beta}_{Xi}\sigma_{Yi}^2 - \rho\sigma_{Xi}\sigma_{Yi}(\hat{\beta}_{Yi} + \hat{\theta}\hat{\beta}_{Xi}) + \hat{\theta}\hat{\beta}_{Yi}\sigma_{Xi}^2)/(\sigma_{Yi}^2 - 2\rho\sigma_{Xi}\sigma_{Yi}\hat{\theta} + \hat{\theta}^2\sigma_{Xi}^2).$$

Similar to Section S2 in [1], we have asymptotic variance of the MPLE $\hat{\theta}$ in Lemma 3 as $1/\widehat{V^*}$ and

$$\widehat{V^*} = \sum_{i=1}^m \frac{\hat{\beta}_{Xi}^2\sigma_{Yi}^2 + \hat{\beta}_{Yi}^2\sigma_{Xi}^2 - 2\rho\hat{\beta}_{Xi}\hat{\beta}_{Yi}\sigma_{Xi}\sigma_{Yi}}{(\sigma_{Yi}^2 + \hat{\theta}^2\sigma_{Xi}^2 - 2\rho\hat{\theta}\sigma_{Xi}\sigma_{Yi})^2}.$$

Denote $c_i = \rho\sigma_{Xi}\sigma_{Yi}$, $d_i = \hat{\beta}_{Yi} - \hat{\theta}\hat{\beta}_{Xi}$ and $D = \sigma_{Yi}^2 + \hat{\theta}^2\sigma_{Xi}^2 - 2\rho\hat{\theta}\sigma_{Xi}\sigma_{Yi}$. Then we have

$$\widehat{V^*} = \frac{1}{1-\rho^2} \sum_{i=1}^m \frac{(\hat{\beta}_{Xi}^2\sigma_{Yi}^2 + \hat{\beta}_{Yi}^2\sigma_{Xi}^2 - 2c_i\hat{\beta}_{Xi}\hat{\beta}_{Yi}) \cdot (\sigma_{Xi}^2\sigma_{Yi}^2 - c_i^2) \cdot D}{D^3(\sigma_{Xi}^2\sigma_{Yi}^2)}, \tag{10}$$

and

$$\hat{V} - \widehat{V^*} = \frac{1}{1-\rho^2} \sum_i \Big\{ \frac{\sigma_{Xi}^2 \sigma_{Yi}^2 (2\sigma_{Xi}^2 \hat{\beta}_{Yi} (\hat{\theta}^2 \sigma_{Xi}^2 + \sigma_{Yi}^2) d_i - 4\sigma_{Xi}^2 \sigma_{Yi}^2 d_i^2)}{D^3(\sigma_{Xi}^2 \sigma_{Yi}^2)} \tag{11}$$

$$- \frac{2c_i \sigma_{Xi}^2 \sigma_{Yi}^2 (\hat{\beta}_{Xi} \sigma_{Yi}^2 d_i + 2\hat{\theta} \sigma_{Xi}^2 d_i^2 + 3\hat{\theta}^2 \hat{\beta}_{Xi} \sigma_{Xi}^2 d_i)}{D^3(\sigma_{Xi}^2 \sigma_{Yi}^2)} \tag{12}$$

$$+ \frac{2c_i^2 (2\sigma_{Xi}^2 \sigma_{Yi}^2 d_i^2 + \hat{\beta}_{Yi} \sigma_{Xi}^2 (\sigma_{Yi}^2 - \hat{\theta}^2 \sigma_{Xi}^2) d_i)}{D^3(\sigma_{Xi}^2 \sigma_{Yi}^2)} \tag{13}$$

$$+ \frac{2c_i^3 (\hat{\beta}_{Xi} \sigma_{Yi}^2 d_i + 2\hat{\theta} \sigma_{Xi}^2 d_i^2 + 3\hat{\theta}^2 \hat{\beta}_{Xi} \sigma_{Xi}^2 d_i)}{D^3(\sigma_{Xi}^2 \sigma_{Yi}^2)} \tag{14}$$

$$- \frac{4c_i^4 \hat{\beta}_{Yi} d_i}{D^3(\sigma_{Xi}^2 \sigma_{Yi}^2)} \Big\}. \tag{15}$$

Divided each part in Eqs (11) to (15) by the corresponding part in Eq (10), we obtain $\hat{V} - \widehat{V^*} = o_p(\widehat{V^*})$, since c_i, σ_{Xi}^2 and σ_{Yi}^2 are all $O_p(1/N)$, and $d_i = o_p(1)$ by the consistency of $\hat{\theta}$. Thus, our cMLE and the MPLE asymptotically share the same variance. This is also confirmed in numerical results as shown in the second and third columns in Tables Da, Db, Ea, Eb, H and I.

A.4 Asymptotic properties of MR-cML-BIC-I in the presence of sample overlap

A.4.1 Selection consistency

It is noted that the selection consistency of MR-cML-BIC-I provided in [1] would still hold despite the correlation between the two GWAS summary data. This can be seen from the proof in [1] that most of the arguments still hold except for some distributional arguments. For example, $\frac{\hat{\beta}_{Yi} - \tilde{\theta} \cdot \hat{\beta}_{Xi}}{\sqrt{\sigma_{Yi}^2 + \tilde{\theta}^2 \sigma_{Xi}^2}}$ is no longer $\mathcal{N}(\frac{\theta \cdot b_{Xi} + r_i - \tilde{\theta} \cdot b_{Xi}}{\sqrt{\sigma_{Yi}^2 + \tilde{\theta}^2 \sigma_{Xi}^2}}, 1)$, but we can multiply it by a strictly positive scalar $s_i = \frac{\sqrt{\sigma_{Yi}^2 + \tilde{\theta}^2 \sigma_{Xi}^2}}{\sqrt{\sigma_{Yi}^2 + \tilde{\theta}^2 \sigma_{Xi}^2 - 2\rho \tilde{\theta} \sigma_{Xi} \sigma_{Yi}}}$. Notice that $s_i = O_p(1)$ and all the inequalities still hold asymptotically. Hence MR-cML-BIC-I, which use the mis-specified likelihood with $\rho = 0$ in the presence of sample overlap, is still able to select the correct set of invalid IVs

asymptotically. We perform simulations to confirm this.

To avoid generating a large number of individual data, we simulate the GWAS summary statistics directly as follows:

$$b_{Xi} = \gamma_i + \beta_{XU}\phi_i, \tag{16}$$

$$b_{Yi} = \theta b_{Xi} + \beta_{YU}\phi_i + \alpha_i, \tag{17}$$

$$\begin{pmatrix} \hat{\beta}_{Xi} \\ \hat{\beta}_{Yi} \end{pmatrix} \sim \mathcal{N}\left(\begin{pmatrix} b_{Xi} \\ b_{Yi} \end{pmatrix}, \begin{pmatrix} \sigma_{Xi}^2 & \rho\sigma_{Xi}\sigma_{Yi} \\ \rho\sigma_{Xi}\sigma_{Yi} & \sigma_{Yi}^2 \end{pmatrix}\right) iid, \quad i = 1, \ldots, m. \tag{18}$$

We set $\gamma_i = 0.08$, $\beta_{XU} = \beta_{YU} = 1$, $\theta = 0.2$, $m = 100$. We considered 30% invalid IVs with the direct effect α_i iid from $\mathcal{N}(0.04, 0.05^2)$, and $\phi_i = 0$ (uncorrelated pleiotropy), or ϕ_i iid from Unif(0,0.01) (correlated pleiotropy). σ_{Xi} and σ_{Yi} were set to be $1/\sqrt{N}$, where the sample size $N_1 = N_2 = N$ varied from {25 000,100 000,500 000,1 000 000,5 000 000}. ρ was set to be 0 or 0.8, and we used the true ρ in MR-cML-BIC-C. We ran 10 000 replications for each scenario and calculated the average true positive rate of correctly identifying invalid IVs, and the average accuracy of classifying IVs. True positive rate is calculated as #correctly identified invalid IVs/#invalid IVs. Accuracy is calculated as (#correctly identified invalid IVs + #correctly identified valid IVs)/#IVs.

First, when $\rho = 0$, MR-cML-BIC-C and MR-cML-BIC-I were the same and gave consistent estimate and selection as shown in the left column in Figs A to C. On the other hand, when $\rho = 0.8$, MR-cML-BIC-I gave biased estimate when the sample size was not large enough, while MR-cML-BIC-C was almost unbiased. It is also noted that MR-cML-BIC-C performed better than MR-cML-BIC-I in selecting invalid IVs, though the probabilities of selecting the correct set of invalid IVs for both methods were going to 1 as the sample size increased as shown in the right column in Figs B and C.

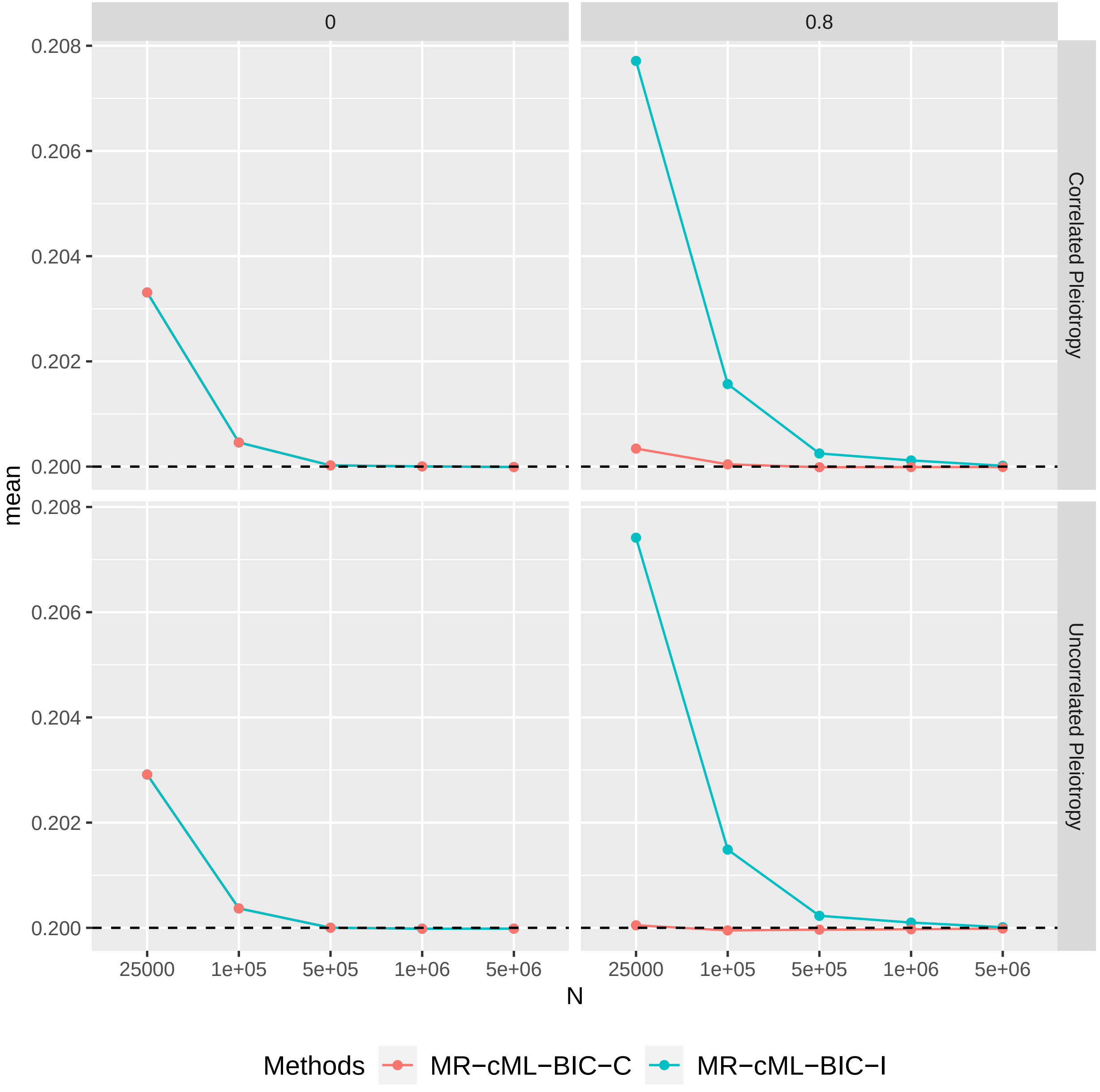

Fig A. The y-axis is the mean of $\hat{\theta}$ among 10 000 replications, and the x-axis represents different sample sizes. Dashed line is the true $\theta = 0.2$. Top: correlated pleiotropy. Bottom: uncorrelated pleiotropy. Left: $\rho = 0$. Right: $\rho = 0.8$

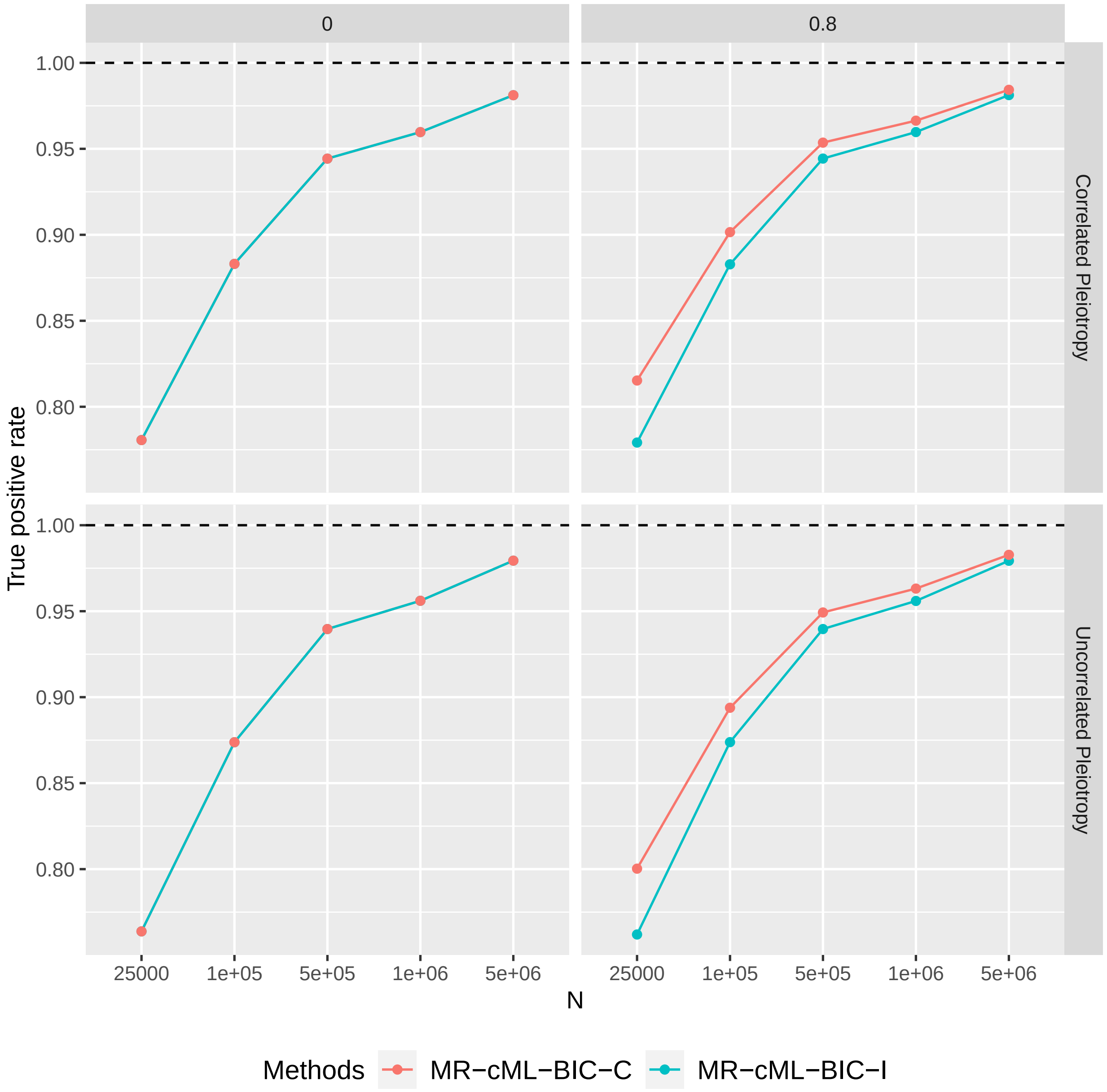

Fig B. The y-axis is the mean of true positive rates among 10 000 replications, and the x-axis represents different sample sizes. Top: correlated pleiotropy. Bottom: uncorrelated pleiotropy. Left: $\rho = 0$. Right: $\rho = 0.8$.

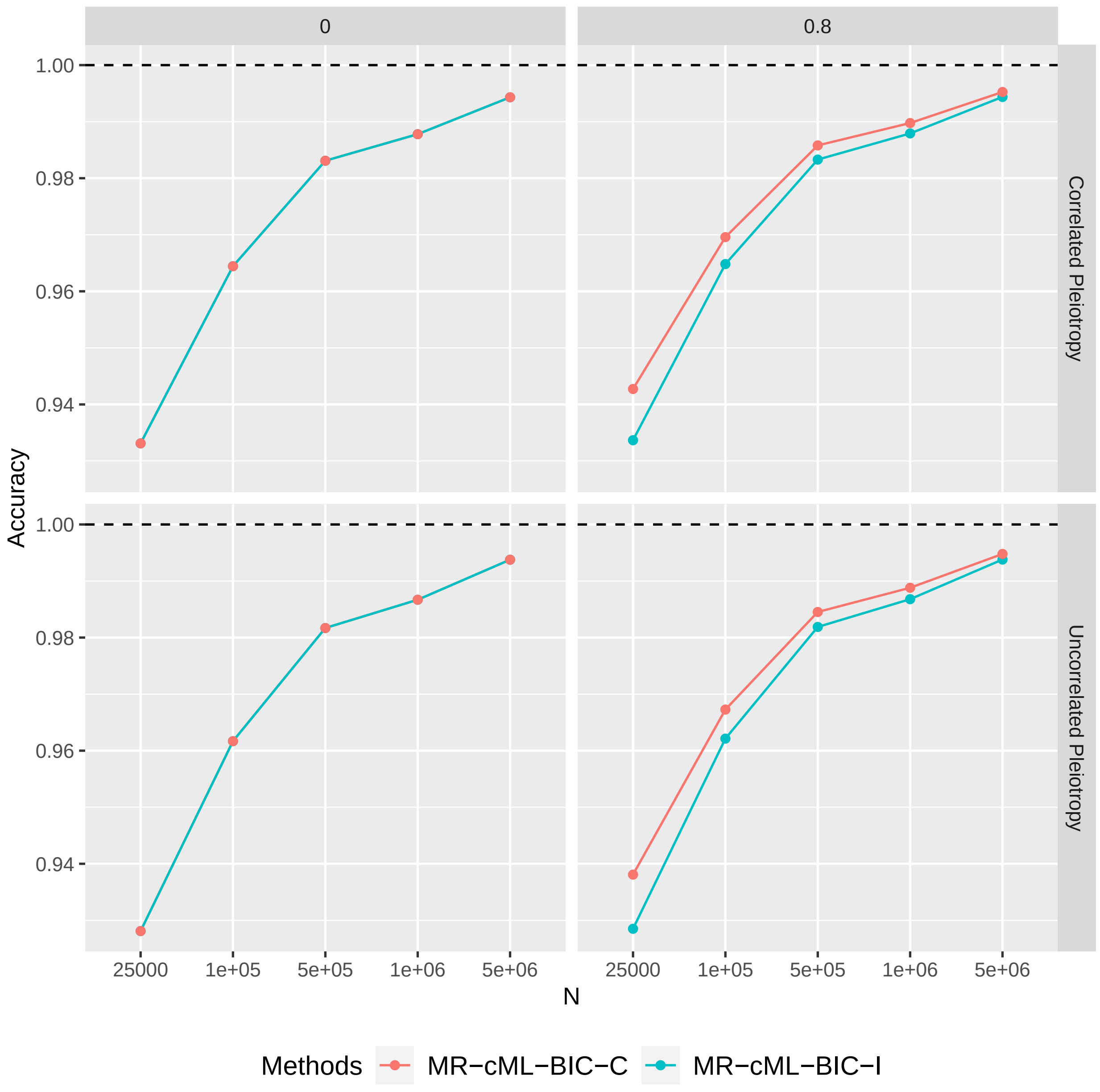

Fig C. The y-axis is the mean of accuracy among 10 000 replications, and the x-axis represents different sample sizes. Top: correlated pleiotropy. Bottom: uncorrelated pleiotropy. Left: $\rho = 0$. Right: $\rho = 0.8$.

A.4.2 Estimation consistency and a robust variance estimator

Using the representation in Eq (8), we can see that even though we ignore the correlation between $\hat{\beta}_{Xi}$ and $\hat{\beta}_{Yi}$ (i.e. $\rho = 0$) and mis-specify the (profile) likelihood, we still have $\psi(\theta_0) = o_p(\sqrt{V^*})$. Thus, the MLE under the mis-specified profile log-likelihood in [2] is

still consistent. But the usual (naive or model-based) variance estimator used in [1, 2] is not. Instead, we could use the sandwich variance estimator that is robust to model misspecification. The asymptotic normality of $\hat{\theta}_I$ in Theorem 2 can be similarly derived as in Lemma 3 with the following representation from Taylor expansion:

$$\sqrt{V_r}(\hat{\theta}_I - \theta_0) = \frac{-\psi(\theta_0; \rho = 0)/\sqrt{\mathbf{B}}}{\psi'(\theta_0; \rho = 0)/\mathbf{A} + (1/2)\psi''(\tilde{\theta}; \rho = 0)(\hat{\theta}_I - \theta_0)/\mathbf{A}}, \tag{19}$$

where $V_r = \mathbf{A}\mathbf{B}^{-1}\mathbf{A}, \mathbf{B} = \mathbb{E}[\psi^2(\theta_0; \rho = 0)], \mathbf{A} = \mathbb{E}[\psi'(\theta_0; \rho = 0)]$.

The 'bread' can be estimated empirically as

$$\begin{aligned} \widehat{\mathbf{A}} = \widehat{\mathbb{E}[\psi'(\theta_0)]} &= \sum_{i=1}^{m} \psi_i'(\hat{\theta}; \rho = 0) \\ &= \Big\{ (\hat{\beta}_{Yi}^2 \sigma_{Xi}^2 - \hat{\beta}_{Xi}^2 \sigma_{Yi}^2 - 2\hat{\theta}\hat{\beta}_{Xi}\hat{\beta}_{Yi}\sigma_{Xi}^2)(\sigma_{Yi}^2 + \hat{\theta}^2\sigma_{Xi}^2)^2 - \\ &\quad 2(\sigma_{Yi}^2 + \hat{\theta}^2\sigma_{Xi}^2)(2\hat{\theta}\sigma_{Xi}^2)(\hat{\beta}_{Yi} - \hat{\theta}\hat{\beta}_{Xi})(\hat{\beta}_{Xi}\sigma_{Yi}^2 + \hat{\beta}_{Yi}\sigma_{Xi}^2) \Big\} / (\sigma_{Yi}^2 + \hat{\theta}^2\sigma_{Xi}^2)^4. \end{aligned} \tag{20}$$

And the 'meat' can be estimated empirically as

$$\begin{aligned} \widehat{\mathbf{B}} = \widehat{\mathbb{E}[\psi^2(\theta_0)]} &= \sum_{i=1}^{m} \psi_i^2(\hat{\theta}; \rho = 0) \\ &= \sum_{i=1}^{m} \left(\frac{(\hat{\beta}_{Yi} - \hat{\theta}\hat{\beta}_{Xi})(\hat{\beta}_{Xi}\sigma_{Yi}^2 + \hat{\beta}_{Yi}\sigma_{Xi}^2\hat{\theta})}{(\sigma_{Yi}^2 + \hat{\theta}^2\sigma_{Xi}^2)^2} \right)^2. \end{aligned} \tag{21}$$

Then we have the robust variance estimator $\hat{V}_{robust} = \widehat{\mathbf{A}}^{-1}\widehat{\mathbf{B}}(\widehat{\mathbf{A}}^{-1})^T$.

We further perform simulations to confirm this. We followed the similar simulation setup as described in Section A.4.1 but under the ideal scenario with no invalid IV. We applied MR-cML-BIC-I and MR-cML-BIC-C with the oracle $K = 0$ (i.e. there is no model selection process and all IVs were valid), called **cML-BIC-I-K0** and **cML-BIC-C-K0** respectively. For MR-cML-BIC-I, we also calculated the robust variance estimate, referred to **cML-BIC-I-K0-robust**. We ran 10 000 replications for each scenario and compared the mean of the estimated standard errors (mean($SE(\hat{\theta})$)) and the empirical standard deviation of $\hat{\theta}$ ($SD(\hat{\theta})$)

from the 10 000 replications.

Fig D shows the simulation results under different scenarios for different methods. First, as shown in the first column in Fig D, when there was no sample overlap ($\rho = 0$), cML-BIC-I-K0 and cML-BIC-C-K0 performed similarly with unbiased estimates. When $\rho = 0.8$, cML-BIC-C-K0 (top) yielded unbiased estimates, while cML-BIC-I-K0 (middle) yielded biased estimates, but the bias was getting smaller when the sample size increased. This confirms that cML-BIC-I-K0 is still consistent. As for the variance estimate, we can see that when there was no sample overlap (first column), the two error bars representing $SD(\hat{\theta})$ and mean($SE(\hat{\theta})$) aligned with each other as the sample size increased for all methods. However, when $\rho = 0.8$, the two error bars aligned for cML-BIC-C-K0 and cML-BIC-I-K0-robust, but not for cML-BIC-I-K0. We can see that in the middle row, there were discrepancies between the two error bars even when the sample size kept increasing. On the other hand, cML-BIC-I-K0-robust (bottom) still gave correct variance estimates when we used the robust sandwich variance estimator.

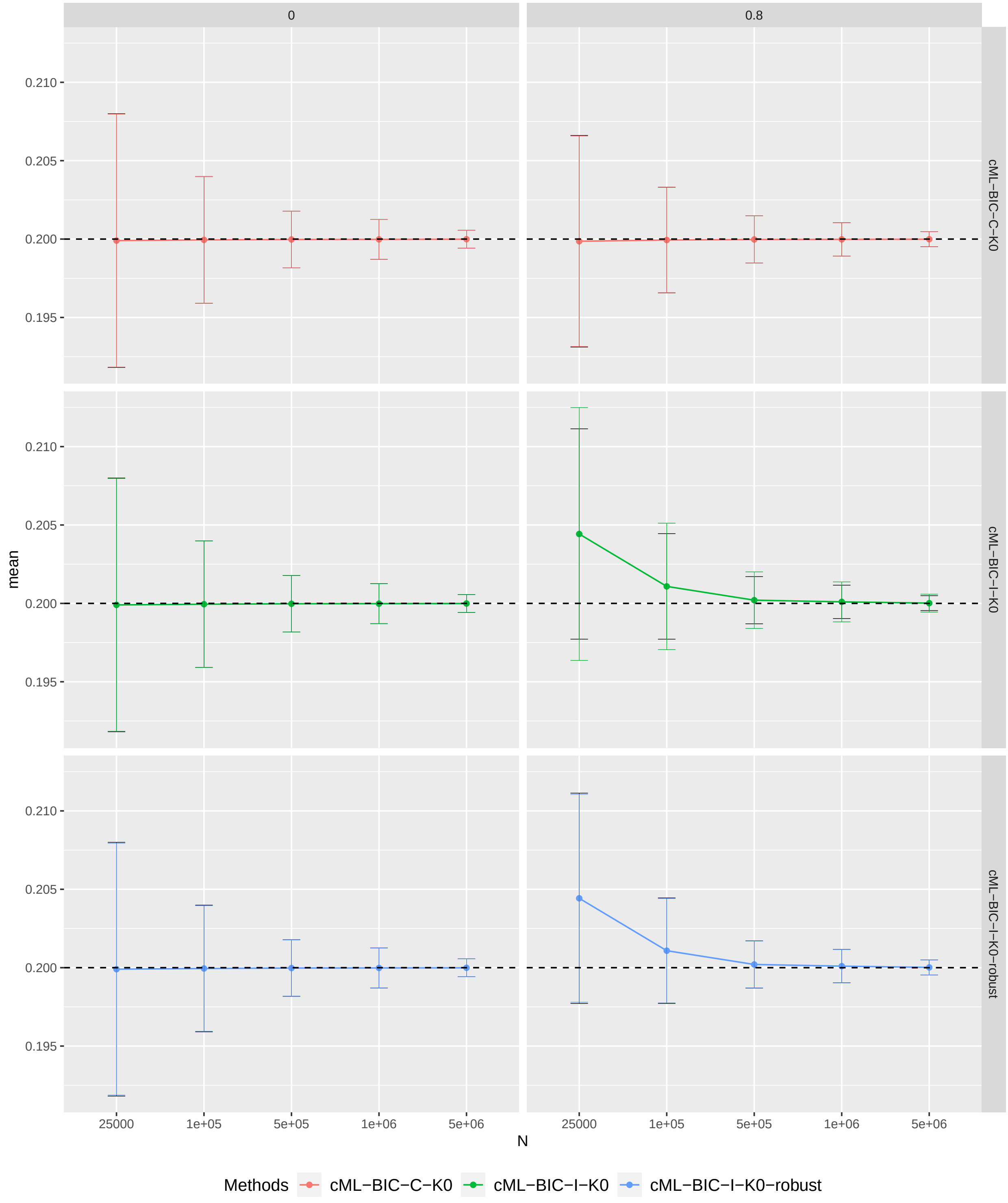

Fig D. The y-axis is the mean of $\hat{\theta}$ among 10 000 replications, and the x-axis represents different sample sizes. Dashed line is the true $\theta = 0.2$. The black error bar is $SD(\hat{\theta})$ and the colored error bar is mean($SE(\hat{\theta})$). Top row: cML-BIC-C-K0. Middle row: cML-BIC-I-K0. Bottom row: cML-BIC-I-K0-robust. Left: $\rho = 0$. Right: $\rho = 0.8$

Fig E shows the empirical coverage rates for different methods. In the first column with $\rho = 0$, the red line (cML-BIC-C-K0) and the green line (cML-BIC-I-K0) coincided with each other, yielding correct coverage rates. When $\rho = 0.8$, only cML-BIC-C-K0 gave correct coverage rates close to 95%. When the sample size was small ($N = 25\,000$), cML-BIC-I-K0 seemed to yield correct coverage rates close to 95%. But this is because it yielded biased estimates and at the same time inflated variances as shown in Fig D. As the sample size increased, cML-BIC-I-K0 yielded more conservative coverage rates. This is because it became less biased but the naive variance estimator over-estimated the true variance as shown in Fig D. It is also noted that using the robust variance estimator, cML-BIC-I-K0-robust yielded anti-conservative empirical coverage rates (and always more anti-conservative than cML-BIC-I-K0), but it was getting closer to the nominal 95% as the sample size increased. This is because although it gave correct variance estimates, the estimates of θ was still biased unless the sample size was large enough (Fig D).

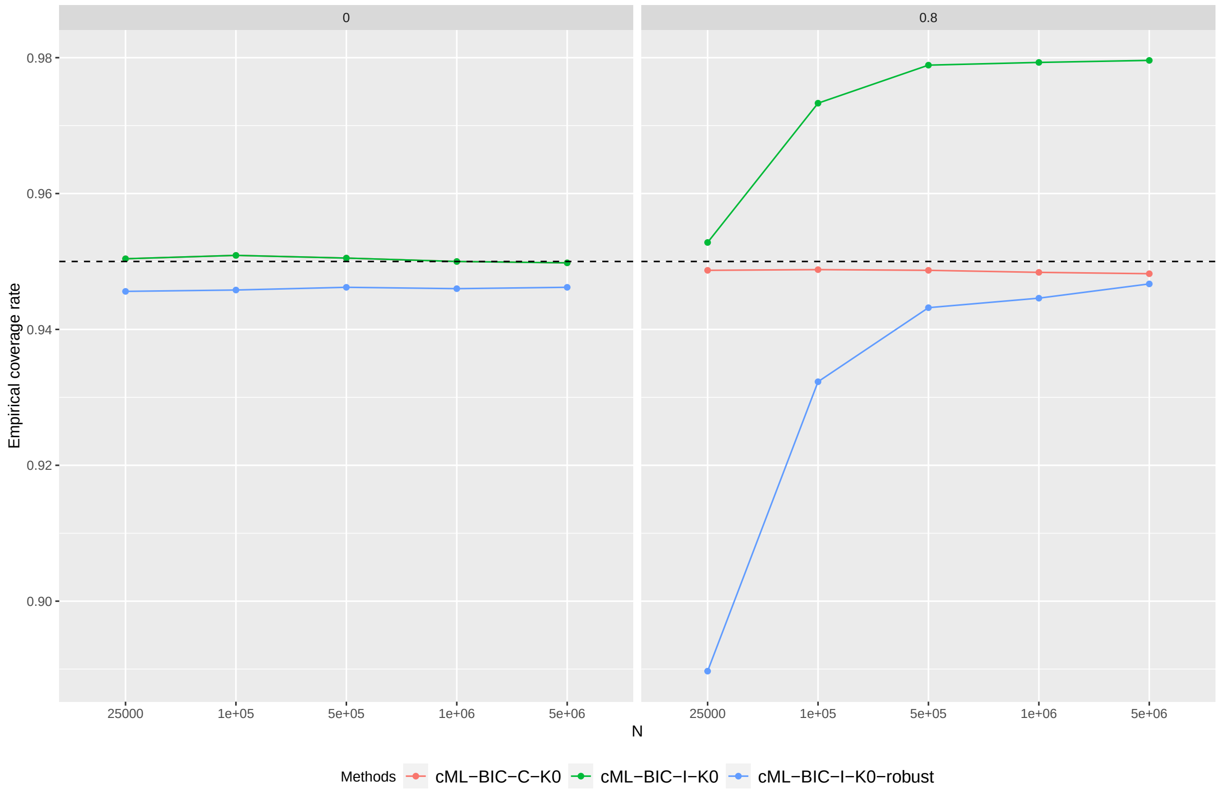

Fig E. The y-axis is the empirical coverage rate among 10 000 replications, and the x-axis represents different sample sizes. Dashed line is the nominal level 95%. Left: $\rho = 0$. Right: $\rho = 0.8$.

B More simulation results for MR methods with sample overlap

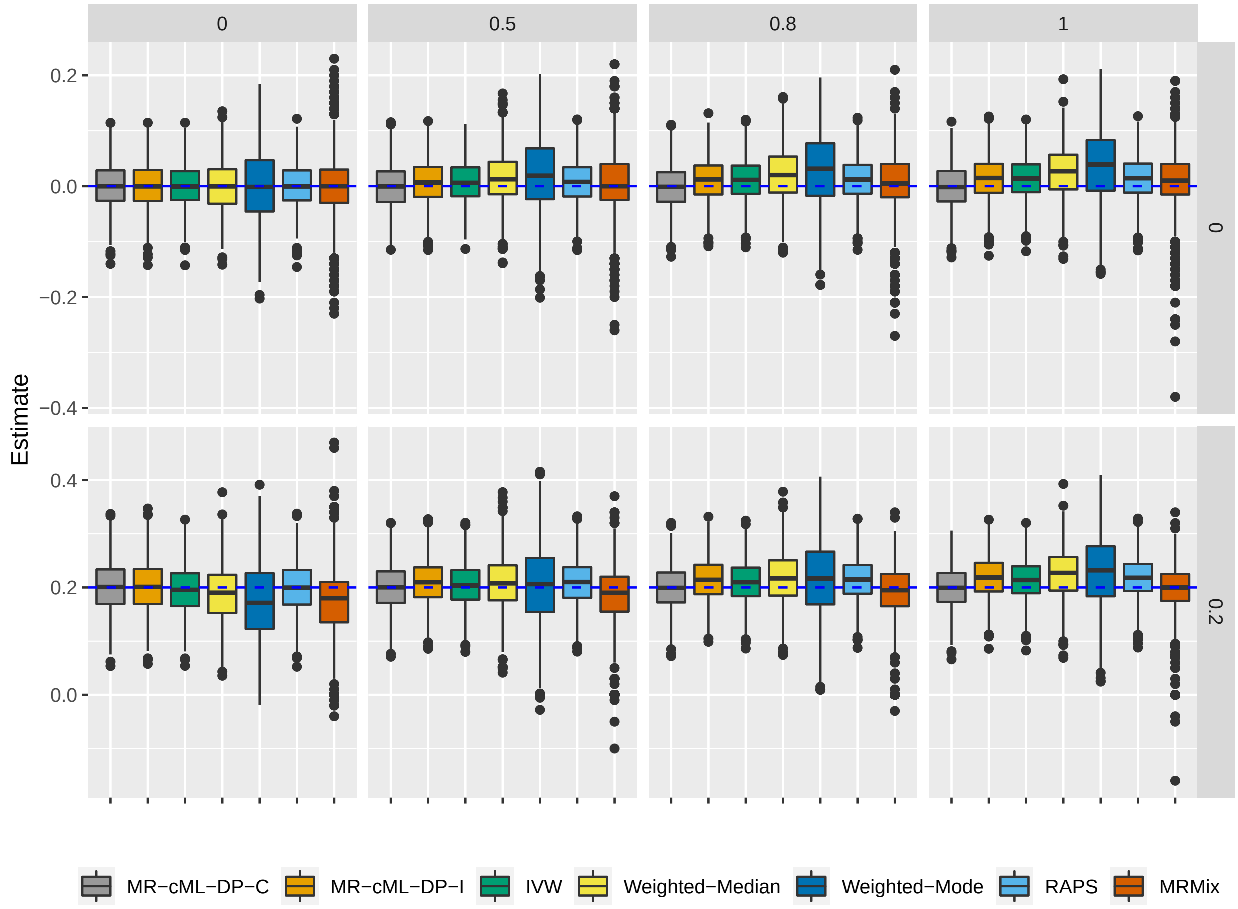

Fig F. Estimates of the causal effect θ with 0% invalid IVs across 1000 replicates. From left to right correspond to 0%, 50%, 80% and 100% overlapping samples. Top panel: $\theta = 0$ and bottom panel: $\theta = 0.2$.

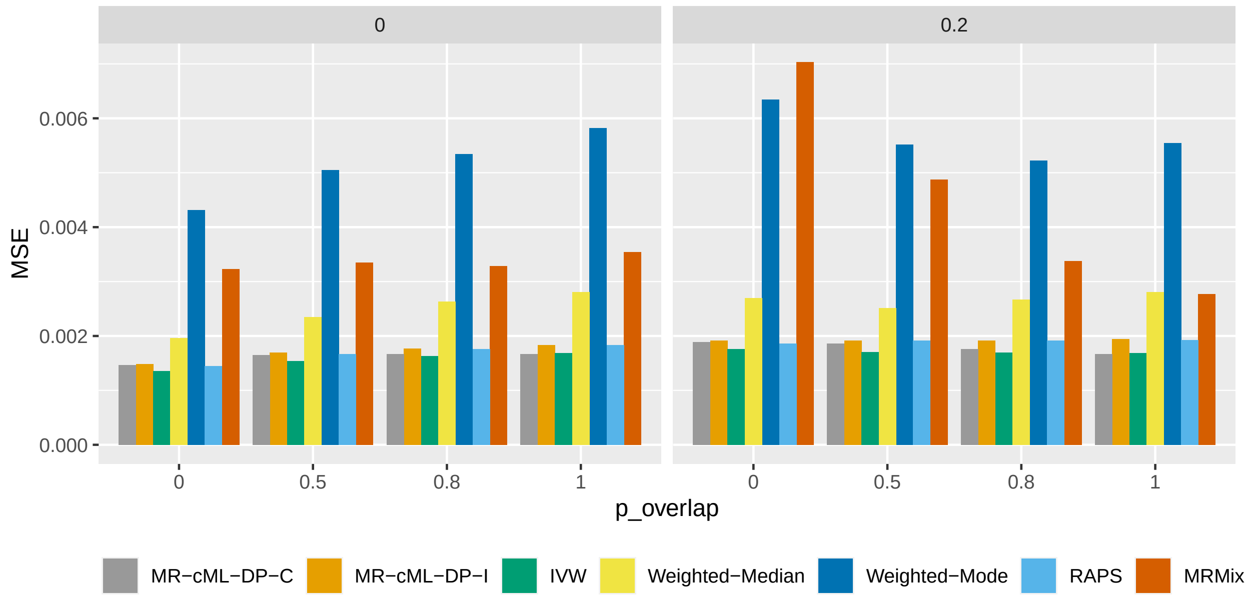

Fig G. Mean squared error (MSE) in the presence of 0% invalid IVs . X-axis represents different proportions of sample overlap (0%, 50%, 80% and 100%). Left panel: $\theta = 0$ and right panel: $\theta = 0.2$.

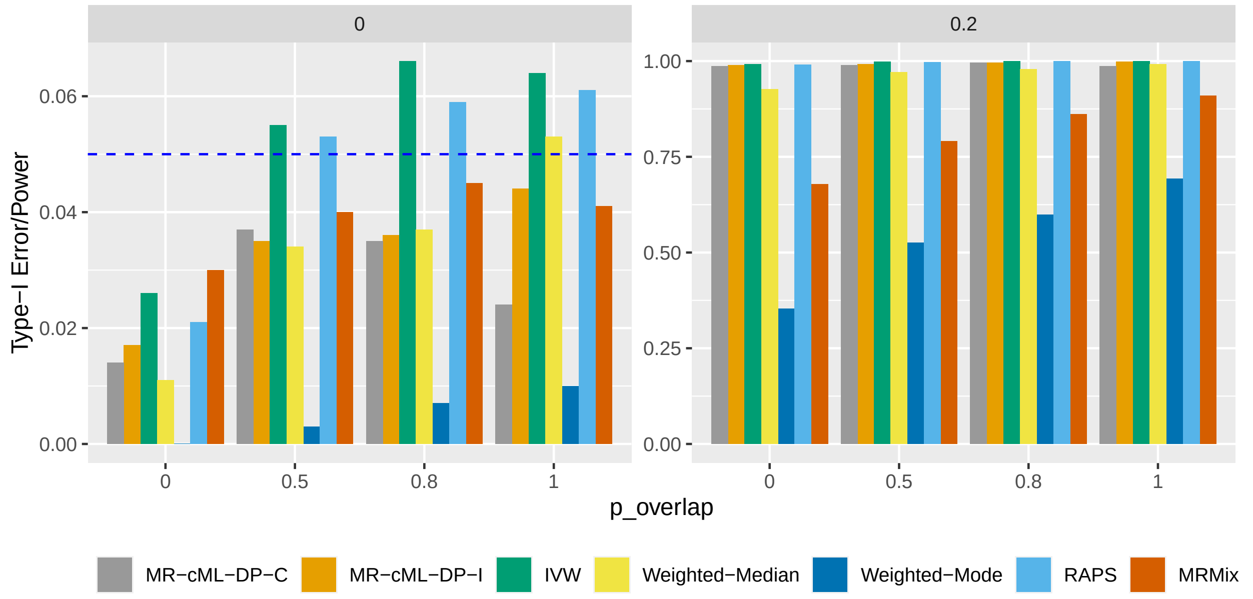

Fig H. Empirical type-I error and power in the presence of 0% invalid IVs . X-axis represents different proportions of sample overlap (0%, 50%, 80% and 100%). Left: $\theta = 0$ (type-I error) and right: $\theta = 0.2$ (power).

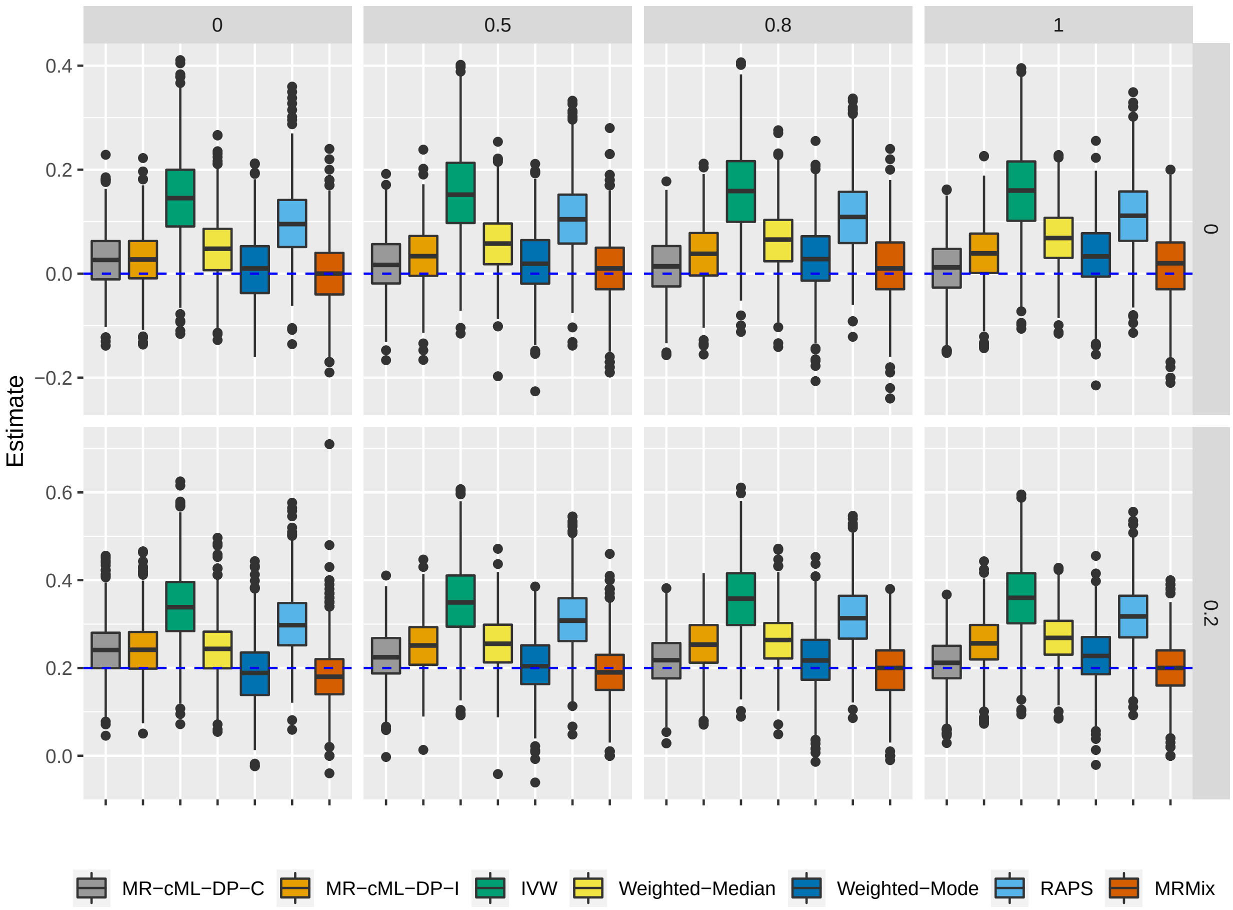

Fig I. Estimates of the causal effect θ with 30% invalid IVs (uncorrelated pleiotropy) across 1000 replicates. From left to right correspond to 0%, 50%, 80% and 100% overlapping samples. Top panel: $\theta = 0$ and bottom panel: $\theta = 0.2$.

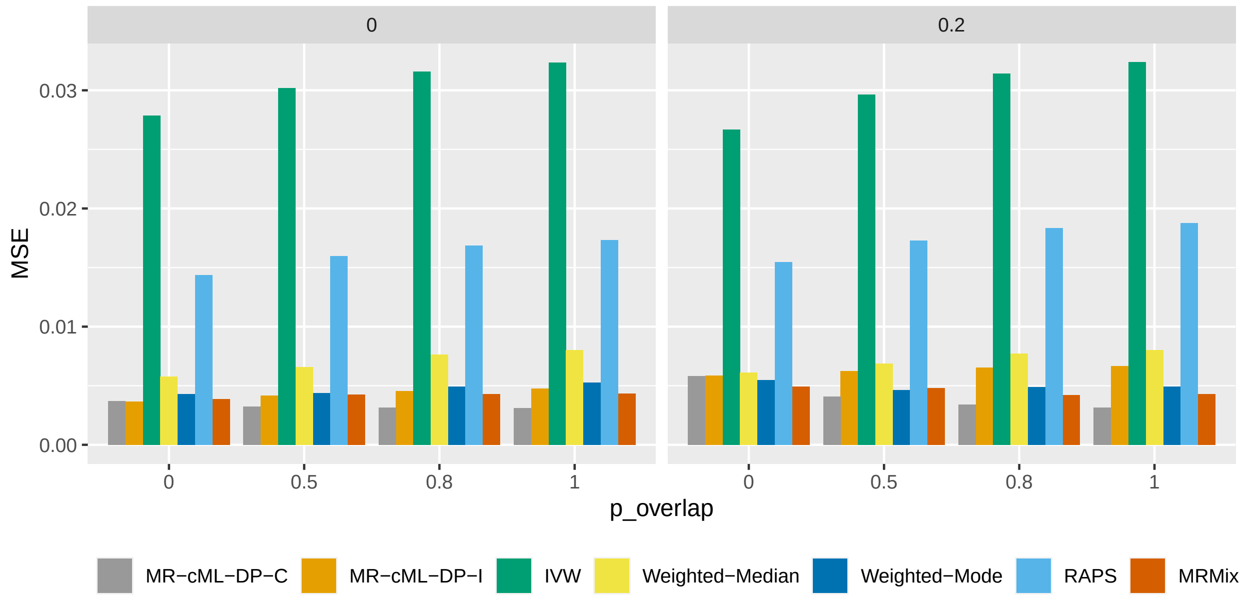

Fig J. Mean squared error (MSE) in the presence of 30% invalid IVs (uncorrelated pleiotropy). X-axis represents different proportions of sample overlap (0%, 50%, 80% and 100%). Left panel: $\theta = 0$ and right panel: $\theta = 0.2$.

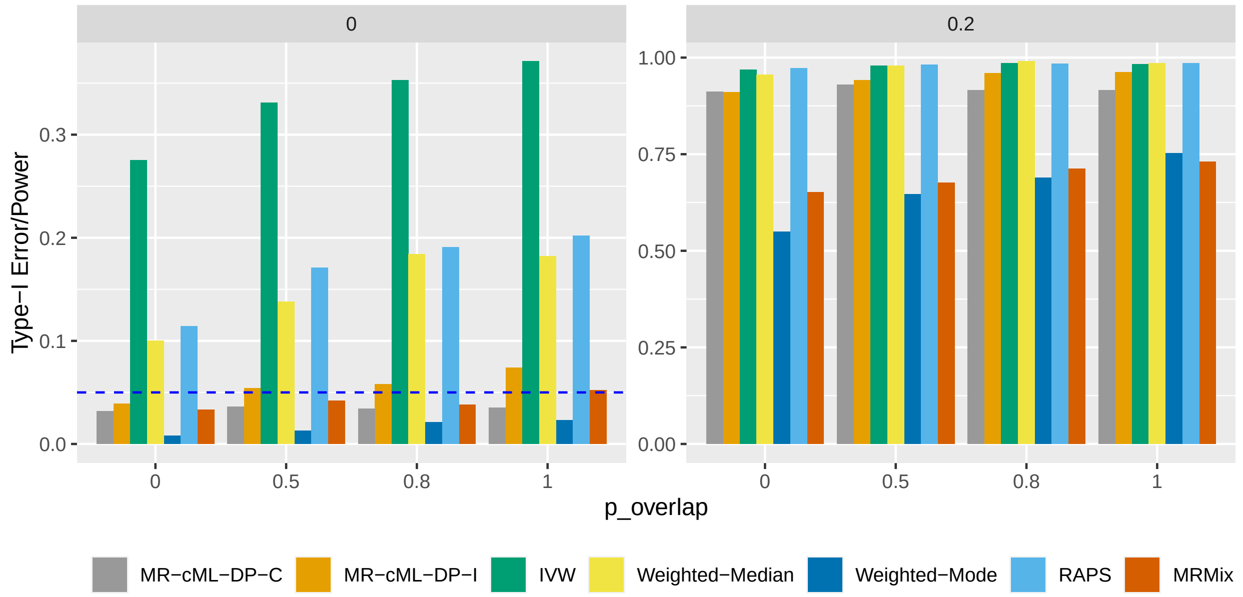

Fig K. Empirical type-I error and power in the presence of 30% invalid IVs with uncorrelated pleiotropy. X-axis represents different proportions of sample overlap (0%, 50%, 80% and 100%). Left: $\theta = 0$ (type-I error) and right: $\theta = 0.2$ (power).

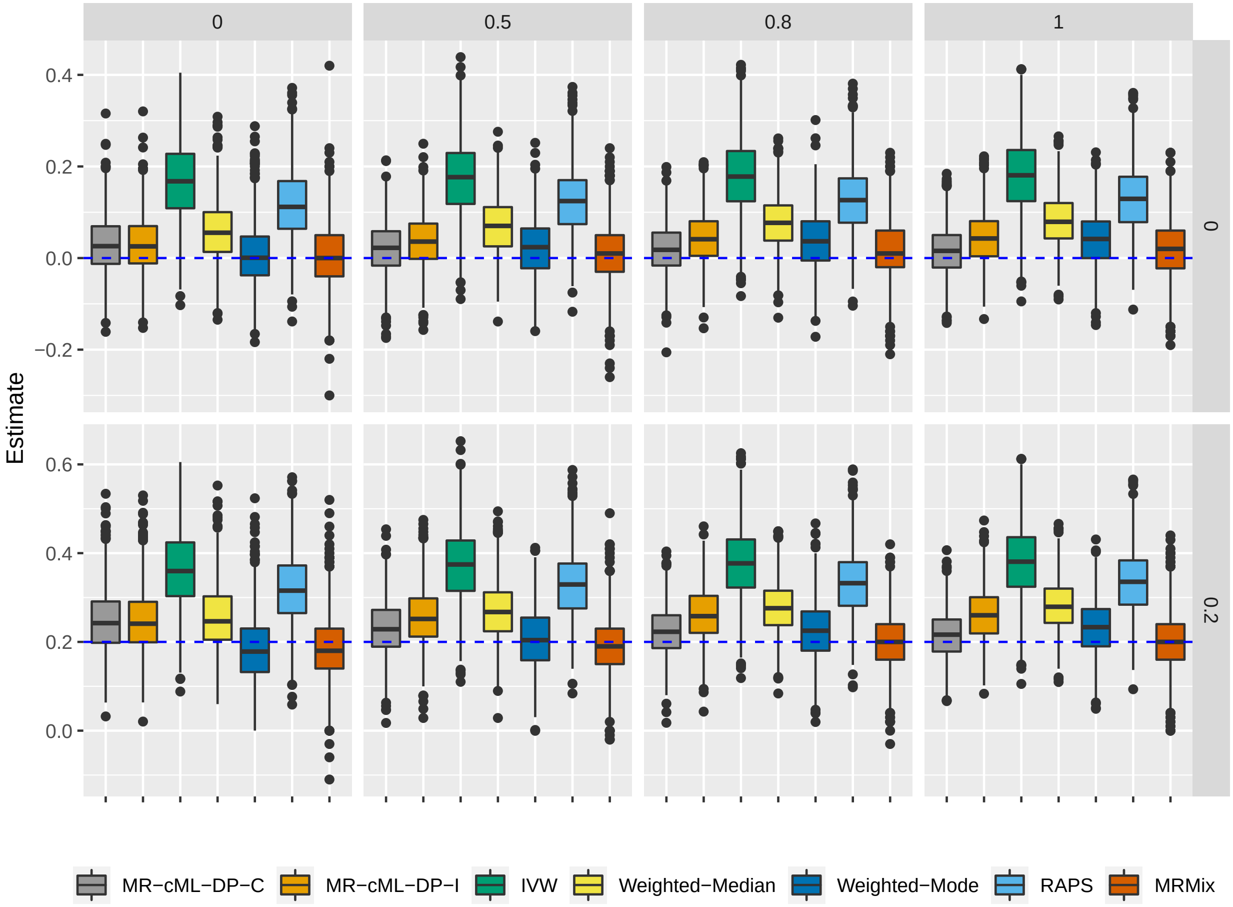

Fig L. Estimates of the causal effect θ with 30% invalid IVs (correlated pleiotropy) across 1000 replicates. From left to right correspond to 0%, 50%, 80% and 100% overlapping samples. Top panel: $\theta = 0$ and bottom panel: $\theta = 0.2$.

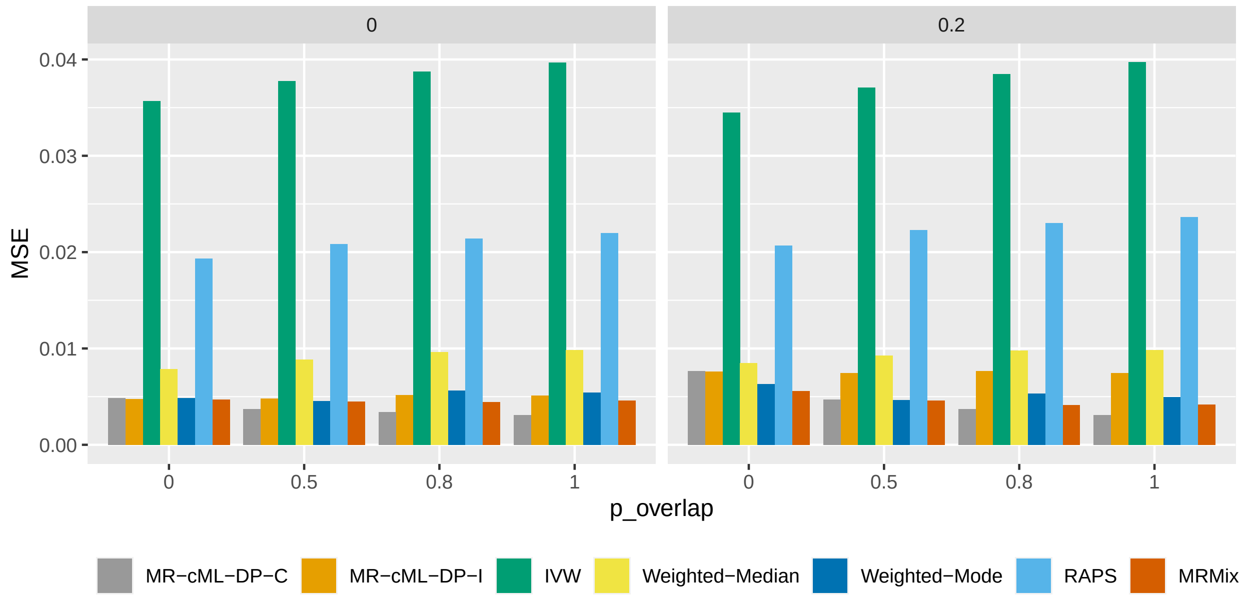

Fig M. Mean squared error (MSE) in the presence of 30% invalid IVs (correlated pleiotropy). X-axis represents different proportions of sample overlap (0%, 50%, 80% and 100%). Left panel: $\theta = 0$ and right panel: $\theta = 0.2$.

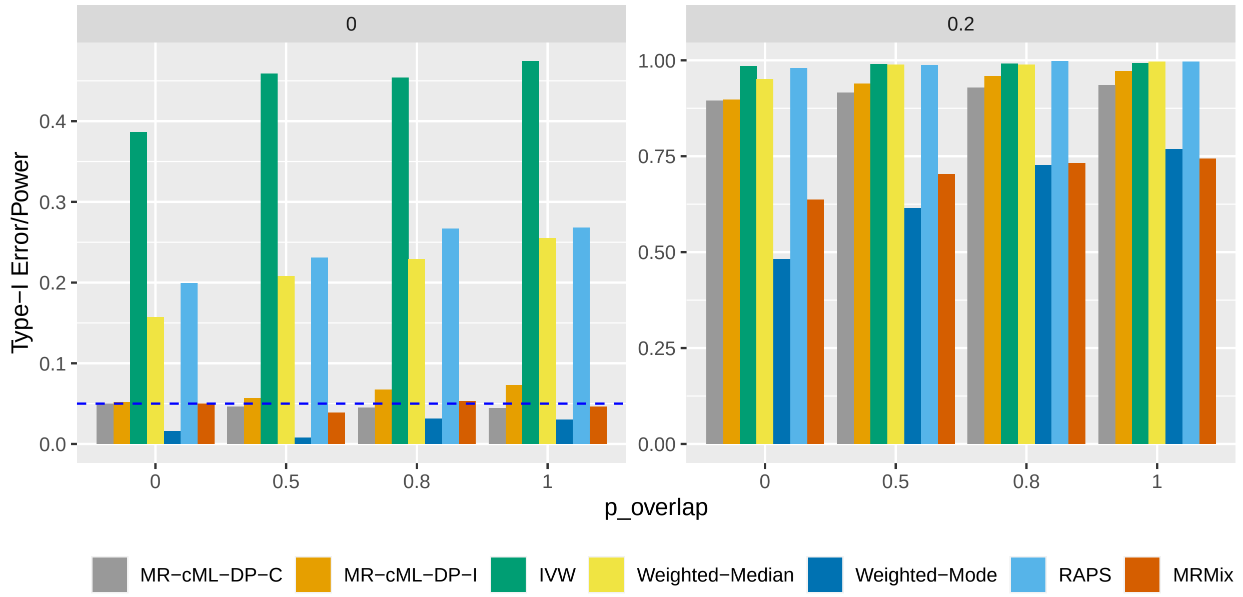

Fig N. Empirical type-I error and power in the presence of 30% invalid IVs with correlated pleiotropy. X-axis represents different proportions of sample overlap (0%, 50%, 80% and 100%). Left: $\theta = 0$ (type-I error) and right: $\theta = 0.2$ (power).

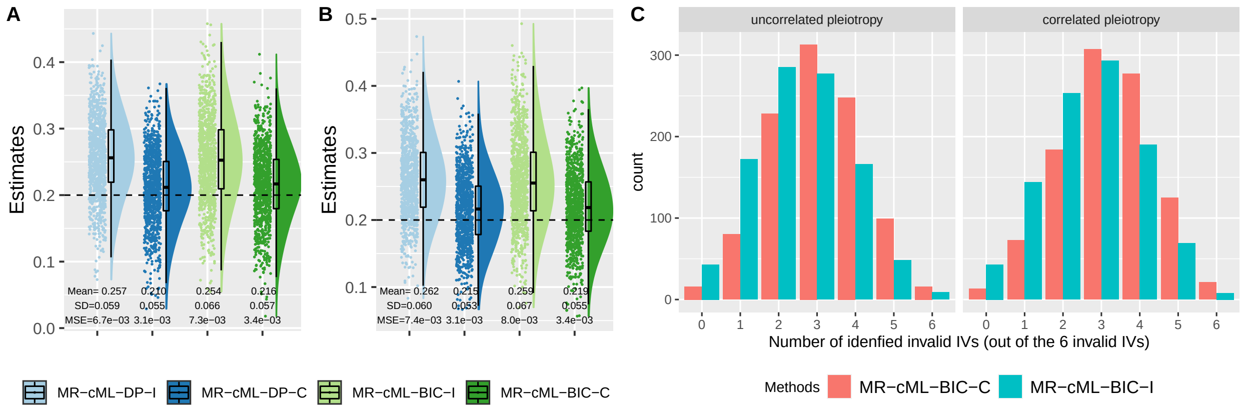

Fig O. Simulation results with 30% invalid IVs and 100% sample overlap. Panel A: empirical distributions of the estimates of the causal effect $\theta = 0.2$ with uncorrelated pleiotropy. Panel B: empirical distributions of the estimates of the causal effect $\theta = 0.2$ with correlated pleiotropy. Panel C: empirical frequency distributions of the numbers of identified invalid IVs (out of a total of 6) by BIC from 1000 replications (left: with uncorrelated pleiotropy; right: with correlated pleiotropy).

Fig O Panels A and B show the distributions of the causal estimates in the case of 100% sample overlap and 30% invalid IVs with uncorrelated and correlated pleiotropy respectively. We note that although all estimates were biased upward, MR-cML-C yielded much less biased estimates than MR-cML-I in both scenarios. One possible reasons is that MR-cML-C performed better in identifying invalid IVs than MR-cML-I in the presence of sample overlap, while both methods sometimes might fail to identify all invalid IVs (perhaps due to the small sample size and/or small effects of some invalid IVs). Fig O Panel C confirmed this point by showing the frequencies (from 1000 independent replications) of the numbers of the correctly identified invalid IVs (out of $20 \times 0.3 = 6$) using MR-cML-BIC-C and MR-cML-BIC-I respectively. We can clearly see that MR-cML-BIC-C tended to identify more of the true invalid IVs than MR-cML-BIC-I.

C More simulation results on the different versions of MR-cML

In this section, we omit the prefix 'MR-' in all methods for a clearer presentation. For example, MR-cML-BIC-I becomes cML-BIC-I, and MR-cML-DP-I becomes cML-DP-I.

C.1 Detailed results of simulations in the main text

Table A. In each cell, from top to bottom are empirical type-I error/power, mean($\hat{\theta}$), SD($\hat{\theta}$), mean(SE($\hat{\theta}$)), coverage rate, MSE, when $N = 25000$ and all valid IVs.

p_overlap	θ	$\hat{\rho}$	cML-BIC-I	cML-DP-I	cML-MA-I	cML-MA-DP-I	cML-BIC-C	cML-DP-C	cML-MA-C	cML-MA-DP-C
0.0	0.0	-0.001	0.034<br>0.001<br>0.038<br>0.039<br>0.966<br>0.001	0.017<br>0.000<br>0.039<br>0.047<br>0.983<br>0.001	0.033<br>0.000<br>0.038<br>0.039<br>0.967<br>0.001	0.020<br>0.000<br>0.039<br>0.046<br>0.980<br>0.001	0.036<br>0.001<br>0.038<br>0.039<br>0.964<br>0.001	0.014<br>0.000<br>0.038<br>0.047<br>0.986<br>0.001	0.036<br>0.000<br>0.038<br>0.039<br>0.964<br>0.001	0.015<br>0.000<br>0.038<br>0.046<br>0.985<br>0.001
	0.2	0.000	0.995<br>0.201<br>0.043<br>0.044<br>0.966<br>0.002	0.989<br>0.201<br>0.044<br>0.053<br>0.985<br>0.002	0.994<br>0.201<br>0.043<br>0.044<br>0.969<br>0.002	0.991<br>0.201<br>0.044<br>0.052<br>0.981<br>0.002	0.995<br>0.201<br>0.043<br>0.044<br>0.967<br>0.002	0.986<br>0.201<br>0.044<br>0.053<br>0.982<br>0.002	0.994<br>0.201<br>0.043<br>0.044<br>0.970<br>0.002	0.988<br>0.201<br>0.043<br>0.052<br>0.981<br>0.002
0.5	0.0	0.247	0.065<br>0.008<br>0.040<br>0.039<br>0.935<br>0.002	0.035<br>0.008<br>0.040<br>0.047<br>0.965<br>0.002	0.062<br>0.008<br>0.040<br>0.039<br>0.938<br>0.002	0.041<br>0.008<br>0.040<br>0.046<br>0.959<br>0.002	0.063<br>0.001<br>0.040<br>0.039<br>0.937<br>0.002	0.037<br>0.000<br>0.041<br>0.047<br>0.963<br>0.002	0.062<br>0.001<br>0.040<br>0.039<br>0.938<br>0.002	0.040<br>0.000<br>0.041<br>0.046<br>0.960<br>0.002
	0.2	0.312	0.999<br>0.210<br>0.042<br>0.044<br>0.948<br>0.002	0.992<br>0.210<br>0.043<br>0.052<br>0.970<br>0.002	0.999<br>0.210<br>0.042<br>0.044<br>0.948<br>0.002	0.996<br>0.210<br>0.043<br>0.051<br>0.969<br>0.002	0.997<br>0.201<br>0.043<br>0.041<br>0.939<br>0.002	0.989<br>0.201<br>0.043<br>0.050<br>0.972<br>0.002	0.998<br>0.201<br>0.043<br>0.042<br>0.943<br>0.002	0.990<br>0.201<br>0.043<br>0.049<br>0.967<br>0.002
0.8	0.0	0.395	0.081<br>0.011<br>0.040<br>0.039<br>0.919<br>0.002	0.036<br>0.011<br>0.040<br>0.047<br>0.964<br>0.002	0.080<br>0.011<br>0.040<br>0.039<br>0.920<br>0.002	0.042<br>0.011<br>0.040<br>0.046<br>0.958<br>0.002	0.068<br>0.000<br>0.040<br>0.039<br>0.932<br>0.002	0.035<br>-0.001<br>0.041<br>0.047<br>0.965<br>0.002	0.061<br>0.000<br>0.040<br>0.039<br>0.939<br>0.002	0.040<br>-0.001<br>0.041<br>0.046<br>0.960<br>0.002
	0.2	0.500	0.999<br>0.215<br>0.041<br>0.044<br>0.945<br>0.002	0.996<br>0.215<br>0.041<br>0.051<br>0.972<br>0.002	0.999<br>0.215<br>0.041<br>0.044<br>0.947<br>0.002	0.996<br>0.215<br>0.041<br>0.050<br>0.968<br>0.002	0.999<br>0.200<br>0.041<br>0.040<br>0.934<br>0.002	0.996<br>0.200<br>0.042<br>0.048<br>0.966<br>0.002	0.997<br>0.200<br>0.041<br>0.040<br>0.941<br>0.002	0.996<br>0.199<br>0.042<br>0.047<br>0.967<br>0.002
1.0	0.0	0.493	0.082<br>0.014<br>0.040<br>0.039<br>0.918<br>0.002	0.044<br>0.014<br>0.040<br>0.047<br>0.956<br>0.002	0.077<br>0.014<br>0.040<br>0.039<br>0.923<br>0.002	0.049<br>0.014<br>0.040<br>0.046<br>0.951<br>0.002	0.064<br>0.000<br>0.040<br>0.039<br>0.936<br>0.002	0.024<br>-0.001<br>0.041<br>0.047<br>0.976<br>0.002	0.059<br>0.000<br>0.040<br>0.039<br>0.941<br>0.002	0.031<br>-0.001<br>0.041<br>0.045<br>0.969<br>0.002
	0.2	0.625	0.999<br>0.219<br>0.040<br>0.044<br>0.935<br>0.002	0.998<br>0.219<br>0.040<br>0.050<br>0.966<br>0.002	0.999<br>0.219<br>0.039<br>0.044<br>0.940<br>0.002	0.999<br>0.219<br>0.040<br>0.049<br>0.964<br>0.002	0.999<br>0.200<br>0.040<br>0.039<br>0.935<br>0.002	0.986<br>0.199<br>0.041<br>0.047<br>0.977<br>0.002	0.999<br>0.200<br>0.040<br>0.039<br>0.941<br>0.002	0.989<br>0.199<br>0.041<br>0.045<br>0.972<br>0.002

Table B. In each cell, from top to bottom are empirical type-I error/power, mean($\hat{\theta}$), SD($\hat{\theta}$), mean(SE($\hat{\theta}$)), coverage rate, MSE, when $N = 25000$ and 30% invalid IVs with uncorrelated pleiotropy.

p_overlap	θ	$\hat{\rho}$	cML-BIC-I	cML-DP-I	cML-MA-I	cML-MA-DP-I	cML-BIC-C	cML-DP-C	cML-MA-C	cML-MA-DP-C
0.0	0.0	0.002	0.167	0.039	0.131	0.045	0.165	0.032	0.130	0.039
			0.023	0.027	0.024	0.028	0.023	0.027	0.024	0.027
			0.057	0.054	0.055	0.054	0.056	0.055	0.055	0.055
			0.042	0.066	0.046	0.063	0.042	0.066	0.046	0.063
			0.833	0.961	0.870	0.955	0.835	0.968	0.870	0.961
			0.004	0.004	0.004	0.004	0.004	0.004	0.004	0.004
	0.2	0.002	0.989	0.911	0.991	0.929	0.986	0.912	0.990	0.928
			0.236	0.243	0.237	0.243	0.234	0.242	0.237	0.243
			0.069	0.063	0.065	0.063	0.068	0.063	0.066	0.063
			0.048	0.080	0.052	0.076	0.048	0.079	0.052	0.075
			0.796	0.961	0.835	0.948	0.800	0.963	0.836	0.955
			0.006	0.006	0.006	0.006	0.006	0.006	0.006	0.006
0.5	0.0	0.248	0.205	0.054	0.169	0.070	0.169	0.036	0.138	0.047
			0.029	0.033	0.030	0.033	0.018	0.018	0.017	0.018
			0.058	0.056	0.056	0.056	0.056	0.054	0.055	0.054
			0.042	0.066	0.046	0.062	0.042	0.063	0.045	0.060
			0.795	0.946	0.831	0.930	0.831	0.964	0.862	0.953
			0.004	0.004	0.004	0.004	0.003	0.003	0.003	0.003
	0.2	0.314	0.992	0.942	0.993	0.964	0.983	0.930	0.980	0.944
			0.246	0.250	0.246	0.250	0.224	0.226	0.223	0.226
			0.068	0.061	0.064	0.061	0.062	0.058	0.060	0.058
			0.048	0.076	0.052	0.072	0.045	0.069	0.048	0.065
			0.737	0.956	0.801	0.938	0.799	0.970	0.848	0.961
			0.007	0.006	0.006	0.006	0.004	0.004	0.004	0.004
0.8	0.0	0.397	0.241	0.058	0.200	0.080	0.161	0.034	0.120	0.039
			0.035	0.036	0.034	0.037	0.016	0.013	0.013	0.013
			0.060	0.057	0.057	0.057	0.056	0.055	0.055	0.054
			0.042	0.066	0.046	0.062	0.042	0.062	0.045	0.059
			0.759	0.942	0.800	0.920	0.839	0.966	0.880	0.961
			0.005	0.005	0.004	0.005	0.003	0.003	0.003	0.003
	0.2	0.501	0.994	0.959	0.991	0.970	0.986	0.916	0.979	0.932
			0.252	0.254	0.252	0.255	0.218	0.216	0.216	0.217
			0.067	0.060	0.063	0.060	0.058	0.056	0.057	0.056
			0.048	0.074	0.052	0.071	0.043	0.064	0.046	0.061
			0.705	0.938	0.764	0.919	0.838	0.964	0.879	0.954
			0.007	0.007	0.007	0.007	0.004	0.003	0.003	0.003
1.0	0.0	0.496	0.252	0.074	0.207	0.091	0.161	0.035	0.125	0.043
			0.036	0.039	0.037	0.039	0.016	0.010	0.011	0.010
			0.060	0.057	0.058	0.057	0.056	0.055	0.056	0.055
			0.042	0.065	0.046	0.062	0.042	0.061	0.045	0.058
			0.748	0.926	0.793	0.909	0.839	0.965	0.875	0.957
			0.005	0.005	0.005	0.005	0.003	0.003	0.003	0.003
	0.2	0.626	0.992	0.962	0.994	0.970	0.984	0.916	0.967	0.932
			0.254	0.257	0.255	0.257	0.216	0.210	0.211	0.211
			0.066	0.059	0.062	0.059	0.057	0.055	0.056	0.055
			0.048	0.073	0.052	0.069	0.042	0.061	0.045	0.058
			0.708	0.921	0.761	0.897	0.834	0.959	0.873	0.952
			0.007	0.007	0.007	0.007	0.003	0.003	0.003	0.003

Table C. In each cell, from top to bottom are empirical type-I error/power, mean($\hat{\theta}$), SD($\hat{\theta}$), mean(SE($\hat{\theta}$)), coverage rate, MSE, when $N = 25000$ and 30% invalid IVs with correlated pleiotropy.

p_overlap	θ	$\hat{\rho}$	cML-BIC-I	cML-DP-I	cML-MA-I	cML-MA-DP-I	cML-BIC-C	cML-DP-C	cML-MA-C	cML-MA-DP-C
0.0	0.0	0.001	0.221	0.052	0.177	0.065	0.218	0.050	0.176	0.062
			0.026	0.030	0.027	0.031	0.026	0.030	0.027	0.031
			0.064	0.062	0.063	0.062	0.063	0.063	0.063	0.063
			0.042	0.068	0.046	0.065	0.042	0.069	0.046	0.065
			0.779	0.948	0.823	0.935	0.782	0.950	0.824	0.938
			0.005	0.005	0.005	0.005	0.005	0.005	0.005	0.005
	0.2	0.001	0.977	0.897	0.977	0.927	0.978	0.895	0.980	0.920
			0.241	0.247	0.242	0.248	0.239	0.248	0.241	0.249
			0.078	0.073	0.074	0.073	0.075	0.073	0.074	0.073
			0.047	0.082	0.052	0.077	0.047	0.082	0.052	0.077
			0.735	0.935	0.795	0.924	0.745	0.934	0.794	0.919
			0.008	0.008	0.007	0.008	0.007	0.008	0.007	0.008
0.5	0.0	0.250	0.228	0.057	0.188	0.065	0.184	0.046	0.147	0.055
			0.033	0.037	0.034	0.038	0.022	0.021	0.020	0.022
			0.063	0.059	0.060	0.059	0.060	0.057	0.058	0.057
			0.042	0.068	0.046	0.064	0.042	0.064	0.045	0.061
			0.772	0.943	0.812	0.935	0.816	0.954	0.853	0.945
			0.005	0.005	0.005	0.005	0.004	0.004	0.004	0.004
	0.2	0.315	0.988	0.939	0.986	0.962	0.982	0.915	0.977	0.937
			0.249	0.255	0.250	0.256	0.228	0.230	0.227	0.231
			0.072	0.066	0.068	0.066	0.065	0.062	0.063	0.062
			0.048	0.079	0.053	0.075	0.045	0.071	0.048	0.067
			0.728	0.928	0.783	0.916	0.785	0.960	0.828	0.949
			0.008	0.007	0.007	0.008	0.005	0.005	0.005	0.005
0.8	0.0	0.397	0.256	0.067	0.206	0.084	0.176	0.045	0.139	0.055
			0.039	0.042	0.040	0.043	0.020	0.018	0.019	0.019
			0.061	0.058	0.059	0.058	0.057	0.055	0.055	0.055
			0.042	0.067	0.046	0.063	0.042	0.062	0.045	0.059
			0.744	0.933	0.794	0.916	0.824	0.955	0.861	0.945
			0.005	0.005	0.005	0.005	0.004	0.003	0.003	0.003
	0.2	0.502	0.993	0.958	0.992	0.972	0.984	0.928	0.985	0.945
			0.257	0.261	0.258	0.261	0.223	0.222	0.221	0.223
			0.069	0.063	0.066	0.063	0.059	0.057	0.058	0.057
			0.048	0.076	0.052	0.072	0.043	0.064	0.046	0.061
			0.697	0.920	0.753	0.899	0.819	0.958	0.855	0.948
			0.008	0.008	0.008	0.008	0.004	0.004	0.004	0.004
1.0	0.0	0.496	0.259	0.073	0.222	0.090	0.151	0.044	0.111	0.052
			0.041	0.044	0.041	0.044	0.019	0.015	0.015	0.015
			0.061	0.057	0.058	0.056	0.055	0.054	0.054	0.054
			0.042	0.066	0.046	0.063	0.042	0.061	0.045	0.058
			0.741	0.927	0.778	0.910	0.849	0.956	0.889	0.948
			0.005	0.005	0.005	0.005	0.003	0.003	0.003	0.003
	0.2	0.627	0.994	0.972	0.994	0.982	0.988	0.935	0.985	0.949
			0.259	0.262	0.260	0.263	0.219	0.215	0.215	0.216
			0.067	0.060	0.063	0.059	0.055	0.053	0.054	0.053
			0.048	0.075	0.052	0.071	0.042	0.061	0.045	0.058
			0.709	0.916	0.754	0.896	0.849	0.953	0.890	0.943
			0.008	0.007	0.008	0.007	0.003	0.003	0.003	0.003

C.2 Detailed results of simulations in Section A.4.1

Table D. In each cell, from top to bottom are empirical power, mean($\hat{\theta}$), SD($\hat{\theta}$), mean(SE($\hat{\theta}$)), coverage rate, MSE, when $\theta = 0.2$, $m = 100$ and 30% invalid IVs with uncorrelated pleiotropy.

(a) $\rho = 0$

N	cML-BIC-C	cML-BIC-C-MPLE	cML-BIC-I
25,000	1.000e+00<br>2.029e-01<br>1.136e-02<br>9.202e-03<br>8.799e-01<br>1.374e-04	1.000e+00<br>2.029e-01<br>1.136e-02<br>9.137e-03<br>8.771e-01<br>1.374e-04	1.000e+00<br>2.029e-01<br>1.136e-02<br>9.202e-03<br>8.799e-01<br>1.374e-04
100,000	1.000e+00<br>2.004e-01<br>5.337e-03<br>4.696e-03<br>9.130e-01<br>2.862e-05	1.000e+00<br>2.004e-01<br>5.337e-03<br>4.688e-03<br>9.122e-01<br>2.862e-05	1.000e+00<br>2.004e-01<br>5.337e-03<br>4.696e-03<br>9.130e-01<br>2.862e-05
500,000	1.000e+00<br>2.000e-01<br>2.283e-03<br>2.128e-03<br>9.327e-01<br>5.211e-06	1.000e+00<br>2.000e-01<br>2.283e-03<br>2.127e-03<br>9.327e-01<br>5.211e-06	1.000e+00<br>2.000e-01<br>2.283e-03<br>2.128e-03<br>9.327e-01<br>5.211e-06
1,000,000	1.000e+00<br>2.000e-01<br>1.600e-03<br>1.510e-03<br>9.327e-01<br>2.560e-06	1.000e+00<br>2.000e-01<br>1.600e-03<br>1.510e-03<br>9.327e-01<br>2.560e-06	1.000e+00<br>2.000e-01<br>1.600e-03<br>1.510e-03<br>9.327e-01<br>2.560e-06
5,000,000	1.000e+00<br>2.000e-01<br>7.043e-04<br>6.784e-04<br>9.400e-01<br>4.962e-07	1.000e+00<br>2.000e-01<br>7.043e-04<br>6.784e-04<br>9.400e-01<br>4.962e-07	1.000e+00<br>2.000e-01<br>7.043e-04<br>6.784e-04<br>9.401e-01<br>4.962e-07

(b) $\rho = 0.8$

N	cML-BIC-C	cML-BIC-C-MPLE	cML-BIC-I
25,000	1.000e+00<br>2.000e-01<br>9.390e-03<br>7.718e-03<br>8.949e-01<br>8.817e-05	1.000e+00<br>2.000e-01<br>9.390e-03<br>7.690e-03<br>8.938e-01<br>8.817e-05	1.000e+00<br>2.074e-01<br>9.732e-03<br>9.201e-03<br>8.550e-01<br>1.497e-04
100,000	1.000e+00<br>2.000e-01<br>4.416e-03<br>3.925e-03<br>9.204e-01<br>1.950e-05	1.000e+00<br>2.000e-01<br>4.416e-03<br>3.922e-03<br>9.202e-01<br>1.950e-05	1.000e+00<br>2.015e-01<br>4.559e-03<br>4.696e-03<br>9.449e-01<br>2.300e-05
500,000	1.000e+00<br>2.000e-01<br>1.887e-03<br>1.774e-03<br>9.346e-01<br>3.560e-06	1.000e+00<br>2.000e-01<br>1.890e-03<br>1.774e-03<br>9.344e-01<br>3.571e-06	1.000e+00<br>2.002e-01<br>1.931e-03<br>2.128e-03<br>9.670e-01<br>3.783e-06
1,000,000	1.000e+00<br>2.000e-01<br>1.325e-03<br>1.258e-03<br>9.372e-01<br>1.756e-06	1.000e+00<br>2.000e-01<br>1.325e-03<br>1.258e-03<br>9.371e-01<br>1.756e-06	1.000e+00<br>2.001e-01<br>1.349e-03<br>1.510e-03<br>9.700e-01<br>1.831e-06
5,000,000	1.000e+00<br>2.000e-01<br>5.828e-04<br>5.649e-04<br>9.400e-01<br>3.398e-07	1.000e+00<br>2.000e-01<br>5.828e-04<br>5.649e-04<br>9.400e-01<br>3.398e-07	1.000e+00<br>2.000e-01<br>5.906e-04<br>6.784e-04<br>9.743e-01<br>3.489e-07

Table E. In each cell, from top to bottom are empirical power, mean($\hat{\theta}$), SD($\hat{\theta}$), mean(SE($\hat{\theta}$)), coverage rate, MSE, when $\theta = 0.2$, $m = 100$ and 30% invalid IVs with correlated pleiotropy.

(a) $\rho = 0$

N	cML-BIC-C	cML-BIC-C-MPLE	cML-BIC-I
25,000	1.000e+00<br>2.033e-01<br>1.141e-02<br>9.183e-03<br>8.722e-01<br>1.411e-04	1.000e+00<br>2.033e-01<br>1.141e-02<br>9.120e-03<br>8.698e-01<br>1.411e-04	1.000e+00<br>2.033e-01<br>1.141e-02<br>9.183e-03<br>8.722e-01<br>1.411e-04
100,000	1.000e+00<br>2.005e-01<br>5.355e-03<br>4.691e-03<br>9.129e-01<br>2.889e-05	1.000e+00<br>2.005e-01<br>5.355e-03<br>4.684e-03<br>9.125e-01<br>2.889e-05	1.000e+00<br>2.005e-01<br>5.355e-03<br>4.691e-03<br>9.129e-01<br>2.889e-05
500,000	1.000e+00<br>2.000e-01<br>2.294e-03<br>2.127e-03<br>9.299e-01<br>5.261e-06	1.000e+00<br>2.000e-01<br>2.294e-03<br>2.126e-03<br>9.297e-01<br>5.261e-06	1.000e+00<br>2.000e-01<br>2.294e-03<br>2.127e-03<br>9.299e-01<br>5.261e-06
1,000,000	1.000e+00<br>2.000e-01<br>1.607e-03<br>1.509e-03<br>9.358e-01<br>2.581e-06	1.000e+00<br>2.000e-01<br>1.607e-03<br>1.509e-03<br>9.358e-01<br>2.581e-06	1.000e+00<br>2.000e-01<br>1.607e-03<br>1.509e-03<br>9.358e-01<br>2.581e-06
5,000,000	1.000e+00<br>2.000e-01<br>7.029e-04<br>6.784e-04<br>9.416e-01<br>4.941e-07	1.000e+00<br>2.000e-01<br>7.029e-04<br>6.783e-04<br>9.416e-01<br>4.941e-07	1.000e+00<br>2.000e-01<br>7.029e-04<br>6.784e-04<br>9.416e-01<br>4.941e-07

(b) $\rho = 0.8$

N	cML-BIC-C	cML-BIC-C-MPLE	cML-BIC-I
25,000	1.000e+00<br>2.003e-01<br>9.412e-03<br>7.702e-03<br>8.906e-01<br>8.869e-05	1.000e+00<br>2.003e-01<br>9.412e-03<br>7.675e-03<br>8.896e-01<br>8.869e-05	1.000e+00<br>2.077e-01<br>9.793e-03<br>9.182e-03<br>8.453e-01<br>1.554e-04
100,000	1.000e+00<br>2.000e-01<br>4.432e-03<br>3.921e-03<br>9.183e-01<br>1.964e-05	1.000e+00<br>2.000e-01<br>4.432e-03<br>3.918e-03<br>9.178e-01<br>1.964e-05	1.000e+00<br>2.016e-01<br>4.582e-03<br>4.690e-03<br>9.423e-01<br>2.346e-05
500,000	1.000e+00<br>2.000e-01<br>1.900e-03<br>1.774e-03<br>9.323e-01<br>3.610e-06	1.000e+00<br>2.000e-01<br>1.900e-03<br>1.773e-03<br>9.322e-01<br>3.610e-06	1.000e+00<br>2.002e-01<br>1.945e-03<br>2.127e-03<br>9.672e-01<br>3.845e-06
1,000,000	1.000e+00<br>2.000e-01<br>1.319e-03<br>1.258e-03<br>9.372e-01<br>1.740e-06	1.000e+00<br>2.000e-01<br>1.319e-03<br>1.258e-03<br>9.372e-01<br>1.740e-06	1.000e+00<br>2.001e-01<br>1.356e-03<br>1.509e-03<br>9.700e-01<br>1.853e-06
5,000,000	1.000e+00<br>2.000e-01<br>5.805e-04<br>5.649e-04<br>9.431e-01<br>3.370e-07	1.000e+00<br>2.000e-01<br>5.805e-04<br>5.649e-04<br>9.431e-01<br>3.370e-07	1.000e+00<br>2.000e-01<br>5.876e-04<br>6.784e-04<br>9.754e-01<br>3.455e-07

Table F. In each cell, from top to bottom are empirical accuracy, true positive rate, true negative rate, when $\theta = 0.2$, $m = 100$ and 30% invalid IVs with uncorrelated pleiotropy.

(a) $\rho = 0$

n	cML-BIC-I	cML-BIC-C
25,000	9.281e-01<br>7.638e-01<br>9.985e-01	9.281e-01<br>7.638e-01<br>9.985e-01
100,000	9.617e-01<br>8.738e-01<br>9.993e-01	9.617e-01<br>8.738e-01<br>9.993e-01
500,000	9.817e-01<br>9.396e-01<br>9.997e-01	9.817e-01<br>9.396e-01<br>9.997e-01
1,000,000	9.867e-01<br>9.561e-01<br>9.998e-01	9.867e-01<br>9.561e-01<br>9.998e-01
5,000,000	9.938e-01<br>9.794e-01<br>9.999e-01	9.938e-01<br>9.794e-01<br>9.999e-01

(b) $\rho = 0.8$

n	cML-BIC-I	cML-BIC-C
25,000	9.285e-01<br>7.620e-01<br>9.999e-01	9.381e-01<br>8.003e-01<br>9.972e-01
100,000	9.621e-01<br>8.739e-01<br>1.000e+00	9.673e-01<br>8.939e-01<br>9.987e-01
500,000	9.819e-01<br>9.396e-01<br>1.000e+00	9.845e-01<br>9.493e-01<br>9.996e-01
1,000,000	9.868e-01<br>9.560e-01<br>1.000e+00	9.888e-01<br>9.632e-01<br>9.998e-01
5,000,000	9.938e-01<br>9.793e-01<br>1.000e+00	9.948e-01<br>9.828e-01<br>9.999e-01

Table G. In each cell, from top to bottom are empirical accuracy, true positive rate, true negative rate, when $\theta = 0.2$, $m = 100$ and 30% invalid IVs with correlated pleiotropy.

(a) $\rho = 0$

n	cML-BIC-I	cML-BIC-C
25,000	9.331e-01<br>7.806e-01<br>9.985e-01	9.331e-01<br>7.806e-01<br>9.985e-01
100,000	9.644e-01<br>8.831e-01<br>9.993e-01	9.644e-01<br>8.831e-01<br>9.993e-01
500,000	9.831e-01<br>9.443e-01<br>9.997e-01	9.831e-01<br>9.443e-01<br>9.997e-01
1,000,000	9.878e-01<br>9.597e-01<br>9.998e-01	9.878e-01<br>9.597e-01<br>9.998e-01
5,000,000	9.943e-01<br>9.812e-01<br>9.999e-01	9.943e-01<br>9.812e-01<br>9.999e-01

(b) $\rho = 0.8$

n	cML-BIC-I	cML-BIC-C
25,000	9.337e-01<br>7.792e-01<br>9.999e-01	9.427e-01<br>8.153e-01<br>9.974e-01
100,000	9.648e-01<br>8.828e-01<br>1.000e+00	9.696e-01<br>9.016e-01<br>9.987e-01
500,000	9.833e-01<br>9.443e-01<br>1.000e+00	9.858e-01<br>9.536e-01<br>9.996e-01
1,000,000	9.879e-01<br>9.598e-01<br>1.000e+00	9.898e-01<br>9.664e-01<br>9.998e-01
5,000,000	9.944e-01<br>9.812e-01<br>1.000e+00	9.952e-01<br>9.843e-01<br>9.999e-01

C.3 Detailed results of simulations in Section A.4.2

Table H. In each cell, from top to bottom are empirical power, mean($\hat{\theta}$), SD($\hat{\theta}$), mean(SE($\hat{\theta}$)), coverage rate, MSE, when $\theta = 0.2$, $m = 100$, ρ=0.

N	cML-BIC-C-K0	cML-BIC-C-K0-MPLE	cML-BIC-I-K0	cML-BIC-I-K0-robust
25,000	1.000e+00<br>1.999e-01<br>8.092e-03<br>8.062e-03<br>9.504e-01<br>6.548e-05	1.000e+00<br>1.999e-01<br>8.092e-03<br>8.015e-03<br>9.487e-01<br>6.548e-05	1.000e+00<br>1.999e-01<br>8.092e-03<br>8.062e-03<br>9.504e-01<br>6.548e-05	1.000e+00<br>1.999e-01<br>8.092e-03<br>8.028e-03<br>9.456e-01<br>6.548e-05
100,000	1.000e+00<br>1.999e-01<br>4.039e-03<br>4.031e-03<br>9.509e-01<br>1.631e-05	1.000e+00<br>1.999e-01<br>4.039e-03<br>4.025e-03<br>9.505e-01<br>1.631e-05	1.000e+00<br>1.999e-01<br>4.039e-03<br>4.031e-03<br>9.509e-01<br>1.631e-05	1.000e+00<br>1.999e-01<br>4.039e-03<br>4.006e-03<br>9.458e-01<br>1.631e-05
500,000	1.000e+00<br>2.000e-01<br>1.806e-03<br>1.803e-03<br>9.505e-01<br>3.260e-06	1.000e+00<br>2.000e-01<br>1.806e-03<br>1.802e-03<br>9.504e-01<br>3.260e-06	1.000e+00<br>2.000e-01<br>1.806e-03<br>1.803e-03<br>9.505e-01<br>3.260e-06	1.000e+00<br>2.000e-01<br>1.806e-03<br>1.791e-03<br>9.462e-01<br>3.260e-06
1,000,000	1.000e+00<br>2.000e-01<br>1.277e-03<br>1.275e-03<br>9.500e-01<br>1.630e-06	1.000e+00<br>2.000e-01<br>1.277e-03<br>1.275e-03<br>9.499e-01<br>1.630e-06	1.000e+00<br>2.000e-01<br>1.277e-03<br>1.275e-03<br>9.500e-01<br>1.630e-06	1.000e+00<br>2.000e-01<br>1.277e-03<br>1.266e-03<br>9.460e-01<br>1.630e-06
5,000,000	1.000e+00<br>2.000e-01<br>5.710e-04<br>5.701e-04<br>9.498e-01<br>3.261e-07	1.000e+00<br>2.000e-01<br>5.710e-04<br>5.701e-04<br>9.498e-01<br>3.261e-07	1.000e+00<br>2.000e-01<br>5.710e-04<br>5.701e-04<br>9.498e-01<br>3.261e-07	1.000e+00<br>2.000e-01<br>5.710e-04<br>5.662e-04<br>9.462e-01<br>3.261e-07

Table I. In each cell, from top to bottom are empirical power, mean($\hat{\theta}$), SD($\hat{\theta}$), mean(SE($\hat{\theta}$)), coverage rate, MSE, when $\theta = 0.2$, $m = 100$, ρ=0.8.

N	cML-BIC-C-K0	cML-BIC-C-K0-MPLE	cML-BIC-I-K0	cML-BIC-I-K0-robust
25,000	1.000e+00<br>1.999e-01<br>6.747e-03<br>6.710e-03<br>9.487e-01<br>4.554e-05	1.000e+00<br>1.999e-01<br>6.747e-03<br>6.689e-03<br>9.479e-01<br>4.554e-05	1.000e+00<br>2.044e-01<br>6.706e-03<br>8.063e-03<br>9.528e-01<br>6.455e-05	1.000e+00<br>2.044e-01<br>6.706e-03<br>6.626e-03<br>8.897e-01<br>6.455e-05
100,000	1.000e+00<br>1.999e-01<br>3.369e-03<br>3.354e-03<br>9.488e-01<br>1.136e-05	1.000e+00<br>1.999e-01<br>3.369e-03<br>3.352e-03<br>9.488e-01<br>1.136e-05	1.000e+00<br>2.011e-01<br>3.365e-03<br>4.031e-03<br>9.733e-01<br>1.249e-05	1.000e+00<br>2.011e-01<br>3.365e-03<br>3.326e-03<br>9.323e-01<br>1.249e-05
500,000	1.000e+00<br>2.000e-01<br>1.506e-03<br>1.500e-03<br>9.487e-01<br>2.269e-06	1.000e+00<br>2.000e-01<br>1.506e-03<br>1.500e-03<br>9.487e-01<br>2.269e-06	1.000e+00<br>2.002e-01<br>1.506e-03<br>1.803e-03<br>9.789e-01<br>2.310e-06	1.000e+00<br>2.002e-01<br>1.506e-03<br>1.489e-03<br>9.432e-01<br>2.310e-06
1,000,000	1.000e+00<br>2.000e-01<br>1.065e-03<br>1.061e-03<br>9.484e-01<br>1.135e-06	1.000e+00<br>2.000e-01<br>1.065e-03<br>1.061e-03<br>9.484e-01<br>1.135e-06	1.000e+00<br>2.001e-01<br>1.065e-03<br>1.275e-03<br>9.793e-01<br>1.144e-06	1.000e+00<br>2.001e-01<br>1.065e-03<br>1.053e-03<br>9.446e-01<br>1.144e-06
5,000,000	1.000e+00<br>2.000e-01<br>4.763e-04<br>4.743e-04<br>9.482e-01<br>2.269e-07	1.000e+00<br>2.000e-01<br>4.763e-04<br>4.743e-04<br>9.482e-01<br>2.269e-07	1.000e+00<br>2.000e-01<br>4.763e-04<br>5.701e-04<br>9.796e-01<br>2.271e-07	1.000e+00<br>2.000e-01<br>4.763e-04<br>4.711e-04<br>9.467e-01<br>2.271e-07

D Graph-MRcML

D.1 Bidirectional MR-cML-C

To infer a possibly bi-directional causal relationship between a pair of traits, say X and Y, we apply bidirectional MR-cML-C [5]. In practice, we first select (genome-wide) significant (and nearly independent) SNPs for trait X, then do so for Y. Next for each SNP selected for both X and Y, we only use it as IV for the trait that has a larger absolute value of correlation with the SNP; this screening rule combines a simple application of Steiger's method [6] with

the fact that an SNP cannot be a valid IV for both traits at the same time. Then we use the significant SNPs for X after excluding those (if any) based on the above screening rule as the IVs and apply MR-cML-C for causal direction $X \to Y$; similarly, we use the significant SNPs for Y after excluding those (if any) based on the screening rule as the IVs in MR-cML-C for $Y \to X$. In this way, we do not need to specify the causal direction(s) a priori and thus infer a possibly bi-directional relationship between the two traits.

D.2 Effective number of tests

For T traits, $T \times (T-1)$ tests are performed in total. To account for multiple testing, one can use a conservative Bonferroni adjustment with the total number of tests. However, we expect that these $T \times (T-1)$ tests are not independent with each other as we reuse each GWAS dataset multiple times and some of them might have overlapping samples. We take the approach described in [7] to calculate the effective number of independent p-values (M_e). Specifically, in each data perturbation, we could obtain p-values from MR-cML-BIC-C for the $T \times (T-1)$ tests, and thus through hundreds of times of data perturbation, we could obtain a $T(T-1) \times T(T-1)$ sample correlation matrix for the $T \times (T-1)$ tests. Then the effective number of independent tests is estimated as

$$M_e = T(T-1) - \sum_{i=1}^{T(T-1)} I(\omega_i > 1) \times (\omega_i - 1),$$

where ω_i's are the eigenvalues of the $T(T-1) \times T(T-1)$ sample correlation matrix of the p-values and $I(\cdot)$ is the indicator function. We apply the Bonferroni adjustment with M_e and claim an edge in the graph is statistically significant if its p-value was smaller than $0.05/M_e$.

D.3 A simple example of network deconvolution

Here, we illustrate the idea of network deconvolution (Eq (7) in the main text) via a simple mediation analysis with three traits as follows:

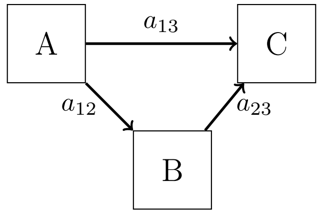

Then the corresponding $\mathbf{G}_{dir}$, $\mathbf{G}_{dir}^2$ and $\mathbf{G}_{dir}^3$ are given as: $\mathbf{G}_{dir} = \begin{bmatrix} 0 & a_{12} & a_{13} \\ 0 & 0 & a_{23} \\ 0 & 0 & 0 \end{bmatrix}$, $\mathbf{G}_{dir}^2 = \begin{bmatrix} 0 & 0 & a_{12}a_{23} \\ 0 & 0 & 0 \\ 0 & 0 & 0 \end{bmatrix}$, $\mathbf{G}_{dir}^3 = \begin{bmatrix} 0 & 0 & 0 \\ 0 & 0 & 0 \\ 0 & 0 & 0 \end{bmatrix}$. And the total network is $\mathbf{G}_{tot} = \begin{bmatrix} 0 & a_{12} & a_{13} + a_{12}a_{23} \\ 0 & 0 & a_{23} \\ 0 & 0 & 0 \end{bmatrix}$. We can see that, the (1,3)th element in $\mathbf{G}_{dir}^2$ corresponds to the indirect effect $a_{12}a_{23}$ from A to C (mediated through B). Since there is no causal pathway of length 3, $\mathbf{G}_{dir}^3$ will be a zero matrix. And the total effect from A to C is $a_{13} + a_{12}a_{23}$. Finally, it is easy to verify that $\mathbf{G}_{tot} = \mathbf{G}_{dir} + \mathbf{G}_{dir}^2 + \mathbf{G}_{dir}^3 + \cdots = \mathbf{G}_{dir} + \mathbf{G}_{dir}^2 = \mathbf{G}_{dir}(\mathbf{I} - \mathbf{G}_{dir})^{-1}$.

D.4 The diagonal elements of a total graph

In the main text, we proposed **Graph-MRcML-d0** and **Graph-MRcML-d1** with two ways to specify the diagonal elements of a total graph. In Graph-MRcML-d0, we set the diagonal elements to zeros, following the practice in [8]. However, this may be problematic if there are cycles in the underlying direct graph, thus we proposed the following iterative algorithm to update the diagonal elements of $\mathbf{G}_{tot}$ in Graph-MRcML-d1. Denote $\mathbf{G}_{tot} = (T_{ij})$, $\mathbf{G}_{dir} = (D_{ij})$ and the bidirectional MR-cML estimates $\hat{T}_{ij}$ $(i \neq j)$.

Algorithm 1 Estimation of $\mathbf{G}_{dir}$ in Graph-MRcML-d1

$\hat{T}_{ii} \leftarrow \sum_{j \neq i} \hat{T}_{ji}\hat{T}_{ij}$
$\widehat{\mathbf{G}}^0_{dir}, \widehat{\mathbf{G}}_{dir} \leftarrow \widehat{\mathbf{G}}_{tot}(\mathbf{I} + \widehat{\mathbf{G}}_{tot})^{-1}$
$t \leftarrow 0$
while $|\hat{D}_{ii}| > \epsilon$ and $t <$ maxit **do** ▷ ϵ is a small value, e.g. 10^{-4}
    $\hat{T}_{ii} \leftarrow \sum_{j \neq i} \hat{D}_{ji}\hat{T}_{ij}$ ▷ Eq 9 in the main text
    $\widehat{\mathbf{G}}_{dir} \leftarrow \widehat{\mathbf{G}}_{tot}(\mathbf{I} + \widehat{\mathbf{G}}_{tot})^{-1}$
    $t \leftarrow t + 1$
end while
if $t =$ maxit **then**
    $\widehat{\mathbf{G}}_{dir} \leftarrow \widehat{\mathbf{G}}^0_{dir}$
end if
return $\widehat{\mathbf{G}}_{dir}$

The proposed iterative algorithm is motivated and illustrated by the following examples.

D.4.1 Example 1

Consider the true direct graph ($\mathbf{G}_{dir}$) with edges $A \to B, B \to C$ and $C \to A$, and all with effect size of 0.6 (Table Ja). The corresponding true total graph ($\mathbf{G}_{tot}$) is shown in Table Jb (round to 7 decimal places), which has non-zero diagonal elements (0.2755102, 0.2755102, 0.2755102). Incorrectly specifying the diagonal elements of $\mathbf{G}_{tot}$ to zeros (as in Graph-MRcML-d0) would lead to an incorrect direct graph as shown in Table Jc.

Table J. Example 1: (a) true direct graph, (b) true total graph and (c) incorrect direct graph by setting diag($\mathbf{G}_{tot}$) to zeros, among three nodes.

	A	B	C
A	0	0.6	0
B	0	0	0.6
C	0.6	0	0

(a) True direct graph

	A	B	C
A	0.2755102	0.7653061	0.4591837
B	0.4591837	0.2755102	0.7653061
C	0.7653061	0.4591837	0.2755102

(b) True total graph

	A	B	C
A	-0.3214689	1.1296873	-0.2577596
B	-0.2577596	-0.3214689	1.1296873
C	1.1296873	-0.2577596	-0.3214689

(c) Incorrect direct graph

We applied Algorithm 1 to iteratively estimate $\mathbf{G}_{dir}$, and plotted the values of $\hat{D}_{11}$, $\hat{D}_{12}$ and $\hat{D}_{13}$ in each iteration as shown in the black line in Fig P. We can see that they converged to the true values (in red) in 8 iterations (so did other entries not plotted here), while the blue dashed line was the estimate in Table Jc.

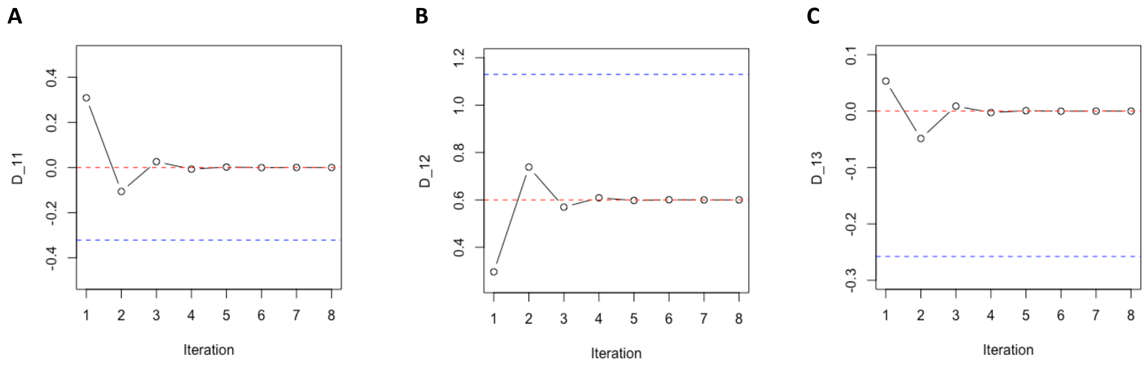

Fig P. Example 1: values of D_{11} (panel A), D_{12} (panel B) and D_{13} (panel C) estimates in each iteration (black line). Dashed line in red is the true value and dashed line in blue is the estimate by setting diagonal elements of the total graph to zeros.

D.4.2 Example 2

Here we give an example that Algorithm 1 failed to converge, but using the result in the first iteration (i.e., $\widehat{\mathbf{G}}^0_{dir}$) was able to improve the result over that by simply setting the diagonal elements to zeros. The true direct graph, true total graph and the incorrect direct graph are given in Table Ka, Table Kb, Table Kc respectively. As shown in the panel A in Fig Q, Algorithm 1 failed to converge (as $\hat{D}_{11}$ failed to converge to 0), but the initial estimates (as shown in iteration 1) were much closer to the truth (in red) than to the blue lines. The initial estimate $\widehat{\mathbf{G}}^0_{dir}$ is given in Table Kd, which was closer to the truth (Table Ka) than that given in Table Kc. In fact, the spectral radius of Table Kc was also larger than 1, casting doubt on its reliability.

Table K. Example 2: (a) true direct graph, (b) true total graph, (c) incorrect direct graph by setting diag($\mathbf{G}_{tot}$) to zeros, and (d) initial estimate $\widehat{\mathbf{G}}^0_{dir}$ among three nodes.

	A	B	C
A	0	0.5	0.4
B	0.4	0	0.5
C	0.5	0	0

(a) True direct graph

	A	B	C
A	1.105263	1.052632	1.368421
B	1.368421	0.684211	1.389474
C	1.052632	0.526316	0.684211

(b) True total graph

	A	B	C
A	4.087102	-3.819095	1.082077
B	1.082077	-4.060302	5.550419
C	-3.819095	6.683417	-4.060302

(c) Incorrect direct graph

	A	B	C
A	0.684749	0.088488	0.097248
B	0.097248	0.632704	0.118948
C	0.088488	0.031582	0.632704

(d) $\widehat{\mathbf{G}}^0_{dir}$

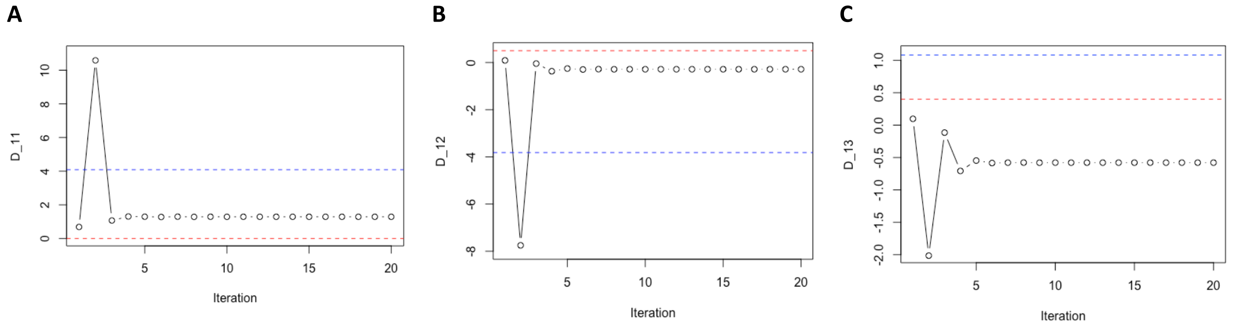

Fig Q. Example 2: values of D_{11} (panel A), D_{12} (panel B) and D_{13} (panel C) estimates in each iteration (black line). Dashed line in red is the true value and dashed line in blue is the estimate by setting diagonal elements of the total graph to zeros.

D.5 Simulation for direct graph inference in the main text

We simulated data based on the estimated direct graph by Graph-MRcML-d0 among 6 traits as shown in Table L. We point out that in Set-up (a), there was no loop, and thus the diagonal elements of the total graph were all zeros. But in Set-up (b), there was a cycle between CAD and AF, and their corresponding diagonal elements in the total graph were not zeros (Table Ma). Table Mb shows the resulting (incorrect) direct graph if we set the diagonal elements of the total graph to zeros in Set-up (b).

Table L. True direct graphs in the simulation for (a) Set-up (a), and (b) Set-up (b).

	BMI	LDL	FG	TG	CAD	Stroke
BMI	0	0	0.07	0.23	0.23	0
LDL	0	0	0	0	0.41	0
FG	0	0	0	0	0.31	0
TG	0	0	0	0	0	0
CAD	0	0	0	0	0	0.23
Stroke	0	0	0	0	0	0

(a) Set-up (a)

	BMI	LDL	FG	TG	CAD	AF
BMI	0	0	0.08	0.23	0.22	0.32
LDL	0	0	0	0	0.42	0
FG	0	0	0	0	0.33	0
TG	0	0	0	0	0	0
CAD	0	0	0	0	0	0.17
AF	0	0	0	0	0.10	0

(b) Set-up (b)

Table M. In Set-up (b): (a) the true total graph $\mathbf{G}_{tot}$, and (b) resulting direct graph when specifying diag($\mathbf{G}_{tot}$) to zeros. Numbers are rounded to 3 decimal places.

	BMI	LDL	FG	TG	CAD	AF
BMI	0	0	0.08	0.23	0.283	0.368
LDL	0	0	0	0	0.427	0.073
FG	0	0	0	0	0.336	0.057
TG	0	0	0	0	0	0
CAD	0	0	0	0	0.017	0.173
AF	0	0	0	0	0.102	0.017

(a)

	BMI	LDL	FG	TG	CAD	AF
BMI	0	0	0.08	0.23	0.223	0.325
LDL	0	0	0	0	0.427	-0.001
FG	0	0	0	0	0.336	-0.001
TG	0	0	0	0	0	0
CAD	0	0	0	0	-0.018	0.176
AF	0	0	0	0	0.104	-0.018

(b)

Let BMI, LDL, FG, TG, CAD and Stroke (or AF) be the first, second, ..., and the 6-th trait respecitvely. Then we generated the *true* association effect sizes $\mathbf{B} = (b_{ij})$, where b_{ij} was the association effect size between the i-th SNP and the j-th trait, as follows. First we initialized $\mathbf{B}$ as a zero matrix. Then for the j-th trait, $j = 1, 2, \ldots, 6$,

1. for $i \in \mathcal{S}_j$, where $\mathcal{S}_j$ is the set of GWAS significant SNPs (i.e. p-value<5e-8) for the j-th trait, let $b_{ij} = (1 + T_{jj}) * \hat{\beta}_{ij}$, where $\hat{\beta}_{ij}$ is the GWAS estimate from the corresponding GWAS dataset used in the real data analysis, and T_{jj} is the j-th diagonal of $\mathbf{G}_{tot}$;

2. for the k-th trait, $k \neq j$, let $b_{ik} = \theta_{jk} b_{ij}$, $i \in \mathcal{S}_j$, where θ_{jk} is the true total effect size from the j-th trait to the k-th trait.

With this data-generating procedure, some invalid IVs (with horizontal pleiotropy) were also generated for some pairs of traits, as some traits (e.g. LDL and TG) shared common IVs in nature. Then with the generated $\mathbf{B}$, in each simulation replicate, we simulated the *estimated*

GWAS summary statistics as $\widehat{\mathbf{B}} = \mathbf{B} + \mathbf{S} * \mathbf{E}$, where $\mathbf{S}$ was the standard errors of GWAS estimates obtained from the real GWAS summary statistics. Alternatively we considered different GWAS sample sizes by setting $\mathbf{S} = (S_{ij})$, and $S_{ij} = 1/\sqrt{N}$, $N = 300\,000$, $500\,000$ or $1\,000\,000$ for all 6 traits. $\mathbf{E}$ was generated from a matrix normal distribution as in data perturbation, $\mathbf{E} \sim \mathcal{MN}(\mathbf{0}, \mathbf{R}, \mathbf{P})$, and $\mathbf{R}$ and $\mathbf{P}$ were the LD matrix of the SNPs and the correlation matrix among the 6 GWAS traits respectively, estimated from the real GWAS data as well.

D.5.1 Results with S from real GWAS summary statistics

Table N. Empirical type-I error and power by Graph-MRcML-d0 for (a) Set-up (a) and (b) Set-up (b). Numbers underlined correspond to power.

	BMI	LDL	FG	TG	CAD	Stroke
BMI		0	0.6	1	0.9	0
LDL	0		0	0.01	1	0
FG	0	0		0	0.89	0
TG	0	0.01	0		0.06	0.01
CAD	0	0	0	0		1
Stroke	0	0.01	0	0	0	

(a) Set-up (a)

	BMI	LDL	FG	TG	CAD	AF
BMI		0	0.81	1	0.87	1
LDL	0.02		0	0	1	0
FG	0	0		0	0.97	0
TG	0	0	0		0.01	0
CAD	0	0	0	0		1
AF	0	0	0	0	1	

(b) Set-up (b)

Table O. Set-up (a): mean estimated direct graph by (a) Graph-MRcML-d0, and (b) Graph-MRcML-d1 across 100 replicates. Numbers are rounded to 3 decimal places.

	BMI	LDL	FG	TG	CAD	Stroke
BMI	0	-0.001	0.069	0.231	0.233	-0.006
LDL	0	0	0.001	0	0.409	0.005
FG	0.001	0.002	0	0.002	0.311	0
TG	0	0	0	0	-0.004	0.019
CAD	0	0	0	0	0	0.23
Stroke	0.001	0	-0.002	0	0	0

(a) Graph-MRcML-d0

	BMI	LDL	FG	TG	CAD	Stroke
BMI	0	-0.001	0.069	0.23	0.233	-0.006
LDL	0	0	0.001	0	0.409	0.005
FG	0.001	0.002	0	0.002	0.311	0
TG	0	0	0	0	-0.004	0.019
CAD	0	0	0	0	0	0.23
Stroke	0.001	0	-0.002	0	0	0

(b) Graph-MRcML-d1

Table P. Set-up (b): mean estimated direct graph by (a) Graph-MRcML-d0, and (b) Graph-MRcML-d1 across 100 replicates. Numbers are rounded to 3 decimal places.

	BMI	LDL	FG	TG	CAD	AF
BMI	0	-0.001	0.083	0.232	0.19	0.309
LDL	0	0	-0.001	-0.004	0.45	0.004
FG	0.001	0.002	0	0.004	0.339	0
TG	-0.001	0.001	0	0	0.08	0.018
CAD	0.001	0	0.001	0	-0.018	0.174
AF	-0.001	0	0	0	0.104	-0.018

(a) Graph-MRcML-d0

	BMI	LDL	FG	TG	CAD	AF
BMI	0	-0.001	0.083	0.232	0.188	0.305
LDL	0	0	-0.001	-0.004	0.442	0.006
FG	0	0.002	0	0.004	0.332	0.001
TG	-0.001	0.001	0	0	0.079	0.018
CAD	0.001	0	0.001	0	0	0.168
AF	-0.001	0	0	0	0.1	0

(b) Graph-MRcML-d1

D.5.2 Results with varying sample sizes

Set-up (a) In the absence of cycles in the direct graph, the diagonal elements of the true total graph were zeros, and Graph-MRcML-d0 correctly specified them. When the diagonal elements were consistently specified, the resulting estimate of the direct graph was consistent. Tables Q to S show the mean of the estimated direct graphs across 100 simulation replicates by Graph-MRcML-d0 (Panel (a)) and Graph-MRcML-d1 (Panel (b)) for different sample sizes; Tables T to V show the empirical type-I error and power. As expected, the estimate from Graph-MRcML-d0 had smaller bias as sample size increased. Also, Graph-MRcML-d1 performed almost identically as Graph-MRcML-d0, suggesting that the iterative algorithm was able to correctly specify the diagonal elements (of the total graph) when there was no cycle in the direct graph. Type-I error was also controlled as the sample size increased.

Table Q. Set-up (a): mean estimated direct graph by (a) Graph-MRcML-d0, and (b) Graph-MRcML-d1 across 100 replicates when $N = 300\,000$. Numbers are rounded to 3 decimal places.

	BMI	LDL	FG	TG	CAD	Stroke
BMI	0	-0.001	0.069	0.231	0.233	-0.006
LDL	0	0	0.001	0	0.409	0.005
FG	0.001	0.002	0	0.002	0.311	0
TG	0	0	0	0	-0.004	0.019
CAD	0	0	0	0	0	0.23
Stroke	0.001	0	-0.002	0	0	0

(a) Graph-MRcML-d0

	BMI	LDL	FG	TG	CAD	Stroke
BMI	0	-0.001	0.069	0.23	0.233	-0.006
LDL	0	0	0.001	0	0.409	0.005
FG	0.001	0.002	0	0.002	0.311	0
TG	0	0	0	0	-0.004	0.019
CAD	0	0	0	0	0	0.23
Stroke	0.001	0	-0.002	0	0	0

(b) Graph-MRcML-d1

Table R. Set-up (a): mean estimated direct graph by (a) Graph-MRcML-d0, and (b) Graph-MRcML-d1 across 100 replicates when $N = 500\,000$. Numbers are rounded to 3 decimal places.

	BMI	LDL	FG	TG	CAD	Stroke
BMI	0	-0.001	0.069	0.23	0.232	-0.004
LDL	0	0	0	0	0.409	0.003
FG	0.001	0.002	0	0.001	0.31	0
TG	0	0	0	0	-0.002	0.013
CAD	0	0	0	0	0	0.23
Stroke	0.001	0	-0.002	0	0	0

(a) Graph-MRcML-d0

	BMI	LDL	FG	TG	CAD	Stroke
BMI	0	-0.001	0.069	0.23	0.232	-0.004
LDL	0	0	0	0	0.41	0.003
FG	0.001	0.002	0	0.001	0.311	0
TG	0	0	0	0	-0.002	0.013
CAD	0	0	0	0	0	0.23
Stroke	0.001	0	-0.002	0	0	0

(b) Graph-MRcML-d1

Table S. Set-up (a): mean estimated direct graph by (a) Graph-MRcML-d0, and (b) Graph-MRcML-d1 across 100 replicates when $N = 1\,000\,000$. Numbers are rounded to 3 decimal places.

	BMI	LDL	FG	TG	CAD	Stroke
BMI	0	-0.001	0.07	0.23	0.231	-0.001
LDL	0	0	0	0	0.41	0.001
FG	0.001	0.001	0	0.001	0.31	0
TG	0	0	0	0	0	0.005
CAD	0	0	0	0	0	0.23
Stroke	0	0	-0.001	0	0	0

(a) Graph-MRcML-d0

	BMI	LDL	FG	TG	CAD	Stroke
BMI	0	-0.001	0.07	0.23	0.231	-0.001
LDL	0	0	0	0	0.41	0.001
FG	0.001	0.001	0	0.001	0.31	0
TG	0	0	0	0	0	0.005
CAD	0	0	0	0	0	0.23
Stroke	0	0	-0.001	0	0	0

(b) Graph-MRcML-d1

Table T. Set-up (a): empirical type-I error and power by (a) Graph-MRcML-d0, and (b) Graph-MRcML-d1 when $N = 300\,000$. Numbers underlined correspond to power.

	BMI	LDL	FG	TG	CAD	Stroke
BMI		0	$\underline{1}$	$\underline{1}$	$\underline{1}$	0.01
LDL	0		$\underline{0}$	0.01	$\underline{1}$	0.05
FG	0	0		0	$\underline{1}$	0
TG	0	0.01	0.01		$\underline{0}$	0.09
CAD	0	0	0	0		$\underline{1}$
Stroke	0	0	0	0	0	

(a) Graph-MRcML-d0

	BMI	LDL	FG	TG	CAD	Stroke
BMI		0	$\underline{1}$	$\underline{1}$	$\underline{1}$	0.01
LDL	0		$\underline{0}$	0.01	$\underline{1}$	0.06
FG	0	0		0	$\underline{1}$	0
TG	0	0.01	0.01		$\underline{0}$	0.09
CAD	0	0	0	0		$\underline{1}$
Stroke	0	0	0	0	0	

(b) Graph-MRcML-d1

Table U. Set-up (a): empirical type-I error and power by (a) Graph-MRcML-d0, and (b) Graph-MRcML-d1 when $N = 500\,000$. Numbers underlined correspond to power.

	BMI	LDL	FG	TG	CAD	Stroke
BMI		0	1	1	1	0
LDL	0		0	0.01	1	0.02
FG	0	0		0	1	0
TG	0	0.01	0.01		0	0.05
CAD	0	0	0	0		1
Stroke	0	0.01	0	0	0	

(a) Graph-MRcML-d0

	BMI	LDL	FG	TG	CAD	Stroke
BMI		0	1	1	1	0
LDL	0		0	0.01	1	0.01
FG	0	0		0	1	0
TG	0	0.01	0.01		0	0.05
CAD	0	0	0	0		1
Stroke	0	0.01	0	0	0	

(b) Graph-MRcML-d1

Table V. Set-up (a): empirical type-I error and power by (a) Graph-MRcML-d0, and (b) Graph-MRcML-d1 when $N = 1\,000\,000$. Numbers underlined correspond to power.

	BMI	LDL	FG	TG	CAD	Stroke
BMI		0	1	1	1	0
LDL	0.01		0	0.01	1	0
FG	0	0		0	1	0
TG	0	0.01	0.01		0	0
CAD	0	0	0	0		1
Stroke	0	0.01	0	0	0	

(a) Graph-MRcML-d0

	BMI	LDL	FG	TG	CAD	Stroke
BMI		0	1	1	1	0
LDL	0.01		0	0.01	1	0
FG	0	0		0	1	0
TG	0	0.01	0.01		0	0
CAD	0	0	0	0		1
Stroke	0	0.01	0	0	0	

(b) Graph-MRcML-d1

Set-up (b) In the presence of a cycle in the direct graph, the diagonal elements of the true total graph were not all zeros, and incorrectly specifying them to zeros led to an incorrect direct graph given in Table Mb. We can see that, as the sample size increased, the estimates from Graph-MRcML-d0 (Tables Wa, Xa and Ya) approached the incorrect direct graph Table Mb. But the type-I error was controlled in the simulation as the sample size increased, probably because the incorrectly inferred direct effects were small (e.g. LDL $\rightarrow$ AF and FG $\rightarrow$ AF both had -0.001 in Table Mb). On the other hand, the iterative algorithm in Graph-MRcML-d1 converged successfully, and its estimates approached the true direct graph as the sample size increased. Type-I error was also well-controlled as the sample size increased.

Table W. Set-up (b): mean estimated direct graph by (a) Graph-MRcML-d0, and (b) Graph-MRcML-d1 across 100 replicates when $N = 300\,000$. Numbers are rounded to 3 decimal places.

	BMI	LDL	FG	TG	CAD	AF
BMI	0	0	0.081	0.23	0.224	0.32
LDL	0	0	0	-0.001	0.427	0.005
FG	0.001	0.001	0	0.002	0.336	-0.001
TG	-0.001	0	0	0	-0.006	0.02
CAD	0	0	0	0	-0.018	0.176
AF	0	0	0	0	0.104	-0.018

(a) Graph-MRcML-d0

	BMI	LDL	FG	TG	CAD	AF
BMI	0	0	0.081	0.23	0.22	0.315
LDL	0	0	0	-0.001	0.42	0.006
FG	0.001	0.001	0	0.002	0.33	0
TG	-0.001	0	0	0	-0.006	0.02
CAD	0	0	0	0	0	0.17
AF	0	0	0	0	0.1	0

(b) Graph-MRcML-d1

Table X. Set-up (b): mean estimated direct graph by (a) Graph-MRcML-d0, and (b) Graph-MRcML-d1 across 100 replicates when $N = 500\,000$. Numbers are rounded to 3 decimal places.

	BMI	LDL	FG	TG	CAD	AF
BMI	0	0	0.081	0.23	0.223	0.321
LDL	0	0	0	0	0.427	0.004
FG	0.001	0.001	0	0.001	0.336	-0.001
TG	-0.001	0	0	0	-0.003	0.015
CAD	0	0	0	0	-0.018	0.176
AF	0	0	0	0	0.104	-0.018

(a) Graph-MRcML-d0

	BMI	LDL	FG	TG	CAD	AF
BMI	0	0	0.081	0.23	0.22	0.316
LDL	0	0	0	0	0.42	0.005
FG	0.001	0.001	0	0.001	0.33	0
TG	-0.001	0	0	0	-0.003	0.015
CAD	0	0	0	0	0	0.17
AF	0	0	0	0	0.1	0

(b) Graph-MRcML-d1

Table Y. Set-up (b): mean estimated direct graph by (a) Graph-MRcML-d0, and (b) Graph-MRcML-d1 across 100 replicates when $N = 1\,000\,000$. Numbers are rounded to 3 decimal places.

	BMI	LDL	FG	TG	CAD	AF
BMI	0	0	0.081	0.23	0.223	0.322
LDL	0	0	0	0	0.427	0.002
FG	0.001	0.001	0	0.001	0.336	-0.001
TG	0	0	0	0	-0.001	0.01
CAD	0	0	0	0	-0.018	0.176
AF	0	0	0	0	0.104	-0.018

(a) Graph-MRcML-d0

	BMI	LDL	FG	TG	CAD	AF
BMI	0	0	0.081	0.23	0.22	0.318
LDL	0	0	0	0	0.42	0.003
FG	0.001	0.001	0	0.001	0.33	0
TG	0	0	0	0	-0.001	0.009
CAD	0	0	0	0	0	0.17
AF	0	0	0	0	0.1	0

(b) Graph-MRcML-d1

Table Z. Set-up (b): empirical type-I error and power by (a) Graph-MRcML-d0, and (b) Graph-MRcML-d1 when $N = 300\,000$. Numbers underlined correspond to power.

	BMI	LDL	FG	TG	CAD	AF
BMI		0	1	1	1	1
LDL	0		0	0	1	0.02
FG	0	0		0	1	0
TG	0	0	0		0	0.17
CAD	0	0	0	0		1
AF	0	0	0	0	1	

(a) Graph-MRcML-d0

	BMI	LDL	FG	TG	CAD	AF
BMI		0	1	1	1	1
LDL	0		0	0	1	0.12
FG	0	0		0	1	0
TG	0	0	0		0	0.17
CAD	0	0	0	0		1
AF	0	0	0	0	1	

(b) Graph-MRcML-d1

Table AA. Set-up (b): empirical type-I error and power by (a) Graph-MRcML-d0, and (b) Graph-MRcML-d1 when $N = 500\,000$. Numbers underlined correspond to power.

	BMI	LDL	FG	TG	CAD	AF
BMI		0	1	1	1	1
LDL	0.01		0	0	1	0.01
FG	0	0		0.01	1	0
TG	0	0	0		0	0.09
CAD	0	0	0	0		1
AF	0	0	0	0	1	

(a) Graph-MRcML-d0

	BMI	LDL	FG	TG	CAD	AF
BMI		0	1	1	1	1
LDL	0.01		0	0	1	0.11
FG	0	0		0.01	1	0
TG	0	0	0		0	0.09
CAD	0	0	0	0		1
AF	0	0	0	0	1	

(b) Graph-MRcML-d1

Table AB. Set-up (b): empirical type-I error and power by (a) Graph-MRcML-d0, and (b) Graph-MRcML-d1 when $N = 1\,000\,000$. Numbers underlined correspond to power.

	BMI	LDL	FG	TG	CAD	AF
BMI		0	1	1	1	1
LDL	0.01		0	0.01	1	0.01
FG	0	0		0	1	0
TG	0	0	0		0	0.06
CAD	0	0	0	0		1
AF	0	0.01	0	0	1	

(a) Graph-MRcML-d0

	BMI	LDL	FG	TG	CAD	AF
BMI		0	1	1	1	1
LDL	0.01		0	0.01	1	0.03
FG	0	0		0	1	0
TG	0	0	0		0	0.06
CAD	0	0	0	0		1
AF	0	0.01	0	0	1	

(b) Graph-MRcML-d1

D.5.3 Results with more IVs for FG

In our real data analysis, the number of IVs used in each MR analysis ranged from 6 to 374, with fasting glucose (FG) having the least IVs. As shown in [1], using more (valid) IVs in the MR-cML analysis will yield a more precise causal effect estimate (in the first step of Graph-MRcML). And this in turn will affect the direct effect estimates as well. In this

section, we performed additional simulations based on Set-up (a) to study the influence of increasing number of IVs for FG in Graph-MRcML, especially in the second step. In Set-up (a), the number of IVs for FG ranged from 7 to 12 in the MR analyses. We added 50 extra IVs for FG (i.e., SNPs only significantly associated with FG), and performed the simulation 100 times with $N = 1\,000\,000$. We compared the standard deviations of the causal network estimates with and without the extra IVs for FG. For the estimated total network in step one (Table AC), as expected, only the row corresponding to FG (i.e., FG being the exposure) showed more precise estimates when more IVs for FG were used (right panel), while for other total effect estimates their precision remained the same. For the estimated direct network (Table AD), the row corresponding to FG also gave more precise estimates, but not for other direct effect estimates.

Table AC. Set-up (a): standard deviations ($\times 10^{-3}$) of estimated total networks across 100 replicates with 0 extra IV (left) and 50 extra IVs (right) for FG.

	BMI	LDL	FG	TG	CAD	Stroke
BMI		4.0	4.4	4.0	5.1	3.8
LDL	1.1		1.2	1.3	1.6	2.1
FG	7.0	7.0		6.9	7.6	6.5
TG	2.3	3.6	2.3		3.9	5.2
CAD	1.3	2.4	1.6	1.5		1.2
Stroke	5.4	4.5	4.5	4.5	4.4	

	BMI	LDL	FG	TG	CAD	Stroke
BMI		4.0	4.4	4.0	5.1	3.8
LDL	1.1		1.2	1.3	1.6	2.1
FG	2.6	2.4		3.0	2.6	2.3
TG	2.3	3.6	2.3		3.9	5.2
CAD	1.3	2.4	1.6	1.5		1.2
Stroke	5.4	4.5	4.5	4.5	4.4	

Table AD. Set-up (a): standard deviations ($\times 10^{-3}$) of estimated direct networks across 100 replicates with 0 extra IV (left) and 50 extra IVs (right) for FG.

	BMI	LDL	FG	TG	CAD	Stroke
BMI		4.4	4.4	4.2	5.8	4.4
LDL	1.0		1.3	1.5	1.7	2.1
FG	6.9	7.0		7.1	8.6	6.8
TG	2.3	3.6	2.3		4.5	5.2
CAD	1.7	2.5	1.7	1.6		1.3
Stroke	5.4	4.5	4.5	4.4	4.9	

	BMI	LDL	FG	TG	CAD	Stroke
BMI		4.3	4.4	4.1	5.8	4.5
LDL	1.0		1.3	1.5	1.7	2.1
FG	2.6	2.6		3.1	2.9	2.4
TG	2.3	3.6	2.3		4.5	5.2
CAD	1.7	2.5	1.7	1.6		1.3
Stroke	5.4	4.5	4.5	4.4	4.9	

For the purpose of simulation, we also considered the scenario with a larger BMI $\rightarrow$ FG effect of 1, which originally was 0.07 in Set-up (a). We performed the similar analysis and compared the standard deviations of estimated causal networks with and without extra IVs

for FG. Again, the precision of the total effect estimates remained the same between the left and right tables in Table AE except for those using FG as the exposure. But for the estimated direct network (Table AF), the rows corresponding to both FG and BMI showed more precise estimates. This result was not completely surprising. Consider a relevant but simpler example of mediation analysis $A \to B \to C$ (e.g., BMI, FG and CAD being traits A, B, C respectively). Denote x the direct effect estimate of $A \to B$, y the direct effect estimate of $B \to C$, z the total effect estimate of $A \to C$. Then the direct effect estimate of $A \to C$ is $z - xy$. Assuming independence among x, y, z, then $\text{Var}(z - xy) = \text{Var}(z) + \text{Var}(xy) = \text{Var}(z) + \text{Var}(x)E(y)^2 + \text{Var}(y)E(x)^2 + \text{Var}(x)\text{Var}(y)$. Adding extra IVs for FG reduced $\text{Var}(y)$, and when $E(x)$ became larger, the term $\text{Var}(y)E(x)^2$ contributed more in the reduction of $\text{Var}(z - xy)$. When $E(x) = 0.07$, this term became negligible and we didn't observe any increase in the precision of the direct effect estimates of BMI to other traits as shown in Table AD; when $E(x) = 1$, we observed more precise estimates in the row corresponding to BMI in Table AF. In general, when we have more (valid) IVs for a trait, the precision of the direct effect estimates starting from that trait is likely to increase, while the precision for other direct effect estimates may or may not change much, depending on the underlying relationship among the traits.

Table AE. Set-up (a) with a larger BMI $\to$ FG effect: standard deviations ($\times 10^{-3}$) of estimated total networks across 100 replicates with 0 extra IV (left) and 50 extra IVs (right) for FG.

	BMI	LDL	FG	TG	CAD	Stroke
BMI		4.0	5.8	4.0	5.6	3.9
LDL	1.1		1.2	1.3	1.6	2.1
FG	7.0	7.0		6.9	7.6	6.5
TG	2.3	3.6	2.3		3.9	5.2
CAD	1.3	2.4	1.6	1.5		1.2
Stroke	5.4	4.5	4.5	4.5	4.4	

	BMI	LDL	FG	TG	CAD	Stroke
BMI		4.0	5.8	4.0	5.6	3.9
LDL	1.1		1.2	1.3	1.6	2.1
FG	2.6	2.4		3.0	2.6	2.3
TG	2.3	3.6	2.3		3.9	5.2
CAD	1.3	2.4	1.6	1.5		1.2
Stroke	5.4	4.5	4.5	4.5	4.4	

Table AF. Set-up (a) with a larger BMI → FG effect: standard deviations ($\times 10^{-3}$) of estimated direct networks across 100 replicates with 0 extra IV (left) and 50 extra IVs (right) for FG.

	BMI	LDL	FG	TG	CAD	Stroke
BMI		8.3	9.5	8.5	11.1	7.7
LDL	1.0		1.7	1.5	1.7	2.1
FG	6.9	7.0		7.1	9.2	6.8
TG	2.3	3.6	3.3		4.5	5.2
CAD	1.7	2.5	2.4	1.6		1.3
Stroke	5.4	4.5	6.6	4.4	5.0	

	BMI	LDL	FG	TG	CAD	Stroke
BMI		5.0	6.2	5.1	7.3	5.4
LDL	1.0		1.7	1.5	1.7	2.1
FG	2.6	2.6		3.1	3.1	2.4
TG	2.3	3.6	3.3		4.5	5.2
CAD	1.7	2.5	2.4	1.6		1.3
Stroke	5.4	4.5	6.7	4.4	4.9	

E More results from the real data analysis

E.1 GWAS summary data

Table AG. 17 GWAS summary data used in the real data analysis.

GWAS Trait	ID in IEU data base	Reference
LDL	ebi-a-GCST002222	[9]
HDL	ebi-a-GCST002223	[9]
Triglycerides	ebi-a-GCST002216	[9]
Height	ieu-a-89	[10]
BMI	ieu-a-835	[11]
Birth weight	ieu-a-1083	[12]
DBP	ukb-a-359	Neale Lab
SBP	ukb-a-360	Neale Lab
Fasting glucose	ebi-a-GCST000568	[13]
Smoke	ieu-b-25	[14]
Alcohol	ieu-b-73	[14]
CAD	ebi-a-GCST005195	[15]
Stroke	ebi-a-GCST005838	[16]
T2D	ieu-a-26	[17]
Asthma	ebi-a-GCST006862	[18]
AFib	ebi-a-GCST006414	[19]
Alzheimer's disease	Not available	[20]

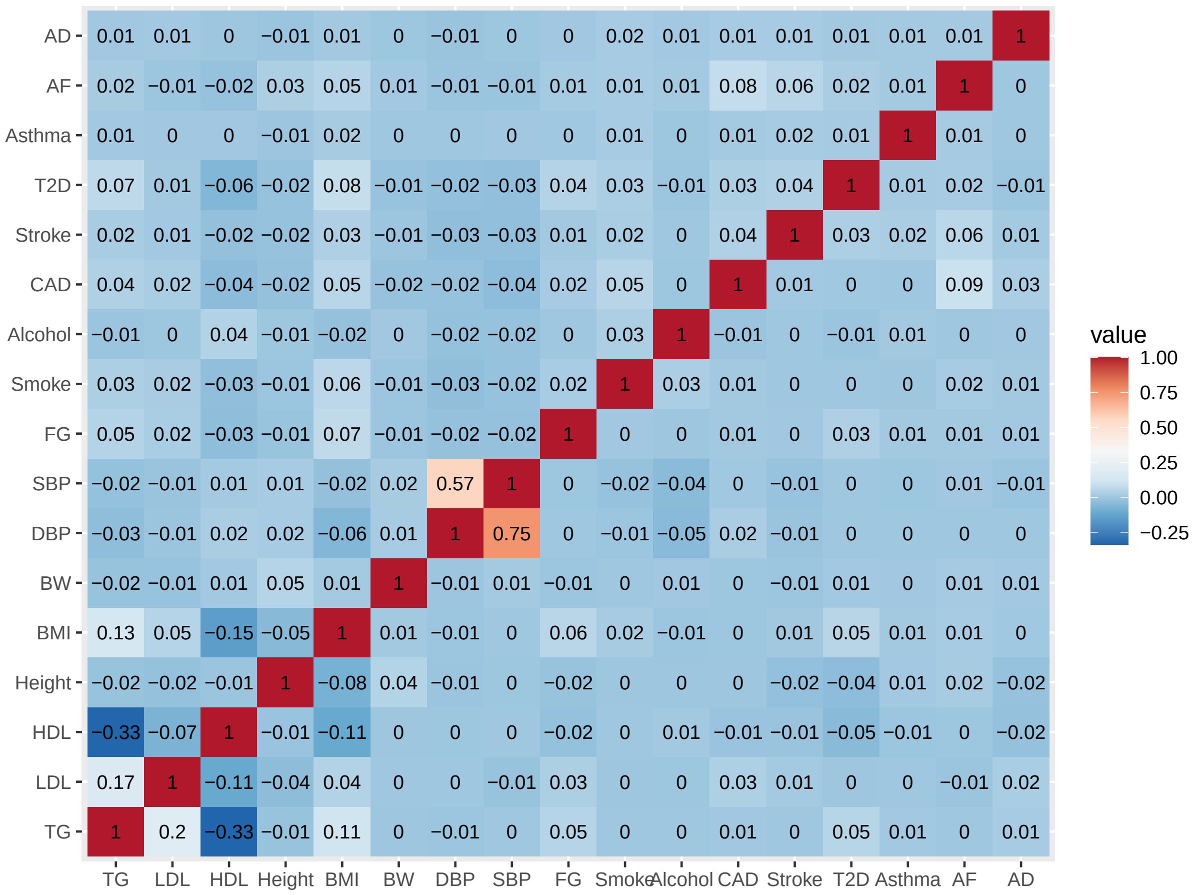

Fig R. The estimated correlation matrix **P** for the 17 traits by bivariate LDSC (lower triangle) and by null Z-scores (upper triangle).

E.2 Results by Graph-MRcML-d0

As mentioned in the main text, given the presence of potential cycles in the underlying direct graph, the diagonal elements of the total graph may not be all zeros. So applying Graph-MRcML-d0 might be problematic. In particular, the final estimated direct graph by Graph-MRcML-d0 among the 17 traits had a spectral radius greater than one (so did every estimate from the 2000 perturbed datasets), violating the key assumption of the graph deconvolution algorithm, thus the result shown in Fig S was not trustworthy.

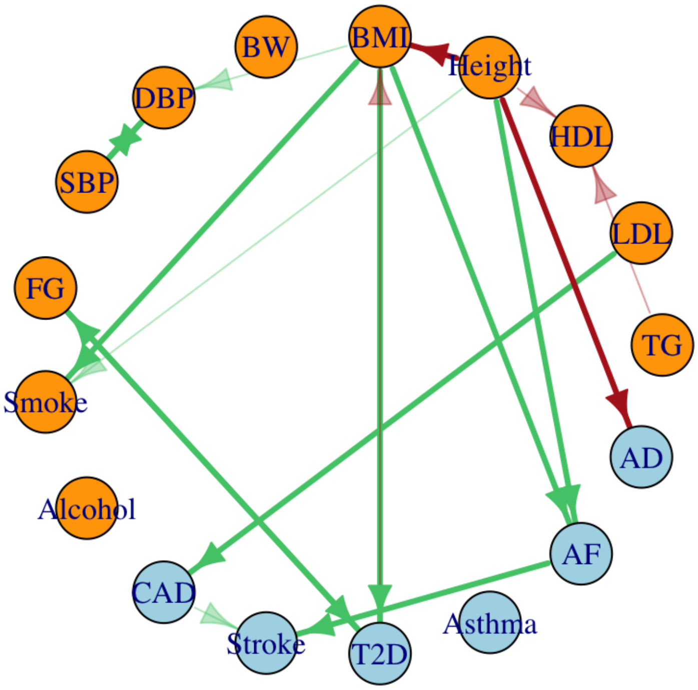

Fig S. Estimated direct graph by Graph-MRcML-d0. The spectral radius of the graph was greater than one. The dark-solid edges are identified at the Bonferroni-adjusted significance level, while the light-colored ones are marginally significant at a less stringent level of 6.5e-3.

We found that when we excluded traits with potential cycles, e.g., one of the two blood pressures traits (SBP and DBP), one of the two lipid traits (HDL and TG), and T2D, this spectral radius problem was much alleviated. For example, if we removed SBP, TG and T2D from the graph, the spectral radius of the final estimated direct graph among the 14 traits by Graph-MRcML-d0 was smaller than one, and so were those from all 2000 estimates. Furthermore, as shown in Fig T, the estimated direct graphs by Graph-MRcML-d0 (left) and Graph-MRcML-d1 (right) had similar structures. Again, we would like to point out that the problem was perhaps due to that Graph-MRcML-d0 simply specified the diagonal elements of the total graph to zeros while there were cycles in the underlying direct graph estimate (e.g. DBP $\leftrightarrow$ SBP, HDL $\leftrightarrow$ TG and T2D $\leftrightarrow$ FG, etc.). At the same time, it is also possible that the bi-directional MR analysis between DBP and SBP (and between HDL and TG, etc.) was not reliable as there were many shared common IVs between the two highly related traits, many of which might be invalid IVs that MR-cML-BIC-C failed to select out,

leading to biased estimation.

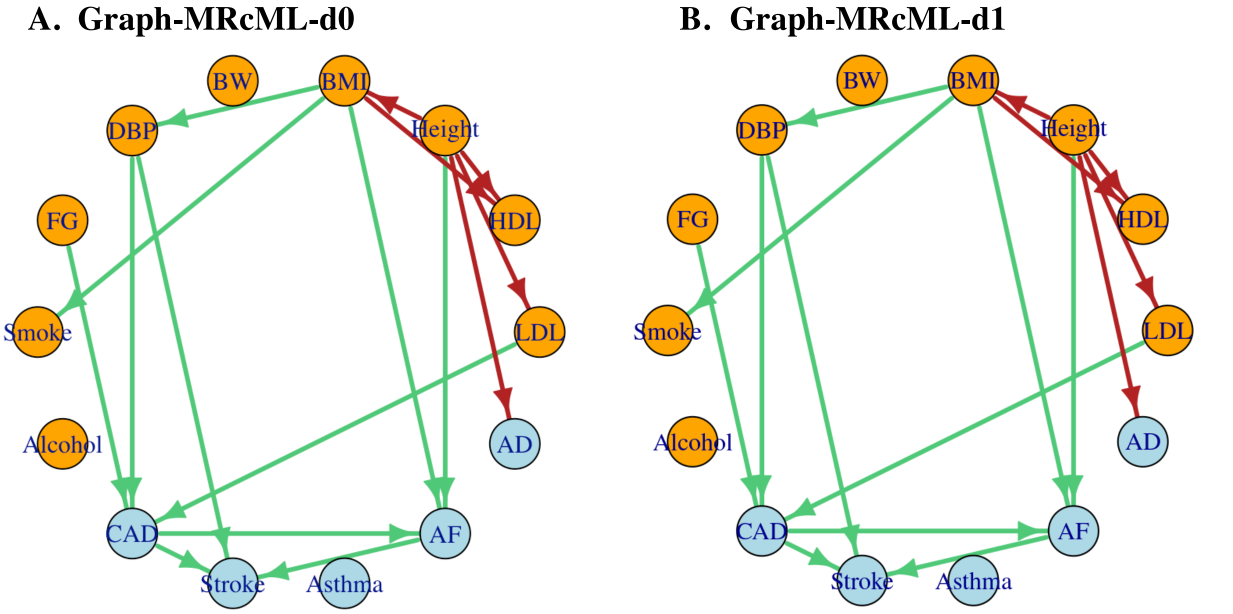

Fig T. Estimated direct graphs by Graph-MRcML-d0 (left) and Graph-MRcML-d1 (right) among 14 traits (after excluding SBP, TG and T2D).

E.3 More results on MVMR analysis

Table AH. Results for MVMR analysis estimating the direct effect of 15 exposures (excluding AD) on CAD.

			MVMR-IVW			MVMR-Robust		
Trait	F-statistics	#SNPs	effect size	SE	pval	effect size	SE	pval
FG	2.74	6	0.13	0.09	1.4e-1	0.14	0.10	1.5e-1
TG	10.87	22	0.15	0.05	2.0e-3	0.14	0.04	6.6e-4
LDL	9.98	23	0.38	0.04	1.4e-23	0.36	0.07	2.6e-7
HDL	19.33	39	-0.03	0.03	4.3e-1	-0.03	0.04	5.1e-1
Stroke	1.71	6	0.23	0.05	1.8e-5	0.18	0.06	2.2e-3
AF	6.29	29	0.11	0.02	2.0e-5	0.10	0.03	4.1e-4
Asthma	3.09	6	-0.01	0.03	7.9e-1	0.01	0.02	8.0e-1
BW	3.03	10	-0.06	0.09	4.9e-1	-0.04	0.07	5.9e-1
T2D	1.61	1	0.00	0.02	9.6e-1	0.00	0.02	8.7e-1
BMI	4.74	18	0.07	0.08	3.7e-1	0.12	0.07	6.1e-2
Height	7.64	194	-0.04	0.03	1.8e-1	-0.05	0.03	1.5e-1
Smoke	4.67	5	-0.00	0.05	1.0e+0	0.02	0.09	8.7e-1
Alcohol	3.00	6	-0.13	0.18	4.5e-1	-0.21	0.16	1.7e-1
DBP	9.66	35	0.27	0.11	1.2e-2	0.09	0.14	5.1e-1
SBP	5.85	33	0.15	0.12	2.4e-1	0.26	0.15	8.1e-2

Table AI. Results for MVMR analysis estimating the direct effect of 5 exposures on CAD.

			MVMR-IVW			MVMR-Robust		
Trait	F-statistics	#SNPs	effect size	SE	pval	effect size	SE	pval
TG	18.80	22	0.18	0.04	4.2e-5	0.18	0.03	1.5e-8
LDL	20.33	28	0.41	0.04	7.2e-27	0.41	0.08	6.3e-8
BMI	7.71	23	0.13	0.07	7.9e-2	0.20	0.06	6.0e-4
Height	36.50	245	-0.02	0.03	5.4e-1	-0.02	0.02	3.2e-1
SBP	7.95	34	0.51	0.09	3.2e-8	0.46	0.12	8.4e-5

E.4 Relationships among HDL, TG and glycemic traits

As pointed out by one reviewer, a recent study [21] identified strong causal effects of fasting insulin (FI) on HDL and TG, while we didn't observe any direct effect of fasting glucose (FG) on TG and HDL as shown in Fig 5B in the main text, and in a smaller network with the three traits of interest (Fig U Panel A). With an estimated number of effective test of

5, we identified a bi-directional effect between HDL and TG, and a negative effect of HDL on FG, at the Bonferroni-adjusted significance level (p-value < 0.01). We further performed analysis using FI, instead of FG, to study the causal relationships among HDL, TG and FI. Following [21], we obtained the GWAS summary data for FI from [22]. As shown in Fig U Panel B, we observed a significant positive effect of FI on TG and a negative effect on HDL, consistent with the findings in Figure 4B of [21]. We didn't identify any direct effect from HDL or TG to FI, while [21] also observed direct effects from HDL and TG to FI, though with a much smaller magnitude of effect size than those in the reverse direction (Figure 4A in [21]). This may partly due to the different GWAS summary data (with a smaller sample size) we used for lipid traits and different analysis approaches. We note that the FI GWAS summary data provided by [22] was adjusted for BMI, and using such GWAS data may introduce bias in the MR estimates [23]. To avoid such potential bias, we conducted an analysis using FI GWAS summary data without adjusting for BMI [24]. As shown in Fig U Panel C, the positive direct effect of FI on TG stayed significant (after the Bonferroni adjustment) while the negative effect of FI on HDL became only marginally significant with p-value ≈ 0.021. In summary, given the complex relationships between glycemic traits and lipid traits, more investigations are warranted.

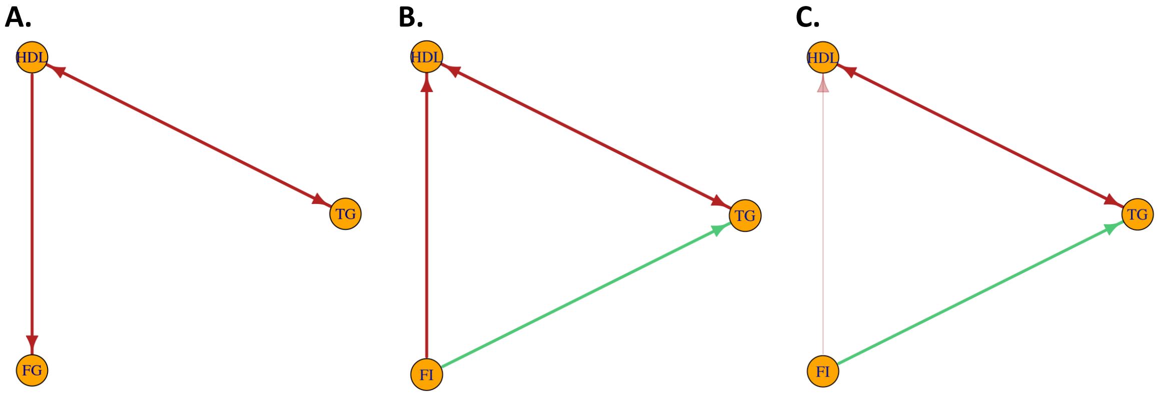

Fig U. Estimated direct causal networks for HDL, TG and (A) FG; (B) FI using BMI adjusted FI GWAS; (C) FI. The dark-solid edges are identified at the Bonferroni-adjusted significance level, while the light-colored ones are marginally significant at the nominal level of 0.05.

References

1. Xue H, Shen X, Pan W. Constrained maximum likelihood-based Mendelian randomization robust to both correlated and uncorrelated pleiotropic effects. The American Journal of Human Genetics. 2021;108(7):1251–1269.

2. Zhao Q, Wang J, Hemani G, Bowden J, Small DS. Statistical inference in two-sample summary-data Mendelian randomization using robust adjusted profile score. The Annals of Statistics. 2020;48(3):1742–1769.

3. Gentle JE. Matrix algebra. Springer texts in statistics, Springer, New York, NY, doi. 2007;10:978–0.

4. Boos DD, Stefanski LA. Essential statistical inference: theory and methods. vol. 591. Springer; 2013.

5. Xue H, Pan W. Robust inference of bi-directional causal relationships in presence of correlated pleiotropy with GWAS summary data. PLoS genetics. 2022;18(5):e1010205.

6. Hemani G, Tilling K, Davey Smith G. Orienting the causal relationship between imprecisely measured traits using GWAS summary data. PLoS genetics. 2017;13(11):e1007081.

7. Li MX, Gui HS, Kwan JS, Sham PC. GATES: a rapid and powerful gene-based association test using extended Simes procedure. The American Journal of Human Genetics. 2011;88(3):283–293.

8. Feizi S, Marbach D, Médard M, Kellis M. Network deconvolution as a general method to distinguish direct dependencies in networks. Nature biotechnology. 2013;31(8):726–733.

9. Willer CJ, Schmidt EM, Sengupta S, Peloso GM, Gustafsson S, Kanoni S, et al. Discovery and refinement of loci associated with lipid levels. Nature genetics. 2013;45(11):1274.

10. Wood AR, Esko T, Yang J, Vedantam S, Pers TH, Gustafsson S, et al. Defining the role of common variation in the genomic and biological architecture of adult human height. Nature genetics. 2014;46(11):1173–1186.

11. Locke AE, Kahali B, Berndt SI, Justice AE, Pers TH, Day FR, et al. Genetic studies of body mass index yield new insights for obesity biology. Nature. 2015;518(7538):197–206.

12. Horikoshi M, Beaumont RN, Day FR, Warrington NM, Kooijman MN, Fernandez-Tajes J, et al. Genome-wide associations for birth weight and correlations with adult disease. Nature. 2016;538(7624):248–252.

13. Dupuis J, Langenberg C, Prokopenko I, Saxena R, Soranzo N, Jackson AU, et al. New genetic loci implicated in fasting glucose homeostasis and their impact on type 2 diabetes risk. Nature genetics. 2010;42(2):105–116.

14. Liu M, Jiang Y, Wedow R, Li Y, Brazel DM, Chen F, et al. Association studies of up to 1.2 million individuals yield new insights into the genetic etiology of tobacco and alcohol use. Nature genetics. 2019;51(2):237–244.

15. van der Harst P, Verweij N. Identification of 64 novel genetic loci provides an expanded view on the genetic architecture of coronary artery disease. Circulation research. 2018;122(3):433–443.

16. Malik R, Chauhan G, Traylor M, Sargurupremraj M, Okada Y, Mishra A, et al. Multiancestry genome-wide association study of 520,000 subjects identifies 32 loci associated with stroke and stroke subtypes. Nature genetics. 2018;50(4):524–537.

17. Morris AP, Voight BF, Teslovich TM, Ferreira T, Segre AV, Steinthorsdottir V, et al. Large-scale association analysis provides insights into the genetic architecture and pathophysiology of type 2 diabetes. Nature genetics. 2012;44(9):981.

18. Demenais F, Margaritte-Jeannin P, Barnes KC, Cookson WO, Altmüller J, Ang W, et al. Multiancestry association study identifies new asthma risk loci that colocalize with immune-cell enhancer marks. Nature genetics. 2018;50(1):42–53.

19. Nielsen JB, Thorolfsdottir RB, Fritsche LG, Zhou W, Skov MW, Graham SE, et al. Biobank-driven genomic discovery yields new insight into atrial fibrillation biology. Nature genetics. 2018;50(9):1234–1239.

20. Jansen IE, Savage JE, Watanabe K, Bryois J, Williams DM, Steinberg S, et al. Genome-wide meta-analysis identifies new loci and functional pathways influencing Alzheimer's disease risk. Nature genetics. 2019;51(3):404–413.

21. Zhu Z, Wang K, Hao X, Chen L, Liu Z, Wang C. Causal graph among serum lipids and glycemic traits: a Mendelian randomization study. Diabetes. 2022;71(8):1818–1826.

22. Chen J, Spracklen CN, Marenne G, Varshney A, Corbin LJ, Luan J, et al. The trans-ancestral genomic architecture of glycemic traits. Nature genetics. 2021;53(6):840–860.

23. Hartwig FP, Tilling K, Davey Smith G, Lawlor DA, Borges MC. Bias in two-sample Mendelian randomization when using heritable covariable-adjusted summary associations. International journal of epidemiology. 2021;50(5):1639–1650.

24. Scott RA, Lagou V, Welch RP, Wheeler E, Montasser ME, Luan J, et al. Large-scale association analyses identify new loci influencing glycemic traits and provide insight into the underlying biological pathways. Nature genetics. 2012;44(9):991–1005.
